# Exploiting exciton coupling of ligand radical intervalence charge transfer transitions to tune NIR absorption†
†Electronic supplementary information (ESI) available: Full synthetic details, DPV data, supplementary EPR data, full UV-vis-NIR spectra for [**2˙˙**]^2+^, [**4˙˙**]^2+^, and [**5˙˙**]^2+^, 195 K UV-vis-NIR spectrum for [**5˙˙**]^2+^, DLS aggregation data for [**5˙˙**]^2+^, solvent dependence of [**5˙˙**]^2+^, DFT spin densities for triplet electronic structures, orbitals involved in the TD-DFT predicted transitions for triplet and broken symmetry solutions, optimized DFT coordinates, full TD-DFT output. CCDC 1579266–1579268. For ESI and crystallographic data in CIF or other electronic format see DOI: 10.1039/c7sc04537a


**DOI:** 10.1039/c7sc04537a

**Published:** 2017-12-19

**Authors:** Ryan M. Clarke, Tiffany Jeen, Serena Rigo, John R. Thompson, Loren G. Kaake, Fabrice Thomas, Tim Storr

**Affiliations:** a Department of Chemistry , Simon Fraser University , V5A1S6 , Burnaby , BC , Canada . Email: tim_storr@sfu.ca; b Départment de Chimie Moléculaire – Chimie Inorganique Redox (CIRE) – UMR CNRS 5250 , Université Grenoble-Alpes , B.P. 53 , 38041 Grenoble Cedex 9 , France

## Abstract

We detail the rational design of a series of bimetallic bis-ligand radical Ni salen complexes in which the relative orientation of the ligand radical chromophores provides a mechanism to tune the energy of intense intervalence charge transfer (IVCT) bands in the near infrared (NIR) region. Through a suite of experimental (electrochemistry, electron paramagnetic resonance spectroscopy, UV-vis-NIR spectroscopy) and theoretical (density functional theory) techniques, we demonstrate that bimetallic Ni salen complexes form bis-ligand radicals upon two-electron oxidation, whose NIR absorption energies depend on the geometry imposed in the bis-ligand radical complex. Relative to the oxidized monomer [**1˙**]^+^ (*E* = 4500 cm^–1^, *ε* = 27 700 M^–1^ cm^–1^), oxidation of the cofacially constrained analogue **2** to [**2˙˙**]^2+^ results in a blue-shifted NIR band (*E* = 4830 cm^–1^, *ε* = 42 900 M^–1^ cm^–1^), while oxidation of **5** to [**5˙˙**]^2+^, with parallel arrangement of chromophores, results in a red-shifted NIR band (*E* = 4150 cm^–1^, *ε* = 46 600 M^–1^ cm^–1^); the NIR bands exhibit double the intensity in comparison to the monomer. Oxidation of the intermediate orientations results in band splitting for [**3˙˙**]^2+^ (*E* = 4890 and 4200 cm^–1^; *ε* = 26 500 and 21 100 M^–1^ cm^–1^), and a red-shift for [**4˙˙**]^2+^ using *ortho*- and *meta*-phenylene linkers, respectively. This study demonstrates for the first time, the applicability of exciton coupling to ligand radical systems absorbing in the NIR region and shows that by simple geometry changes, it is possible to tune the energy of intense low energy absorption by nearly 400 nm.

## Abstract 2

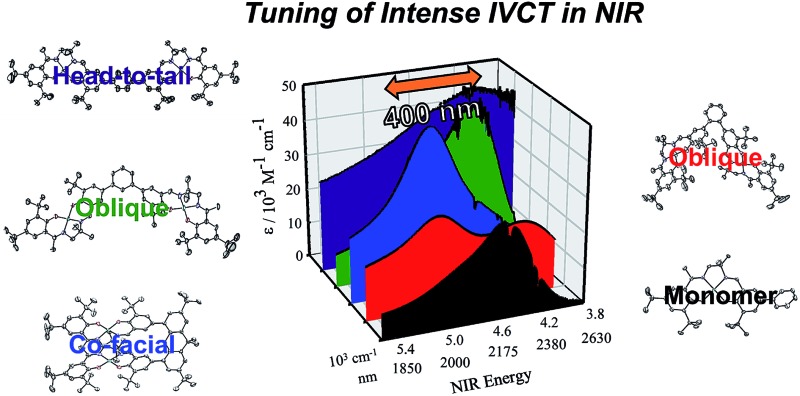
We detail the rational design of a series of bimetallic bis-ligand radical Ni salen complexes in which the relative orientation of the ligand radical chromophores provides a mechanism to tune the energy of intense intervalence charge transfer (IVCT) bands in the near infrared (NIR) region.

## Introduction

The photophysical properties of molecular aggregates continue to receive widespread attention due to their potential incorporation into display and wearable technologies,^1^ as well as photovoltaic devices.^2^ While the vast majority of molecular aggregates absorb in the visible region, there is significant interest in molecules that absorb in the near-infrared (NIR) due to the novel optical and electronic applications in this energy range.^3^ NIR absorbing materials include conjugated organic molecules and aggregates,^4^ polymers,^5^ transition metal complexes,^6^ and inorganic materials such as quantum dots^7^ and perovskites.^8^ Interestingly, mixed valence species can exhibit low energy intervalence charge transfer (IVCT) bands in the NIR, providing an additional class of low energy absorbing materials.^9^

**Fig. 1:**
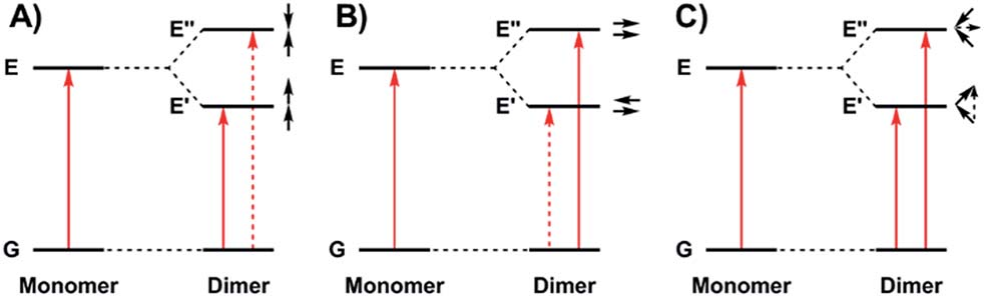
Exciton coupling of the excited states leads to band shifting and splitting relative to the analogous monomeric transition depending on the molecular geometry. Solid and dashed red lines represent allowed and forbidden transitions, respectively. Small black arrows represent transition moment dipoles.

The relationship between molecular packing and photophysical properties was eloquently described by Kasha, who showed that the alignment of transition moment dipoles and resulting coulombic interaction induces spectral shifts *via* exciton coupling when compared to uncoupled molecules (Fig. 1).^10^ For transition moment dipoles aligned in a head-to-tail fashion (Fig. 1A, J-aggregates), an in phase alignment of transition moment dipoles corresponds to a red-shifted band of double intensity relative to a monomeric analogue; while a blue-shifted band of double intensity is the result of in phase transition moment dipoles aligned in a cofacial manner (Fig. 1B, H-aggregates). Chromophores with transition moment dipoles oriented in an oblique arrangement will result in both red and blue-shifted bands as both orientations have net in phase alignment (Fig. 1C). Exciton coupling is common in the absorption spectra of aggregated dye molecules, and has also been well studied in *meso*-linked porphyrin arrays,^11^ and the π–π* transitions of dipyrinnato^12^ and azadipyrannato^13^ complexes. While the above description is sufficient in many cases, materials in alternate geometries and/or exhibiting short-range interactions (*i.e.* orbital overlap) have provided additional mechanisms to tune exciton coupling in molecular aggregates.^14^

IVCT bands in the NIR provide a sensitive probe of electron transfer, and Robin and Day provided a classification system for localized (Class I, where the valences cannot interconvert, and Class II where interconversion can occur under thermal or photochemical activation) and delocalized (Class III) mixed-valence species,^15^ with Hush providing a theoretical model for intervalence charge transfer in these species.^16^

**Fig. 2:**
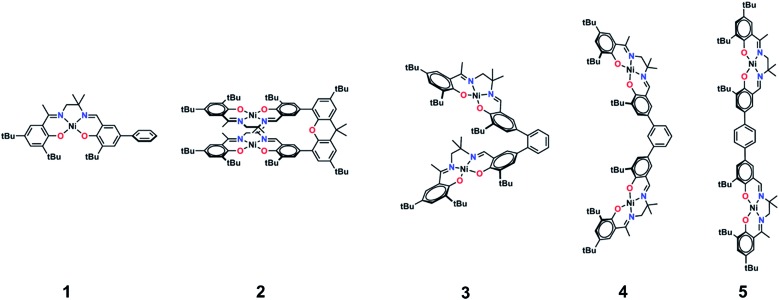
Monometallic and bimetallic salen complexes studied. **1** and **3** – previous work;^17^**2**, **4**, and **5** – this work.

We, and others, have investigated the relationship between electronic structure and NIR band energy and intensity in transition metal complexes employing pro-radical ligands.^17^ Alteration of ligand electronics,17c,j ligand chromophore orientation,17a,^18^ metal ion17g,^19^ and oxidation state17*h* are all important factors controlling the energy and intensity of the resulting NIR band in these systems. As a practical example, stable Ni(ii) dithiolene ligand radical complexes,^20^ which exhibit intense NIR absorptions, have been applied as low energy absorbers to limit the transmittance of NIR radiation from plasma displays,^21^ and incorporated into materials for electronic applications.^22^ One prevalent class of pro-radical ligand that has been investigated extensively are bis-imine bis-phenoxide complexes (*i.e.* salen) due to their facile and highly modular syntheses which allows for tuning of both the steric and electronic properties.^23^ For example, one-electron oxidation of the Ni salen complex **1** (Fig. 2) forms the ligand radical [**1˙**]^+^, which exhibits a sharp, intense IVCT band in the NIR (*E* = 4500 cm^–1^, *ε* = 27 700 M^–1^ cm^–1^, Δ*ν*_1/2_ = 650 cm^–1^), indicative of a Class III fully delocalized ligand radical in the Robin–Day classification system.17*a* The ligand radical [**1˙**]^+^ is stable for weeks in CH_2_Cl_2_ solution and in a PMMA film, and does not show any concentration-dependent aggregation effects in solution. We were intrigued to further consider the applicability of oxidized Ni salens, and tunability of the intense NIR absorption, for NIR absorbing materials. We have now investigated a series of salen dimers **2–5** (Fig. 2) to probe salen radical excited-state electronic coupling.

Further interest in multimetallic Schiff-base complexes arises from their interesting reactivity,^24^ electronic structure,^25^ magnetism,^26^ and supramolecular properties.^27^ In related work, we have investigated the synthesis and oxidation of multimetallic salen complexes for potential catalytic applications.17*a* While pursuing this work, we synthesized **3** and investigated the doubly oxidized form [**3˙˙**]^2+^.17*a* The NIR region of the absorption spectrum for [**3˙˙**]^2+^ exhibited two intense NIR bands, equally spaced at higher and lower energies to the monomer [**1˙**]^+^. While we could not discount the possibility that the observed band splitting was due to different conformers of [**3˙˙**]^2+^, our analysis suggested that the NIR band splitting could be accounted for by exciton coupling. Related work in this area has demonstrated the interesting intermolecular interactions and resulting photophysical properties of salen systems.^28^

We now provide a full experimental and theoretical study of exciton coupling in a series of bimetallic bis-ligand radical complexes, where the distance and orientation of the NIR chromophores are varied systematically (Fig. 2). The resulting analysis supports exciton coupling of the intense NIR bands, providing a mechanism to tune the energy of the intense ligand radical NIR absorption in this system.

## Results

### Synthesis and solid-state characterization of bimetallic Ni complexes

**Fig. 3:**
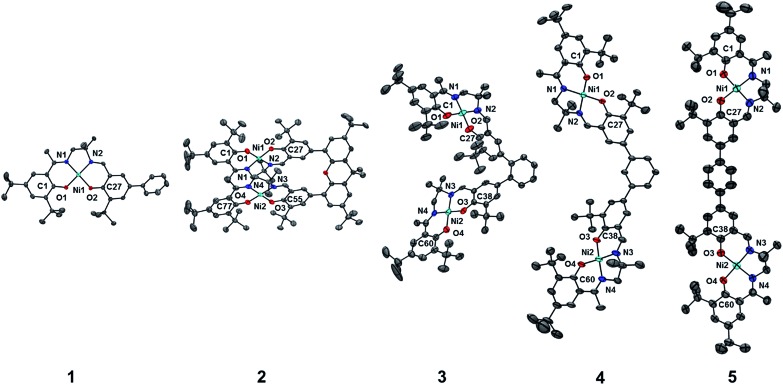
ORTEPs of **1–5** (50% probability) generated using POV-Ray, excluding hydrogen atoms and solvent. See Tables S1 and S2† for metrical parameters and crystallographic details, respectively.

The synthetic strategies to form ligands (H_4_L^2^, H_4_L^4^ and H_4_L^5^) and complexes are presented in Schemes S1–S3.† All three ligands are synthesized *via* Suzuki coupling of the corresponding linking unit and a salicylaldehyde boronate ester, followed by condensation with 2 equivalents of a ‘half-salen’ unit. Complexes **3**, **4** and **5** alternate substitution patterns around the phenylene linker (*ortho*, *meta*, and *para* respectively), while we employed the xanthene spacer in **2**, which has been used by Nocera to cofacially align porphyrin rings for dioxygen activation reactions,^29^ and Wasielewski for the alignment of organic chromophores.^30^ The solid-state structures of **1–5** are presented in Fig. 3. The Ni N_2_O_2_ coordination sphere in all complexes is slightly distorted from a square planar geometry, with the distortion likely due to the sterically demanding *t*Bu substituents in the *ortho* positions. DFT calculations on **2**, **4** and **5** well reproduce the coordination sphere bond lengths of the three complexes, with the coordination sphere metrical parameters replicated to within ±0.02 Å of the experimental values (Table S1†). Furthermore, the calculations also accurately reproduce the slight asymmetry in the coordination spheres due to the asymmetric salen ligand. The individual Ni salen moieties are oriented in a *trans* conformation relative to one another in the solid-state in **2**, with the sterically-demanding *t*Bu substituents facing away from each other. This is in contrast to the previously reported *cis*-orientation for **3** in the solid state (Fig. 3), however in solution both conformers were observed by VT ^1^H NMR with a DFT-computed rotational barrier of *ca.* 6 kcal mol^–1^.17*a* DFT calculations on **2** predict the *trans* conformation to be *ca.* 2.7 kcal mol^–1^ lower in energy in comparison to the *cis* conformation (*vide infra*). Additionally, the close spatial proximity between the salen moieties enforced by the xanthene spacer (Ni···Ni distance of 3.98 Å) prohibits rotation of the salen units, and thus **2** is locked in a *trans* conformation at room temperature. The recrystallized *trans* form of **2** was used in all subsequent experiments. The angle between the salen planes in **2** is only 8.2°, highlighting the essentially cofacial arrangement of the salen units (*vide infra*).

Similarly to **2**, **4** adopts a *trans* conformation of the salen units in the solid-state, although DFT calculations predict both *cis* and *trans* conformers to be essentially isoenergetic (difference of *ca.* 0.11 kcal mol^–1^). The observed solid-state *trans* conformation is likely a result of solid-state packing effects and there is expected to be a relatively small rotational barrier of the salen units in solution. Finally, the solid-state structure of **5** adopts a *cis* conformation of the salen units, in similar fashion to **3**. DFT calculations predict both *cis* and *trans* conformers to be essentially isoenergetic (difference of *ca.* 0.09 kcal mol^–1^). Overall the X-ray results show the expected configuration of the salen units in **2–5** as mediated by the linker, and steric interactions responsible for the restricted rotation of the salens observed for **2** and **3**.

### Electrochemistry

**Fig. 4:**
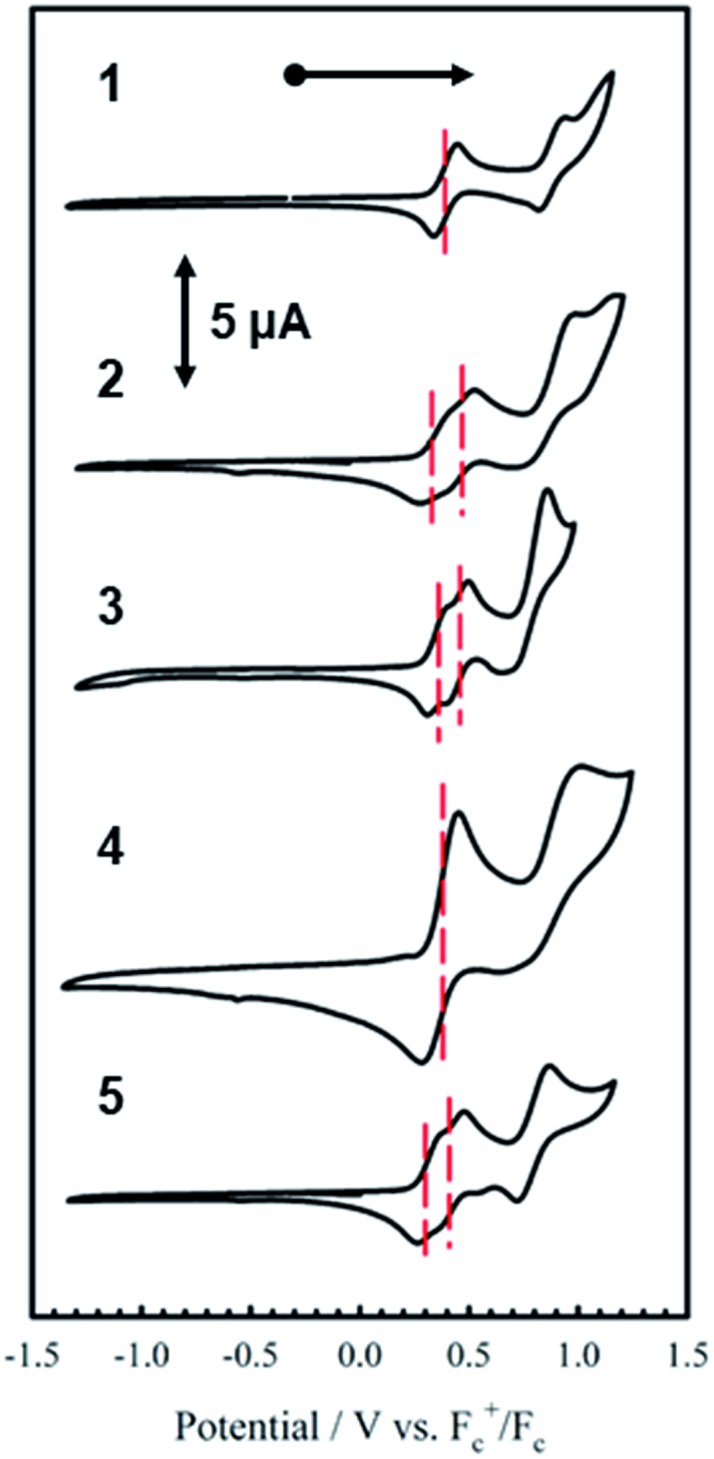
Cyclic voltammograms of **1–5** (top to bottom). Conditions: 1 mM complex, 0.1 M ^*n*^Bu_4_ClO_4_, scan rate 100 mV s^–1^, CH_2_Cl_2_, 230 K.

The electrochemical behavior of new bimetallic Ni salen complexes **2**, **4** and **5** was studied by cyclic voltammetry (CV) in CH_2_Cl_2_ at 230 K and compared to the electrochemical behavior of **1** and **3** (Fig. 4).17*a* All three new complexes are easily oxidized in similar ranges to the previously studied **1** and **3**, reflecting the common oxidation of the Ni salen units to form a delocalized ligand radical. Both **2** and **5** exhibit two closely-spaced reversible one-electron redox couples at *E*_1/2_ = 330 mV and *E*_1/2_ = 470 mV for **2** and *E*_1/2_ = 300 mV and *E*_1/2_ = 410 mV for **5**; as well as irreversible oxidation events at approximately 800–850 mV (Table 1). In contrast to the closely spaced first two oxidation events for **2** and **5**, **4** displays only one reversible oxidation event at low potential (*E*_1/2_ = 380 mV), albeit a two-electron process reflected in a doubling of the current intensity in comparison to **2**, **3** and **5**, which is further emphasized in the differential pulse voltammetry (DPV) data (Fig. S1†).^31^ This is likely due to the *meta* 1,3-phenylene linker in **4** in comparison to the *ortho* and *para* connectors in **3** and **5** which facilitate communication between the salen units through the bridge.^32^ The splitting of the first two redox processes in **2** is therefore attributable to a small through space interaction between the redox-active salen moieties dictated by the xanthene spacer. The electronic coupling between the two redox-active salen moieties in [**2˙˙**]^2+^, [**4˙˙**]^2+^ and [**5˙˙**]^2+^ was investigated *via* the difference between the first and second oxidation events (Δ*E*_ox_) and the comproportionation constant, *K*_c_ [eqn (1)–(3)].^33^ The small separation between the first two redox events in **2** and **5**, as well as the corresponding values of *K*_c_ suggests limited coupling between the two redox-active salen moieties, and that each salen unit is oxidized individually. In comparison, the unresolvable value of Δ*E*_ox_ for **4** suggests negligible coupling in this derivative.

**Table 1:** Redox potentials for **1–5***vs.* Fc^+^/Fc^*a*^ (1 mM complex, 0.1 M ^*n*^Bu_4_NClO_4_, scan rate 100 mV s^–1^, CH_2_Cl_2_, 230 K), and the comproportionation constant (*K*_c_)^*c*^

	E_1/2_^1^/mV	*E*_1/2_^2^/mV	*E*_1/2_^3^/mV	Δ*E*_ox_ (*E*_1/2_^2^ – *E*_1/2_^1^)	*K*_c_ (230 K)^*b*^
**1**	390 (106)	890 (116)	—	500	9.04 × 10^10^
**2**	330 (115)	470 (110)	850 (230)	140	1.17 × 10^3^
**3**	360 (100)	460 (100)	800 (200)	100	1.55 × 10^2^
**4**	380 (140)	—	875 (210)	495	—
**5**	300 (90)	410 (96)	795 (150)	110	2.57 × 10^2^

^*a*^Peak-to-peak differences in brackets (|*E*_pa_ – *E*_pc_| in mV). Peak-to-peak difference for the Fc^+^/Fc couple at 230 K is 110 mV.

^*b*^ *K*_c_ for **1** is within a salen unit, while *K*_c_ for **2–5** is between salen fragments.

^*c*^ Ref. 17.

The *K*_c_ values for **2**, **3**, and **5** are much smaller than the value for **1** (Table 1), which corresponds to the coupling between the redox-active phenolates within a salen unit. While not further investigated, we presume the redox events at high potential for **2–5** are due to the formation of bis-ligand radical species on the salen units.^34^1
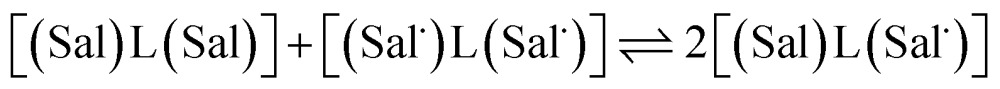

2
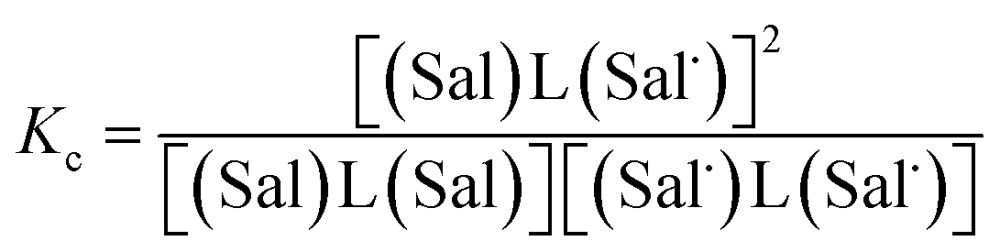

3
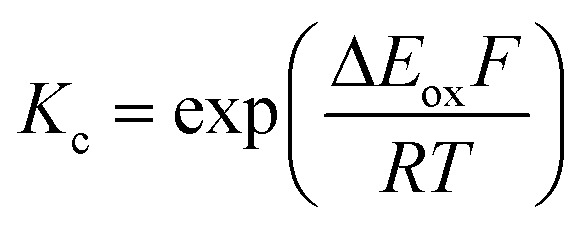



### Electron paramagnetic resonance spectroscopy (EPR)

EPR is an effective tool to study the degree of delocalization, and metal participation in the SOMO, in transition-metal complexes incorporating redox-active ligands. In this work, EPR is a sensitive probe for detecting spin–spin interactions in the bis-ligand radical species [**2–5˙˙**]^2+^.

**Fig. 5:**
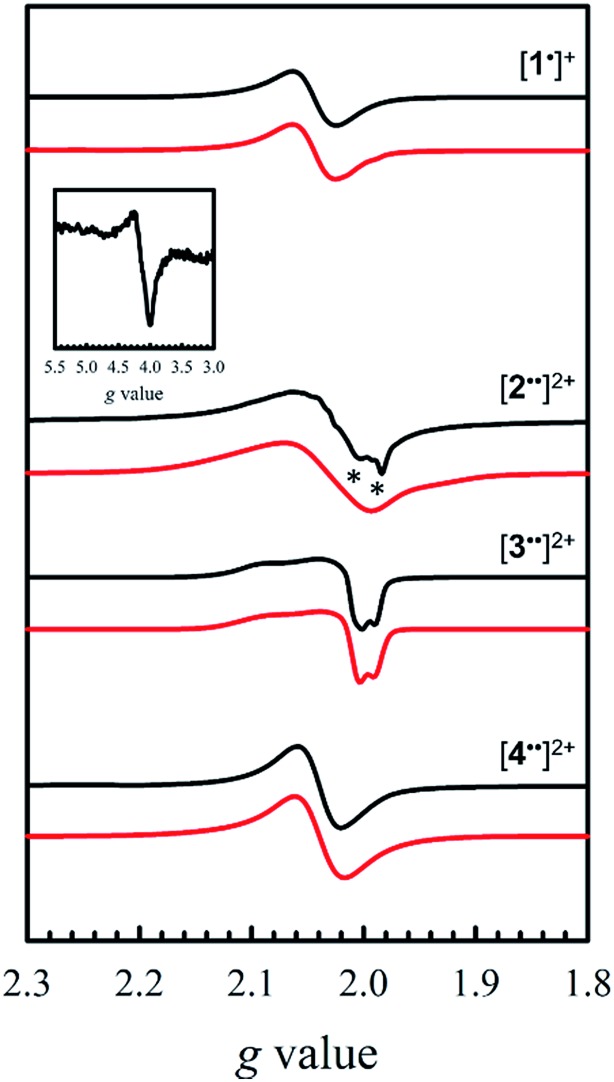
X-band EPR spectra (black) of [**1˙**]^+^ and [**2–4˙˙**]^2+^ recorded in frozen CH_2_Cl_2_ at 0.33 mM. Red lines represent simulations to the experimental data. Inset: weak half-field Δ*M*_s_ = 2 transition for [**2˙˙**]^2+^. Conditions: frequency = 9.38 GHz ([**1˙**]^+^ and [**3˙˙**]^2+^), 9.64 GHz ([**2˙˙**]^2+^ and [**4˙˙**]^2+^); power = 2 mW; modulation frequency = 100 kHz; modulation amplitude = 0.4 mT; *T* = 6 K. Asterisks denote the monoradical [**2˙**]^+^ impurity.

**Fig. 6:**
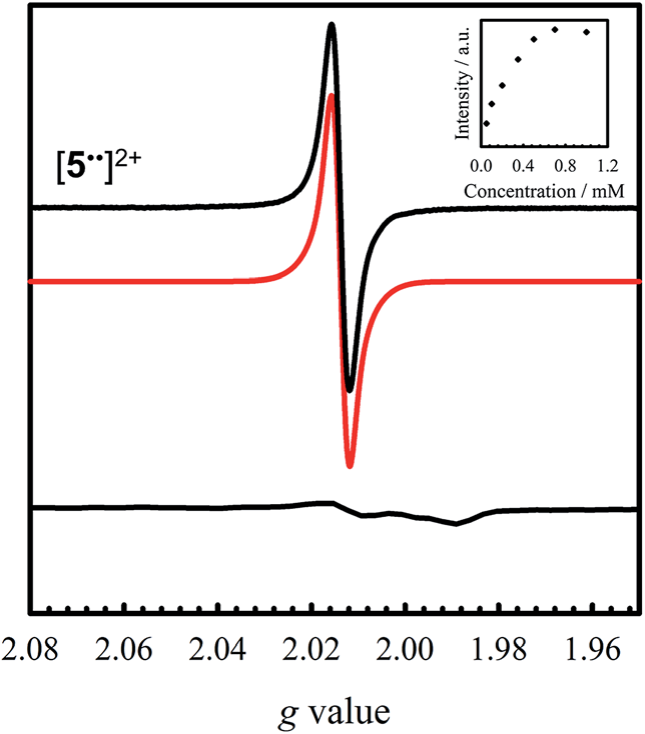
Room temperature X-band EPR spectrum of [**5˙˙**]^2+^ (black) recorded in CH_2_Cl_2_ at 0.35 mM (simulation in red). Inset: saturation curve of the signal at *g* = 2.014 with increasing concentration suggesting aggregation above 0.5 mM. Conditions: frequency = 9.44 GHz; power = 2 mW; modulation frequency = 10 kHz; modulation amplitude = 0.15 mT; *T* = 298 K. Bottom: X-band EPR spectrum of [**5˙˙**]^2+^ oxidized at 77 K. Conditions: frequency = 9.38 GHz; power = 2 mW; modulation frequency = 100 kHz; modulation amplitude = 0.6 mT.

The X-band EPR spectra of [**1˙**]^+^ and [**2–4˙˙**]^2+^ are presented in Fig. 5, and that of [**5˙˙**]^2+^ is shown in Fig. 6. All five complexes exhibit *g* values close to that of the free electron, indicating ligand radical species (Table 2). The *g* values are slightly higher than expected for purely ligand based radicals (*g*_ave_ > *g*_e_ ∼ 2.0023), which suggests non-negligible contribution of the Ni centers to the SOMOs. The ligand radical [**1˙**]^+^ was previously investigated and exhibits a rhombic spectrum (*g*_ave_ = 2.048, Table 2) with *ca.* 13% Ni d_*xz*_ character in the SOMO (based on DFT).17*a*

**Table 2:** EPR simulation parameters for the complexes

Complex	*g*_*xx*_	*g*_*yy*_	*g*_*zz*_	*g*_ave_	*D*/10^–4^ cm^–1^	*E*/*D*
[**1˙**]^+^^*a*^	2.061	2.046	2.037	2.048	—	—
[**2˙˙**]^2+^	2.120	2.020	1.980	2.035	75	<0.05
[**3˙˙**]^2+^	2.090	2.019	2.000	2.036	13	<0.04
[**4˙˙**]^2+^	2.047	2.036	2.033	2.038	—	—
[**5˙˙**]^2+^	2.014				—	—

^*a*^ Ref. 17.

The X-band EPR spectrum of [**2˙˙**]^2+^ differs substantially from that of [**1˙**]^+^, exhibiting a low field resonance at *g* = 4 in addition to the features at *ca. g* = 2. This low field resonance is attributed to a half-field transition (Fig. 5 – inset), which suggests significant population of the triplet spin state. Both the weak intensity and position suggest that the zero-field splitting (ZFS) parameters are small.^35^ The signal at *ca. g* = 2 contains two contributions. The minor component originates from an *S* = 1/2 monoradical [**2˙**]^+^ impurity,^36^ which is manifest as a relatively sharp rhombic feature at *g*_1_ = 2.115, *g*_2_ = 2.012, and *g*_3_ = 1.987 (Fig. S2†). The main spectrum is much broader and displays outermost resonances assigned to the Δ*M*_s_ = 1 transitions of the triplet species.^37^ The low resolution of the triplet spectrum complicates the determination of accurate ZFS parameters by simulation. By using the *g* values determined for [**2˙**]^+^ as an initial guess for the simulation of the triplet spectra at both X- and Q-band frequencies (Fig. S3†), the ZFS parameters are estimated as |*D*| = 0.0075 cm^–1^ with an *E*/*D* < 0.05. By using the point dipole approximation these ZFS parameters afford an interspin distance of 6.9 Å, which exceeds the distance between the two mean N_2_O_2_ planes measured in the crystal structure of **2** (3.98 Å). Such discrepancy is not unexpected for organic diradicals and can be taken as evidence of substantial delocalization of the unpaired electrons.^38^ We further investigated the magnetic interaction in [**2˙˙**]^2+^*via* the temperature dependence of the Δ*M*_s_ = 2 signal. A plot of the intensity of the Δ*M*_s_ = 2 signal as a function of 1/T results in a straight line (Fig. S4†), suggesting a weak interaction between the two salen radical units. The magnetic coupling for [**2˙˙**]^2+^ is comparable to that reported for paracyclophane,^39^ and Ni(ii)-thiazyl p-stacked radical dimers,^40^ and could be further interpreted in terms of a McConnel-I mechanism involving delocalized radicals.^41^

The EPR spectrum for [**3˙˙**]^2+^ was previously simulated as a biradical^42^ by considering the individual salen moieties in *cis* and *trans* conformations relative to one another, with the observed experimental data a combination of the two sub-spectra.17*a* Although we do not observe a half-field transition, based on the analysis of [**2˙˙**]^2+^, the data for [**3˙˙**]^2+^ was simulated here as a triplet species instead of two conformers, with small ZFS parameters (Table 2). The lack of a half-field transition, coupled with the small ZFS parameters suggest limited coupling in [**3˙˙**]^2+^.^42^ Using the point dipole approximation, an interspin distance is calculated as 12.5 Å. This value is again larger than the 9.8 Å distance between the Ni centers in the crystal structure of **3**, and similarly to the analysis of [**2˙˙**]^2+^, suggests extensive delocalization of the unpaired electrons. It must be stressed that the D values are small for both [**2˙˙**]^2+^ and [**3˙˙**]^2+^, confirming that the spin–spin interactions are weak in both complexes. The EPR spectrum of [**4˙˙**]^2+^ exhibits a broad signal at *g*_ave_ = 2.038, consistent with the other oxidized complexes. We do not observe a half-field transition, likely due to the large separation between the salen moieties in [**4˙˙**]^2+^. In agreement with the above structural and electrochemical data, the EPR spectrum of mono-oxidized [**4˙**]^+^ (Fig. S5†) is very similar to [**4˙˙**]^2+^, but with *ca.* 1/2 the intensity.

Oxidation of **5** to [**5˙˙**]^2+^ at low temperature resulted in a very weak EPR signal (Fig. 6 – bottom) with *ca.* 3% intensity in comparison to a concentration-matched sample of [**4˙˙**]^2+^. We hypothesized that the lack of signal was either due to sample decomposition, or spin–spin interactions due to intermolecular interaction of [**5˙˙**]^2+^ at low temperature (*vide infra*). EPR analysis of a solution of [**5˙˙**]^2+^ at 298 K affords an isotropic signal with a *g*_iso_ = 2.014, supporting the assignment as a bis-ligand radical species, although with less contribution of the central Ni atoms to the SOMO. The intensity of the doubly integrated signal of [**5˙˙**]^2+^ under non-saturating conditions is plotted against the concentration in the inset of Fig. 6. A clear saturation is observed at *ca.* 0.5 mM, indicative of dimerization/aggregation processes above this concentration.

### Vis-NIR spectroscopy

**Fig. 7:**
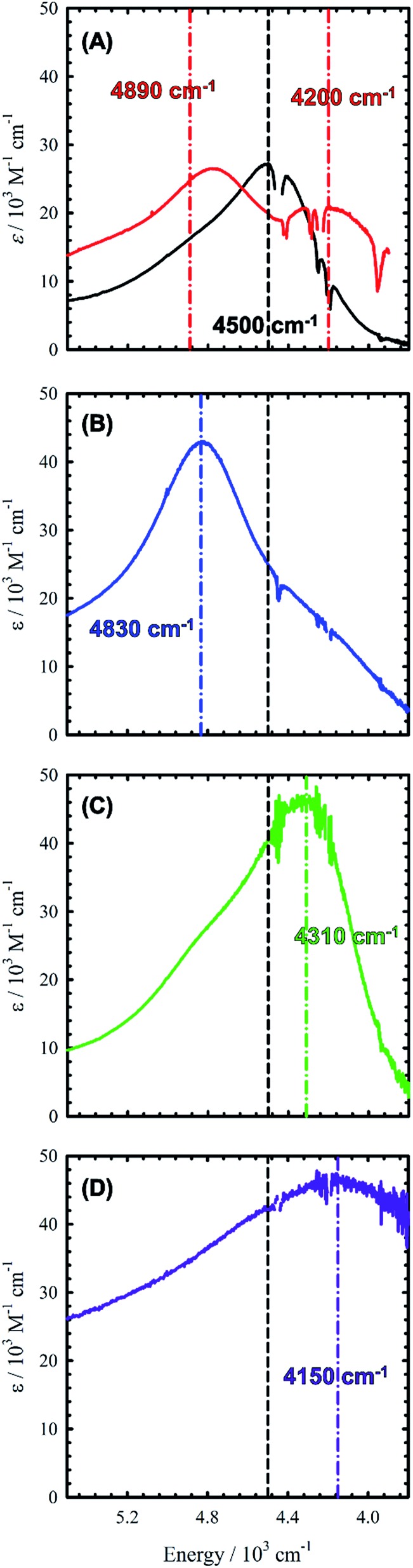
NIR region in the absorption spectra of [**1˙**]^+^ and [**2–5˙˙**]^2+^. (A) Black line: [**1˙**]^+^, red line: [**3˙˙**]^2+^; (B) [**2˙˙**]^2+^; (C) [**4˙˙**]^2+^; (D) [**5˙˙**]^2+^. The dashed black line represents *λ*_max_ for [**1˙**]^+^. Colored dashed lines represent *λ*_max_ for the respective bis-ligand radical complex. Conditions: CH_2_Cl_2_, 0.33 mM complex (A–C), 0.08 mM complex (D), *T* = 195 K (A–C), 298 K (D).

Chemical oxidation of **2**, **4** and **5** with two equivalents of [N(C_6_H_3_Br_2_)_3_˙]^+^[SbF_6_]^–^ at 198 K results in the appearance of intense transitions in the NIR region of the absorption spectra (Fig. 7 and Table 3). In all cases, isosbestic points are observed during the sequential addition of oxidant from 0–2 equivalents indicating clean conversion to the respective oxidized product (see Fig. S6–S8† for full UV-vis-NIR spectra). For [**2˙˙**]^2+^, in which the salen moieties are cofacially aligned the exciton model predicts a blue-shifted and doubly intense band relative to the monomeric analogue [**1˙**]^+^. We indeed observe a blue-shifted band by *ca.* 330 cm^–1^ relative to the IVCT transition observed in [**1˙**]^+^ (Fig. 7A and B). The IVCT band for [**2˙˙**]^2+^ is nearly double the intensity of the band observed in [**1˙**]^+^ (42 900 *vs.* 27 700 M^–1^ cm^–1^), with the slightly diminished intensity in [**2˙˙**]^2+^ likely due to minimal symmetry perturbations resulting in the low energy forbidden transition gaining weak intensity.^43^ Indeed, the low energy transition in [**2˙˙**]^2+^ is evident as a shoulder in Fig. 7B. For oblique systems such as [**3˙˙**]^2+^ and [**4˙˙**]^2+^, the exciton model predicts a splitting of bands as both transitions at high and low energy are partially allowed (Fig. 1). The intensity of the observed bands is dependent on the distance and angle between the transition dipole moments. For [**3˙˙**]^2+^, which has an approximate angle of 60° between the two salen units, two NIR transitions are observed equally split about the IVCT band in [**1˙**]^+^.17*a* The slight increase in intensity for the higher energy band for [**3˙˙**]^2+^ is in agreement with the Kasha model.10*b* For [**4˙˙**]^2+^ which has a significantly larger angle between the two salen units (120°) and is closer to the parallel system, we observe a prominent low energy band red-shifted by *ca.* 190 cm^–1^ as well as a lower-intensity shoulder corresponding to the higher energy transition predicted by the exciton model (Table 3).

**Table 3:** Spectroscopic properties of the Ni complexes in CH_2_Cl_2_ solution^*a*^

Complex^*b*^	*λ*_max_ [cm^–1^] (*ε* × 10^3^ [M^–1^ cm^–1^])
**1** ^*c*^	25 400 sh (4.7), 23 900 (8.2), 22 300 sh (5.3), 17 400 w (0.36)
[**1˙**]^+^^*c*^	32 000 (100), 26 500 (14.6), 25 000 (10.6), 21 700 (7.1), 19 100 (4.1), 11 000 (3.0), 9100 (9.2), 5900 sh (7.1), **4500 (27.7)**
**2**	23 800 (15.6), 21 800 sh (8.6), 16 700 w (1.7)
[**2˙˙**]^2+^	21,00 sh (7.2), 17 800 (3.4), 12 100 (5.8), 8970 (12.3), **4830 (42.9)**
**3** ^*c*^	23 800 (13.4), 22 300 sh (9.1), 17 400 w (0.42)
[**3˙˙**]^2+^^*c*^	32 000 (100), 26 500 (19.7), 25 000 (16.4), 21 700 (9.9), 19 100 (4.8), 9260 (10.4), **4890 (26.5), 4200 (21.1)**
**4**	23 500 (14.5), 21 850 sh (8.8), 17 500 w (0.8)
[**4˙˙**]^2+^	21 500 (9.5), 18 500 (4.4), 9100 (14.0), 5850 sh (8.2), **4850 sh (26.1), 4310 (46.1)**
**5**	23 500 (14.4), 21 600 sh (9.2), 17 400 w (1.1)
[**5˙˙**]^2+^	20 200 (28.6), 8700 sh (8.0), 5400 sh (27), **4150 (46.6)**

^*a*^NIR transitions in bold.

^*b*^Conditions: 0.33 mM (**1–4**), 0.08 mM (**5**), CH_2_Cl_2_, 198 K (**1–4**), 298 K (**5**); sh = shoulder, w = weak.

^*c*^ Ref. 17.

Finally, the exciton model predicts that [**5˙˙**]^2+^ will afford a red-shifted band relative to the monomeric analogue. At low temperature, we do not observe a red-shifted IVCT band, but a broad transition ranging from 20 000 cm^–1^ to 10 000 cm^–1^ (*ε* = 30 000 M^–1^ cm^–1^), alongside a broad blue-shifted NIR band (*E* = 5100 cm^–1^, *ε* = 29 000 M^–1^ cm^–1^) (Fig. S9†). Heating/sonication of this solution does not change the absorption profile and dynamic light scattering (DLS) experiments confirm the presence of large aggregates (mean size ∼145 nm – Fig. S10†), likely formed *via* π-interactions between radical cations.^44^ Oxidation of a dilute solution (0.08 mM) of **5** to [**5˙˙**]^2+^ at 298 K does however afford a red-shifted band as predicted by the exciton model (Fig. 7D). Concentration-dependent EPR studies at 298 K show saturation of the main resonance at *g* = 2 beginning at 0.5 mM, suggesting aggregation above this concentration, and the broadness of the band depicted in Fig. 7 is likely due to partial aggregation of [**5˙˙**]^2+^ even at room temperature (DLS mean aggregate size ∼38 nm, Fig. S10†). The ‘monomeric’ form of [**5˙˙**]^2+^ is favoured in toluene, however, the oxidized complex displays limited stability in this solvent medium (Fig. S11–S14†). Overall, the shifting and/or splitting of the intense IVCT bands in the bimetallic bis-ligand radical species is correctly predicted by the exciton model.

### Theoretical characterization of [**2˙˙**]^2+^, [**4˙˙**]^2+^, and [**5˙˙**]^2+^

**Fig. 8:**
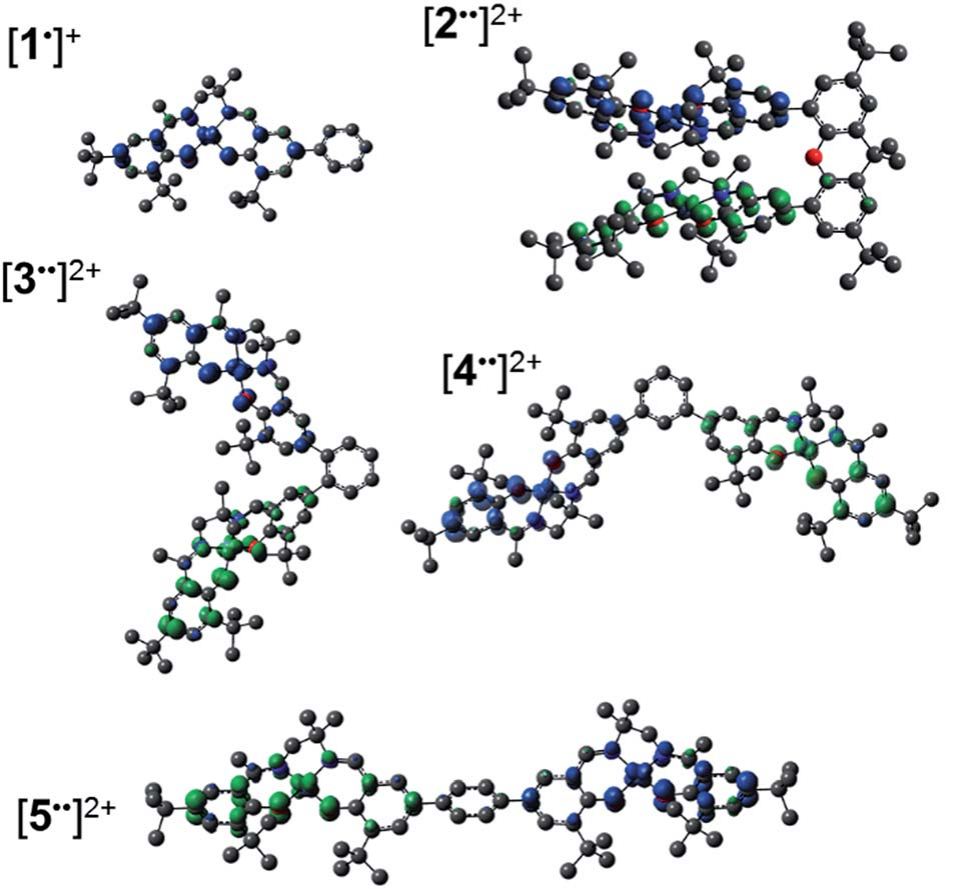
Spin density plots for [**1˙**]^+^ and the broken symmetry (*S* = 0) solution for [**2–5˙˙**]^2+^. See the experimental section for calculation details.

Geometry optimization for both triplet (*S* = 1) and broken symmetry (BS, *S* = 0) electronic structures for [**2˙˙**]^2+^, [**4˙˙**]^2+^ and [**5˙˙**]^2+^ show a pronounced coordination sphere contraction when compared to the neutral analogues (Table S1†), matching the predicted coordination sphere changes for [**1˙**]^+^ and [**3˙˙**]^2+^.17*a* The two spin states are nearly isoenergetic for both [**4˙˙**]^2+^ (BS lower in energy by 0.2 kcal mol^–1^) and [**5˙˙**]^2+^ (triplet lower in energy by 0.05 kcal mol^–1^). Interestingly, the BS solution for [**2˙˙**]^2+^ is 2.8 kcal mol^–1^ lower in energy in comparison to the triplet solution, thus predicting the most significant spin-interaction for this species. In all cases, the spin density plots show that the radicals are delocalized across the salen moieties, with minimal spin density on the bridging unit (Fig. 8/Fig. S15†).

**Fig. 9:**
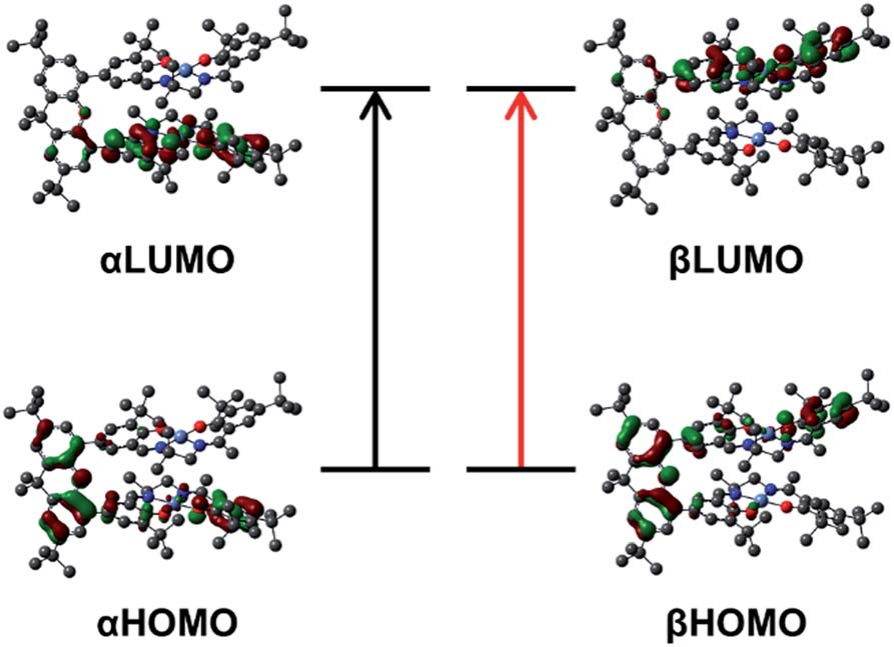
Kohn–Sham molecular orbitals for the broken-symmetry solution (*S* = 0) of [**2˙˙**]^2+^ associated with the calculated NIR transitions at 4995 and 3590 cm^–1^. The predicted low energy bands are symmetric and antisymmetric linear combinations of the αHOMO → αLUMO (black arrow), and βHOMO → βLUMO (red arrow) local transitions on the individual salen radicals.

Time-dependent DFT (TD-DFT) calculations on both triplet and BS solutions for the bis-ligand radical species were employed to gain insight into the donor and acceptor orbitals, and energy shifts associated with the experimentally observed NIR features. The TD-DFT results are similar for both electronic structures, predicting low energy transitions with an intensity pattern in line with the exciton model (Table 4). For the triplet calculations (Fig. S15† for spin densities), extensive mixing of the donor and acceptor orbitals results in orbitals delocalized over both salen units (Fig. S16–S18†). For the BS calculations, the donor and acceptor orbitals are confined to the individual salen units and match the orbitals associated with the predicted low energy transition for the monomer [**1˙**]^+^ (Fig. 9, S19 and S20†).17*a* The predicted low energy bands from the BS TD-DFT calculations are symmetric and antisymmetric linear combinations of the αHOMO → αLUMO and βHOMO → βLUMO local transitions on the individual salen radicals, providing further evidence of excited-state mixing of the salen chromophores in the doubly oxidized dimers.

**Table 4:** TD-DFT predicted energies and oscillator strengths for the broken symmetry (*S* = 0) solutions for the doubly oxidized dimers.^*a*^ ^,^^*b*^ ^,^^*c*^

	Low energy band		High energy band	
	Energy (cm^–1^)	Oscillator strength (*f*)	Energy (cm^–1^)	Oscillator strength (*f*)
[**2˙˙**]^2+^	3590	0.055	**4995**	**0.406**
[**3˙˙**]^2+^	**4708**	**0.270**	**5675**	**0.196**
[**4˙˙**]^2+^	**4935**	**0.395**	5570	0.092
[**5˙˙**]^2+^	**4390**	**0.744**	5495	0.002

^*a*^See the Experimental section for calculation details.

^*b*^The TD-DFT predicted NIR band for [**1˙**]^+^ is at *E* = 5260 cm^–1^, *f* = 0.2450.

^*c*^The predicted band energies for the triplet (*S* = 1) solutions match the BS (*S* = 0) results.

For the co-facial orientation in [**2˙˙**]^2+^ (*S* = 0), the high energy band is predicted to have significantly increased oscillator strength in comparison to the low energy band (*f* = 0.406 *vs. f* = 0.055, see Table 4), in accordance with the experimental results and the exciton coupling model. We have previously hypothesized that the NIR band splitting in [**3˙˙**]^2+^ is a result of exciton coupling of the radical salen units in an oblique geometry,17*a* and the data presented herein for the full series of dimers provides confirmation of this interpretation. Interestingly, the intensity ratio of the two predicted NIR bands for [**3˙˙**]^2+^ is opposite that observed experimentally and predicted by the Kasha model.10*b* Further analysis of the DFT computed geometry shows that the chromophores are not perfectly oblique, and thus there is likely an additional orientational component contributing to the NIR band intensity ratio.^45^ The increased inter-chromophore angle in [**4˙˙**]^2+^ relative to [**3˙˙**]^2+^, results in the low energy band exhibiting significantly greater intensity in comparison to the high energy band (Fig. 7C), and this is correctly predicted by the TD-DFT calculations (*f* = 0.395 *vs. f* = 0.092, see Table 4). Finally, for the parallel orientation in [**5˙˙**]^2+^, only the low energy band is predicted to exhibit significant intensity (Table 4), reflecting the limited capacity of the forbidden transition to gain intensity *via* symmetry perturbations in this chromophore geometry.^43^

## Summary

Investigation of the influence of inter-chromophore distance and relative geometry on photophysical properties in discrete dimers provides a mechanism to understand the absorption and emission properties in aggregated systems.^46^ In this context, a series of bimetallic Ni salen complexes have been prepared and studied (**2–5**), in which the relative geometry of the salen units has been systematically varied from cofacial to head-to-tail. All complexes are easily oxidized to the corresponding bis-ligand radical species, in which the ligand radicals are confined to the individual salen units. The xanthene spacer of **2** has previously been employed in photophysical studies of chromophore interactions,^30^ and the close Ni···Ni distance of 3.98 Å is within a range for the observation of strong interactions *via* direct wave-function overlap.^14^ Interestingly, we observe a weak spin–spin interaction of the ligand radical units in [**2˙˙**]^2+^*via* X- and Q-band EPR spectroscopy, and this is further corroborated by DFT calculations. EPR simulation of the oblique dimer [**3˙˙**]^2+^ as a triplet affords very small ZFS parameters, demonstrating that a non-interacting biradical description is appropriate for this derivative.^42^,^47^ Most interestingly, for the doubly oxidized species we observe significant shifts in the intense intervalence charge transfer (IVCT) band in the NIR, matching that predicted by the exciton model. This study shows, for the first time, the applicability of the exciton model to bis-ligand radical systems absorbing in the NIR, and we demonstrate the ability to tune the energy of absorption by nearly 400 nm in this low energy region. We plan to further study the interesting aggregation process and associated photophysical properties of the *para* dimer [**5˙˙**]^2+^, which under the current conditions is irreversible.

## Conflicts of interest

There are no conflicts to declare.

## Supplementary Material

Supplementary information

Crystal structure data
