# Supplementary material for: Exploiting exciton coupling of ligand radical intervalence charge transfer transitions to tune NIR absorption
Source: Chem Sci. 2017 Dec 19;9(6):1610–20. doi: 10.1039/c7sc04537a (PMC5887452; doi:10.1039/c7sc04537a)
Supplement: Supplementary file 1 [file SC-009-C7SC04537A-s001.pdf]

Electronic Supplementary Material (ESI) for Chemical Science.
This journal is © The Royal Society of Chemistry 2018

Exploiting Exciton Coupling of Ligand Radical Intervalence Charge Transfer Transitions to Tune NIR Absorption

Supporting Information

Ryan M. Clarke,† Tiffany Jeen,† Serena Rigo,† John R. Thompson,† Fabrice Thomas,§ and Tim Storr†

†Department of Chemistry, Simon Fraser University, Burnaby, British Columbia, V5A 1S6, Canada

§Départment de Chimie Moléculaire – Chimie Inorganique Redox (CIRE) – UMR CNRS 5250, Université Joseph Fourier, B.P. 53, 38041 Grenoble Cedex 9, France.

Table of Contents

Experimental…..……………………………………………………………………….S3-8

Experimental and Calculated Metrical Parameters……………………………………..S8

X-ray Data………………………………………………………………………………...S9

Differential Pulse Voltammetry of **2**, **4**, and **5**…………………………………..…..…S9

Supplementary EPR Figures……………………………………………………….…..S10-11

Full UV-vis-NIR data for **2**, **4**, and **5**…………………………………………………S12-13

DLS experiment on aggregated [**5**••]$^{2+}$…………………………………………………..S14

Solvent-dependence of [**5**••]$^{2+}$…………………………………………………….….S15-16

Triplet Spin Density Plots……………………………………………………………...S17

Kohn-Sham Molecular Orbitals Involved in the TD-DFT Predicted Transitions

(*S* = 1)……………………………………………………………………………..…S17-18

Kohn-Sham Molecular Orbitals Involved in the TD-DFT Predicted Transitions

(*S* = 0)………………………………………………………………………………S19

Computational Data…………………………………………………………………...S20-80

References……………………………………………………………………………..S80-81

Experimental Section

Materials and Methods. All chemicals used were of the highest grade available and were further purified whenever necessary. Literature methods were followed in order to prepare 5,5'-(2,7-di-*tert*-butyl-9,9-dimethyl-9*H*-xanthene-4,5-diyl)bis(3-(*tert*-butyl)-2-hydroxybenzaldehyde)[1] and (*E*)-2-(1((2-amino-2-methylpropyl)imino)ethyl)-4,6-di-*tert*-butylphenol.[2] The tris(2,4-dibromophenyl) aminium hexafluoroantimonate radical chemical oxidant $[N(C_6H_3Br_2)_3][SbF_6]$ ($E_{1/2}$ = 1.14 V vs. Fc^+/Fc, MeCN) was synthesized according to published protocols.[3] Electronic spectra were recorded on a Cary 5000 spectrophotometer with variable pathlength (1 and 10 mm, Hellma, Inc.). Constant temperatures were maintained by a dry ice/acetone bath. Cyclic voltammetry (CV) was performed on a PAR-263A potentiometer, equipped with a Ag wire reference electrode, a Pt disk working electrode, and a Pt counter electrode with $^nBu_4NClO_4$ (0.1 M) solution in CH_2Cl_2. Decamethylferrocene was used as an internal standard.[4] ^{1}H NMR spectra were recorded on a Bruker AV-400 instrument. Mass spectra (positive ion) were obtained on a Bruker Microflex LT MALDI-TOF MS instrument. Elemental analyses (C, H, N) were performed by Mr. Paul Mulyk at Simon Fraser University on a Carlo Erba EA1110 CHN elemental analyzer. Electron paramagnetic resonance (EPR) spectra were collected using a Bruker EMXplus spectrometer with a premiumX X-band microwave bridge and a dual mode resonator connected to an Oxford Instruments He flow cryostat. Samples for X-band EPR measurements were placed in 4 mm outer diameter tubes with sample volumes of ~250 µL. For the Q-Band measurements the same apparatus was used, with a 33.9 GHz microwave bridge and a qwt resonator. EPR spectra were simulated using the EasySpin package in MATLAB.[5]

X-ray Structure Determination. Single crystal X-ray crystallographic analysis of **2** was collected at 150 K on a D8 goniostat equipped with a Bruker PHOTON100 CMOS detector at Beamline 11.3.1 at the Advanced Light Source (Lawrence Berkeley National Laboratory) using synchrotron radiation tuned to λ = 0.7749 Å. For data collection frames were measured for a duration of 2 s at 0.5° intervals of ω. **4** and **5** was performed on a Bruker SMART diffractometer equipped with an APEX II CCD detector and IµSCuKα (λ = 1.54184 nm) microfocus sealed X-ray tube fitted with HELIOS multilayer optics. Crystals were mounted on MiTeGen dual-thickness MicroMounts using parabar oil. The data was collected at room temperature (approximated to 296 K) (**5**) and 150(2) K (via an Oxford Cryosystems cold-stream) (**4**). Data was collected in a series of φ and ω scans with 1.00° image widths and 60 second exposures. The crystal-to-detector distance was 40 mm. Data was processed using the Bruker APEX II software suite. Structures were solved using intrinsic phasing method[6] and refined with ShelXl within ShelXle.[7] The structures of **2** and **5** contained heavily disordered solvent that could not be successfully modelled

thus, the PLATON/SQUEEZE[8] program was used to generate a 'solvent-free' HKLF5 format data set. The equivalent of 4 molecules of hexane and 4 molecules of CH_2Cl_2 (**2**) and 28 molecules of hexane (**5**) was removed from the unit cell. All non-hydrogen atoms were refined anisotropically. All C-H hydrogen atoms were placed in geometrically calculated positions without further refinement. All crystal structure plots were produced using ORTEP-3[9] and rendered using POV-Ray.[10] A summary of the crystal data for the structure determination is given in Table S2.

Oxidation Protocol. Under an inert atmosphere at 195 K, 500 μL of a CH_2Cl_2 solution of the metal complex (4.6 mM) was added to 3.0 mL of dry CH_2Cl_2. Monitored by UV-vis-NIR spectroscopy, a saturated solution of $[N(C_6H_3Br_2)_3][SbF_6]$ in CH_2Cl_2 was added in 60 μL aliquots resulting in clean conversion to the respective one- and two-electron oxidized species.

Calculations. Geometry optimizations were performed using the Gaussian 09 program (Revision D.01),[11] the B3LYP functional,[12-13] and 6-31G(d) basis set on all atoms. This combination of functional and basis set has been used previously for structurally similar salen complexes,[2, 14-16] providing a good match to experimental metrical parameters. Frequency calculations at the same level of theory confirmed that the optimized structures were located at a minimum on the potential energy surface. Single-point calculations for energetic analysis were performed using the B3LYP functional, and the TZVP basis set of Ahlrichs on all atoms.[17-18] The intensities of the 10 lowest-energy electronic transitions were calculated by time-dependent density functional theory (TD-DFT)[19-20] at the B3LYP/TZVP level with a polarized continuum model (PCM) for CH_2Cl_2 (dielectric ε = 8.94).[21-24]

Synthetic Schemes

Scheme S1. Synthesis of **2**[a]

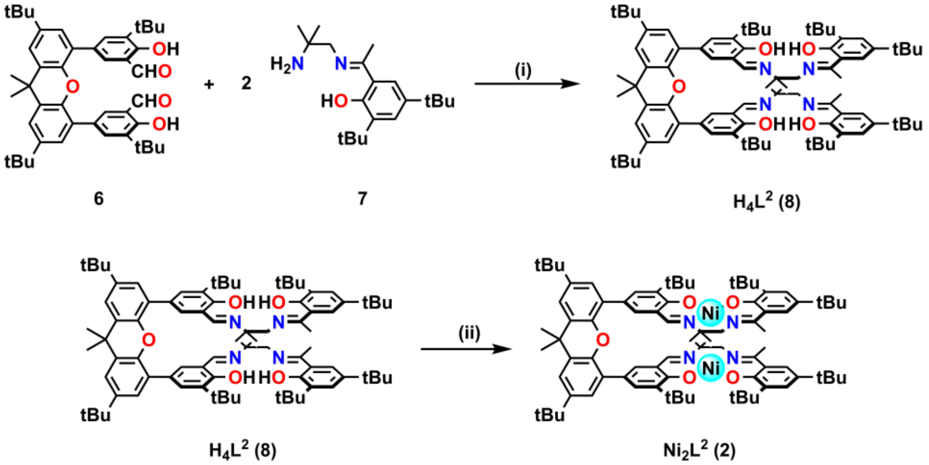

[a](i) CH_2ClCH_2Cl, 52%; (ii) $Ni(OAc)_2$•$4H_2O$, DMF, 82%.

Scheme S2. Synthesis of **4**[a]

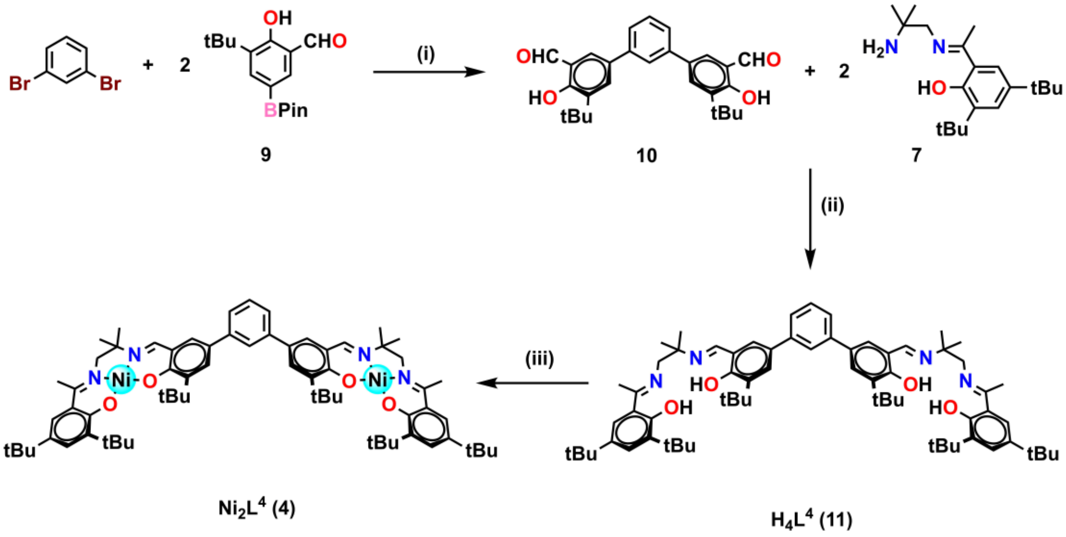

[a](i) $Pd(PPh_3)_4$, K_2CO_3, 5:1 DME/H_2O, 53%; (ii) THF, 75%; (iii) $Ni(OAc)_2$•$4H_2O$, DMF, 91%.

Scheme S3. Synthesis of **5**[a]

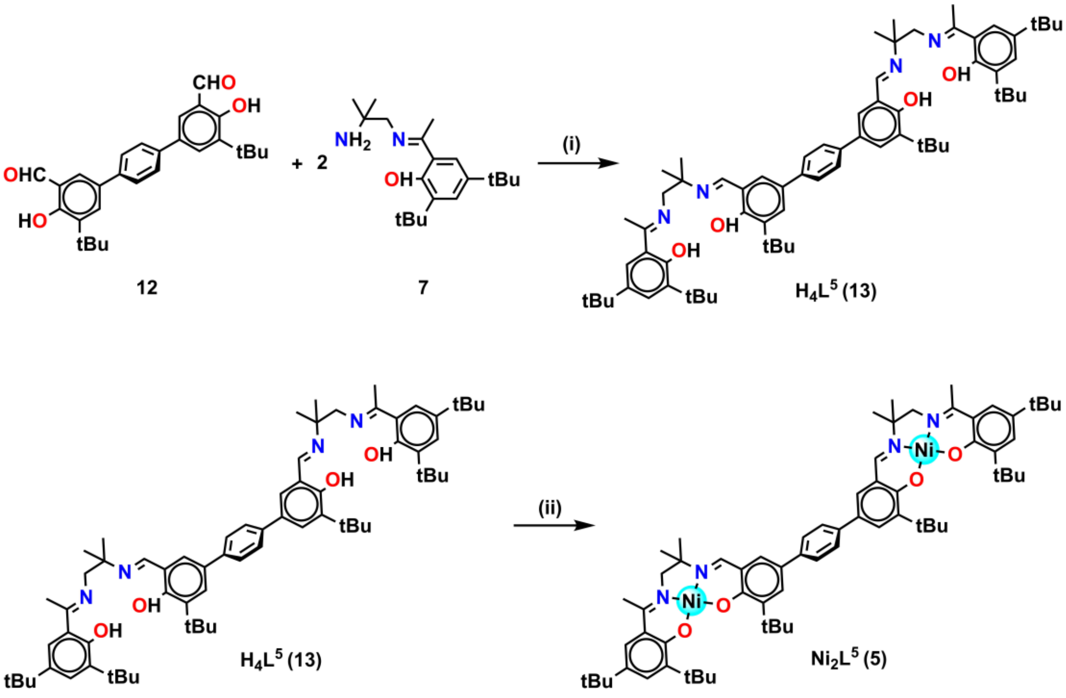

[a](i) THF, 87%; (ii) Ni(OAc)2•4H2O, DMF, 88%.

Synthetic Details

*H_4L^2 (**8**).* A 10 mL degassed dichloroethane solution of **6** (0.49 g, 0.73 mmol) was added to a refluxing 10 mL degassed dichloroethane solution of **7** (0.47 g, 1.5 mmol). The yellow mixture was refluxed for 24 hours, after which the mixture was cooled and the solvent was removed *in vacuo* until only 5 mL remained. Cold methanol was added to the mixture (20 mL) and a bright yellow precipitate formed which was collected *via* filtration and washed with methanol and pentane (3x10 mL each). Yield: 0.48 g, 52%. MALDI-MS *m/z*: 1275.51 (100%). ^{1}H NMR (400 MHz, $CDCl_3$): δ = 8.12 (s, 1H), 7.46 (d, *J* = 2.3 Hz, 2H), 7.41 (d, *J* = 2.4 Hz, 2H), 7.35 (m, 4H), 7.32 (d, *J* = 2.2 Hz, 2H), 7.17 (d, *J* = 2.4 Hz, 2H), 3.61 (s, 4H), 2.31 (s, 6H), 1.78 (s, 6H), 1.39 (s, 9H), 1.38 (s, 12H), 1.37 (s, 9H), 1.29 (s, 9H), 1.17 (s, 9H).

*Ni_2L^2 (**2**).* To a solution of **8** (50 mg, 0.039 mmol) in 3 mL dry dimethylformamide (DMF) was added a 3 mL dry DMF $Ni(OAc)_2$•$4H_2O$ solution (40 mg, 0.16 mmol). The mixture turned deep brown upon addition of the metal salt, and was allowed to stir at reflux for 16 hours. The mixture was then cooled to room temperature and placed in an ice bath. Cold water was added (10 mL) to precipitate a brown solid which was collected *via* suction filtration. The crude material was recrystallized in CH_2Cl_2/hexanes (1:1) to obtain light brown crystals. Yield: 45 mg, 82%. MALDI-MS *m/z*: 1389.51 (100%). ^{1}H NMR (400 MHz CD_2Cl_2): δ = 7.52 (s, 2H), 7.50 (s, 2H), 7.35 (d, *J* = 2.4 Hz, 2H), 7.27 (d, *J* = 2.5 Hz, 2H), 7.24 (d, *J* = 2.4 Hz, 2H), 7.02 (d, *J* = 2.4 Hz, 2H), 6.89 (s, 1H), 1.71 (s, 6H), 1.61 (s, 8H), 1.44 (s, 18H), 1.42 (s, 18H), 1.40 (s, 12H), 1.35 (s, 18H), 1.24 (s, 18H) Anal. Cald. (%) for $C_{85}H_{114}N_4O_5Ni_2$: C 70.82, H 8.40, N, 3.59; Found (%): C 70.77, H 8.56, N 3.71.

*5-5”-di-*tert-*butyl-4-4”-dihydroxy-[1,1’:3’,1”-terphenyl]-3,3”-dicarbaldehyde (**10**)*. A 2-neck flask was charged with 2-hydroxy-3-tertbutyl-5-Bpin-benzaldehyde (500 mg, 1.63 mmol), K_2CO_3 (215 mg, 1.56 mmol), and $Pd(PPh_3)_4$ (55 mg, 0.048 mmol) and subjected to three evacuation/refill cycles. 10 mL of degassed DME/H_2O (5:1) was added to the solids. 1,3-dibromobenzene (93 μL, 0.78 mmol) was added and the mixture was heated at reflux for 24 hours, after which the mixture was allowed to cool to room temperature. The mixture was extracted with CH_2Cl_2 (3x20 mL). The solvent was removed *in vacuo* and the crude material as purified *via* flash column chromatography (2.5% EtOAc in hexanes) to afford the title compound as a yellow solid. X-ray quality crystals were grown from a concentrated CH_2Cl_2 solution layered with hexanes. Yield: 78.3 mg, 53%. MALDI-MS *m/z*: 430.92 (100%). ^{1}H NMR (400 MHz, $CDCl_3$): δ = 11.83 (s, 2H), 9.99 (s, 2H), 7.80 (d, *J* = 2.3 Hz, 2H), 7.70-7.66 (m, 1H), 7.65 (d, *J* = 2.3 Hz, 2H), 7.55-7.53 (m, 3H), 1.49 (s, 18H). Anal. Cald. (%) for $C_{28}H_{30}O_4$: C 78.11, H 7.02; Found (%): C 78.31, H 7.18.

*H_4L^4 (**11**).* To a stirring solution of **10** (140 mg, 0.33 mmol) in THF (40 mL) was added **7** (217 mg, 0.68 mmol). The mixture was heated at reflux for 24 hours, after which it was allowed to cool to room temperature. The solvent was removed *in vacuo* and the crude material was recrystallized in hot MeOH. Yield: 250 mg, 75%. MALDI-MS *m/z*: 1031.95 (100%). ^1H NMR (500 MHz, $CDCl_3$): δ = 8.55 (s, 2H), 7.66 (d, *J* = 2.1 Hz, 1H), 7.58 (d, *J* = 2.3 Hz, 2H), 7.45 (d, *J* = 1.3 Hz, 3H), 7.42 (d, *J* = 2.2 Hz, 2H), 7.38 (d, *J* = 2.4 Hz, 2H), 7.35 (d, *J* = 2.4 Hz, 2H), 3.71 (s, 4H), 2.36 (s, 6H), 1.52 (s, 12H), 1.48 (s, 18H), 1.38 (s, 18H), 1.29 (s, 18H). Anal. Cald. (%) for $C_{68}H_{94}N_4O_4$: C 79.18, H 9.19, N 5.43; found (%): C 79.31, H 9.28, N 5.20.

*Ni_2L^4 (**4**).* To a stirring solution of **11** (50 mg, 0.05 mmol) in DMF (5 mL) was added $Ni(OAc)_2$•$4H_2O$ (48 mg, 0.2 mmol) in DMF (5 mL). The mixture was heated at reflux for 5 hours, after which it was cooled to room temperature. Cold water was added dropwise to precipitate a brown solid which was collected *via* suction filtration. The solid material was washed with H_2O (10 mL) and recrystallized in CH_2Cl_2/MeOH at room temperature to yield a brown microcrystalline material. X-ray quality crystals were grown from a concentrated acetonitrile solution. Yield: 51 mg, 91%. MALDI-MS *m/z*: 1143.19 (100%). ^1H NMR (500 MHz, $CDCl_3$): δ = 7.60 (s, 1H), 7.53 (d, *J* = 2.5 Hz, 2H), 7.45 (s, 2H), 7.34 (m, 3H), 7.30, (d, *J* = 2.5 Hz, 2H), 7.21 (d, *J* = 2.4 Hz, 2H), 7.19 (d, *J* = 2.5 Hz, 2H), 3.23 (s, 4H), 2.34 (s, 6H), 1.46 (s, 12H), 1.45 (s, 18H), 1.43 (s, 18H), 1.29 (s, 18H). Anal. Cald. (%) for $C_{68}H_{90}N_4O_4Ni_2$: C 71.34, H 7.92, N 4.89; found (%): C 71.60, H 8.11, N 4.58.

*H_4L^5 (**13**).* To a stirring solution of **12** (100 mg, 0.23 mmol) in THF (40 mL) was added **7** (150 mg, 0.49 mmol). The mixture was heated at reflux for 24 hours, after which it was allowed to cool to room temperature. The solvent was removed *in vacuo* and the crude material was recrystallized in hot MeOH. Yield: 210 mg, 87%. MALDI-MS *m/z*: 1031.31 (100%). ^1H NMR (500 MHz, $CDCl_3$): δ = 8.56 (s, 2H), 7.58 (m, 6H), 7.43 (d, *J* = 2.3 Hz, 2H), 7.39-7.35 (m, 4H), 3.72 (s, 4H), 2.37 (s, 6H), 1.53 (s, 12H), 1.49 (s, 18H), 1.42 (s, 18H), 1.29 (s, 18H). Anal. Cald. (%) for $C_{68}H_{94}N_4O_4$: C 79.18, H 9.19, N 5.20; found (%): C 79.40, H 9.33, N 4.96.

*Ni_2L^5 (**5**).* To a stirring solution of **13** (50 mg, 0.05 mmol) in DMF (5 mL) was added $Ni(OAc)_2$•$4H_2O$ (48 mg, 0.2 mmol) in DMF (5 mL). The mixture was heated at reflux for 5 hours, after which it was cooled to room temperature. Cold water was added dropwise to precipitate a brown solid which was collected *via* suction filtration. The solid material was washed with H_2O (10 mL) and recrystallized in hot pyridine. Yield: 50 mg, 88%. MALDI-MS *m/z*: 1144.31 (100%). ^1H NMR (500 MHz, $CDCl_3$): δ = 7.68 (s, 4H), 7.60 (d, *J* = 2.5 Hz, 2H), 7.37 (s, 2H), 7.31 (d, *J* = 2.3 Hz, 2H), 7.29 (d, *J* = 2.4 Hz, 2H), 7.09 (d, *J* = 2.4

Hz, 2H), 3.50 (s, 4H), 2.07 (s, 6H), 1.46 (s, 18H), 1.45 (s, 18H), 1.39 (s, 12H), 1.26 (s, 18H). Anal. Cald. (%) for $C_{68}H_{90}N_4O_4Ni_2$: C 71.34, H 7.92, N 4.89; found (%): C 71.55, H 8.09, N 4.71.

Table S1. Experimental and calculated (in parentheses)[a] coordination sphere metrical parameters for the complexes [Å].

Complex	Ni(1)-O(1)	Ni(1)-O(2)	Ni(1)-N(1)	Ni(1)-N(2)	Ni(2)-O(3)	Ni(2)-O(4)	Ni(2)-N(3)	Ni(2)-N(4)
**2**	1.828 (1.844)	1.871 (1.857)	1.862 (1.858)	1.841 (1.850)	1.854 (1.853)	1.836 (1.846)	1.842 (1.851)	1.862 (1.857)
[**2**••]$^{2+}$ (broken symmetry)	(1.819)	(1.845)	(1.851)	(1.845)	(1.843)	(1.819)	(1.844)	(1.851)
[**2**••]$^{2+}$ (triplet)	(1.824)	(1.840)	(1.850)	(1.845)	(1.846)	(1.821)	(1.846)	(1.850)
**4**	1.832 (1.836)	1.854 (1.857)	1.858 (1.857)	1.845 (1.852)	1.859 (1.851)	1.833 (1.830)	1.851 (1.848)	1.855 (1.852)
[**4**••]$^{2+}$ (broken symmetry)	(1.823)	(1.833)	(1.857)	(1.840)	(1.827)	(1.814)	(1.837)	(1.847)
[**4**••]$^{2+}$ (triplet)	(1.823)	(1.833)	(1.857)	(1.840)	(1.828)	(1.814)	(1.837)	(1.847)
**5**	1.827 (1.830)	1.864 (1.852)	1.861 (1.852)	1.854 (1.848)	1.860 (1.852)	1.824 (1.830)	1.847 (1.848)	1.856 (1.852)
[**5**••]$^{2+}$ (broken symmetry)	(1.814)	(1.829)	(1.848)	(1.838)	(1.829)	(1.814)	(1.838)	(1.848)
[**5**••]$^{2+}$ (triplet)	(1.814)	(1.829)	(1.848)	(1.838)	(1.829)	(1.814)	(1.838)	(1.848)

[a]See the Experimental Section for calculation details.

Table S2. Selected crystallographic data for **2, 4** and **5**.

	**2**	**4**	**5**
Formula	$C_{92}H_{130}N_4Ni_2O_5$	$C_{72}H_{96}N_6Ni_2O_4$	$C_{89}H_{139}N_4Ni_2O_4$
Formula weight	1560.31	1226.93	1446.45
Space group	P 21/n	P -1	P b c a
a (Å)	15.5829(11)	11.4567(7)	29.9305(5)
b (Å)	39.582(3)	13.1358(10)	12.1234(2)
c (Å)	16.2027(11)	25.5529(16)	47.9429(8)
α (deg)	90	82.482(5)	90
β (deg)	110.448(2)	89.480(4)	90
γ (deg)	90	67.190(5)	90
V ($Å^3$)	9364.0(11)	3510.7(4)	17396.5(5)
Z	4	2	8
T (K)	150	150	296
ρ_{calcd} ($g\ cm^{-3}$)	1.107	1.161	1.105
λ (Å)	0.77490	1.54178	1.54178
μ (cm^{-1})	0.6370	1.043	0.898
R indices[a] with I > 2.0σ(I) (data)	0.0439	0.0599	0.0555
wR_2	0.1126	0.1685	0.1250
R_1	0.0626	0.07934	0.0893
Goodness-of-fit on F^2	1.033	1.044	1.080

[a]Goodness-of-fit on *F*

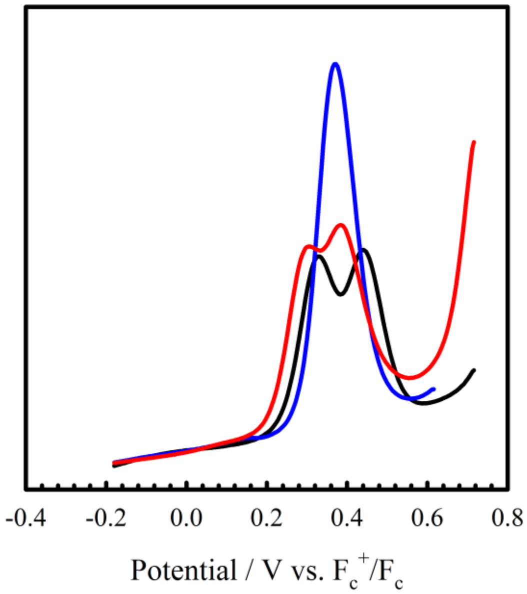

Figure S1. Differential pulse voltammetry (DPV) scans of **3** (black line), **4** (blue line), and **5** (red line).

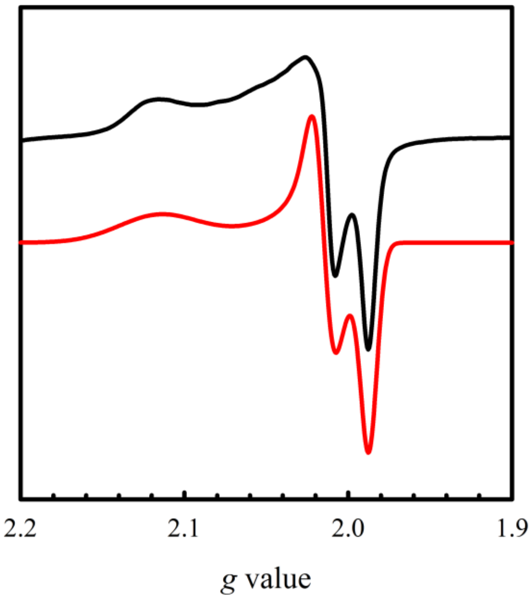

Figure S2. X-band EPR spectrum of [**2**$^{\bullet}$]$^{+}$ recorded in frozen CH_2Cl_2 at 1.2 mM. Conditions: frequency = 9.64 GHz; power = 2 mW; modulation frequency = 100 kHz; modulation amplitude = 0.4 mT; T = 8 K. The red line represents simulation to the experimental data using the parameters: g_1 = 2.118, g_2 = 2.015, and g_3 = 1.987.

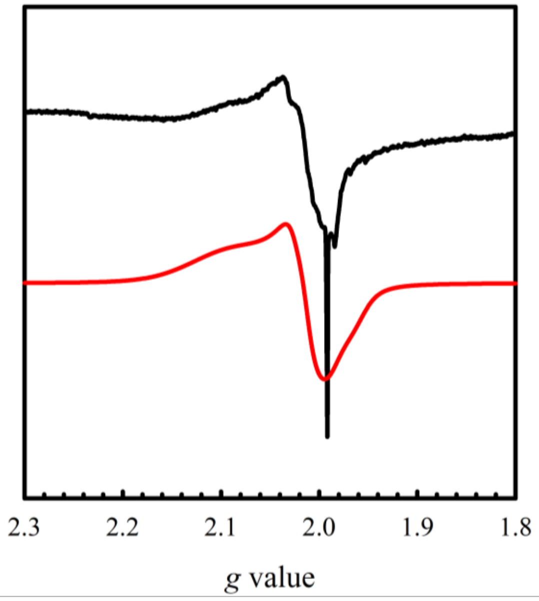

Figure S3. Q-band EPR spectrum of [**2**$^{\bullet\bullet}$]$^{2+}$ recorded in frozen CH_2Cl_2 at 0.8 mM. Conditions: frequency = 33.92 GHz; power = 1.4 mW; modulation frequency = kHz;

modulation amplitude = 0.5 mT; T = 6 K. The red line represents simulation to the data using the parameters given in the main text. The sharp peak originates from the monoradical [**2**$^{\bullet}$]$^{+}$.

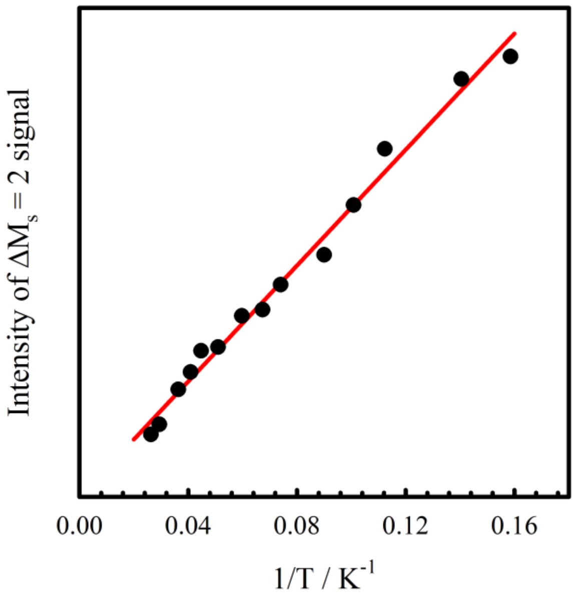

Figure S4. Curie-plot of the half-field signal for [**2**$^{\bullet\bullet}$]$^{2+}$.

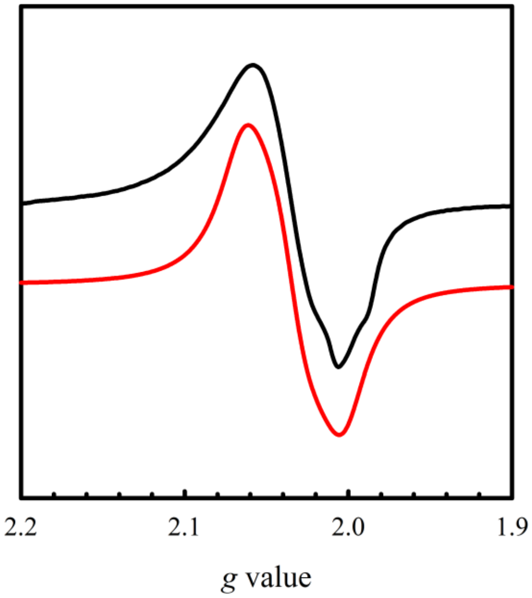

Figure S5. X-band EPR spectrum of [**4**$^{\bullet}$]$^{+}$ recorded in frozen CH_2Cl_2 at 1.4 mM. Conditions: frequency = 9.64 GHz; power = 2 mW; modulation frequency = 100 kHz;

modulation amplitude = 0.2 mT; T = 16 K. The red line represents simulation to the experimental data using the parameters: g_1 = 2.062, g_2 = 2.034, g_3 = 2.002.

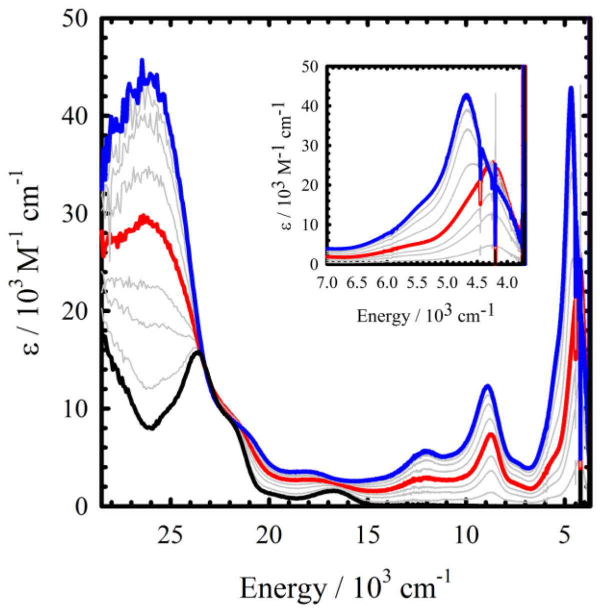

Figure S6. Oxidation titration data for **2** (black line) to [**2**$^{\bullet\bullet}$]$^{2+}$ (blue line). Intermediate gray lines were measured during the oxidation titration with [N($C_6H_3Br_2$)$_3$]$^{+\bullet}$[SbF_6]$^-$. The red line represents addition of 1 equivalent of oxidant to generate [**2**$^{\bullet}$]$^{+}$. Conditions: CH_2Cl_2, 0.33 mM complex, 198 K.

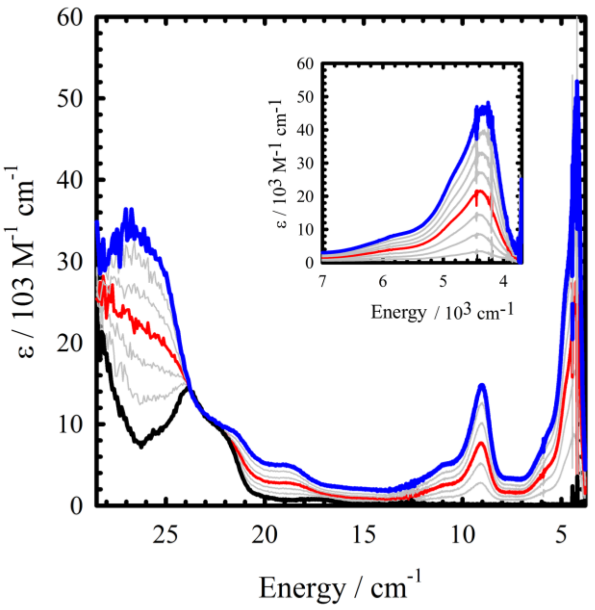

Figure S7. Oxidation titration data for **4** (black line) to $[\mathbf{4}^{\bullet\bullet}]^{2+}$ (blue line). Intermediate gray lines were measured during the oxidation titration with $[N(C_6H_3Br_2)_3]^{+\bullet}[SbF_6]^-$. The red line represents addition of 1 equivalent of oxidant. Conditions: CH_2Cl_2, 0.33 mM complex, 198 K.

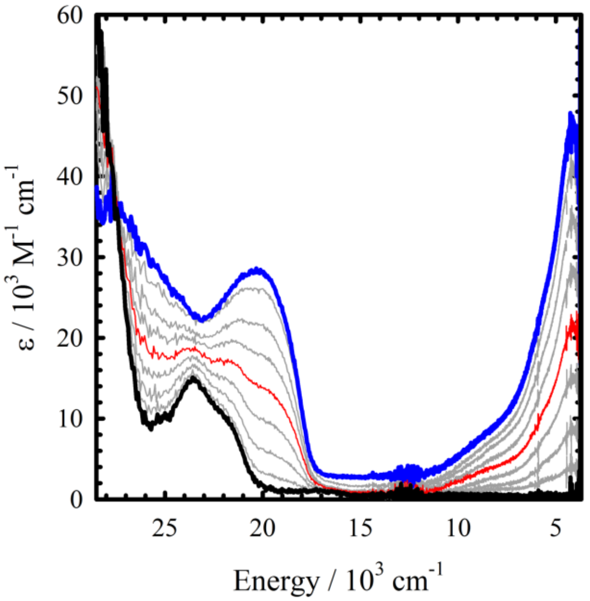

Figure S8. Oxidation titration data for **5** (black line) to $[\mathbf{5}^{\bullet\bullet}]^{2+}$ (blue line). Intermediate gray lines were measured during the oxidation titration with $[N(C_6H_3Br_2)_3]^{+\bullet}[SbF_6]^-$. The red line represents addition of 1 equivalent of oxidant to generate $[\mathbf{5}^{\bullet}]^{+}$. Conditions: CH_2Cl_2, 0.08 mM complex, 298 K.

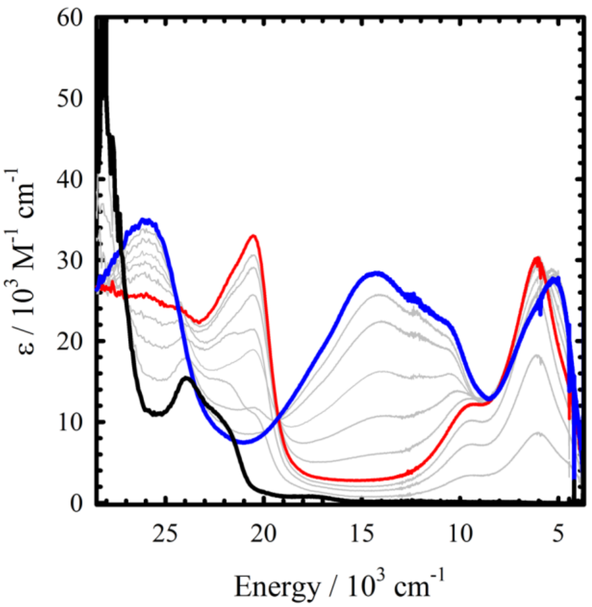

Figure S9. Oxidation titration data for **5** (black line) at low temperature (198 K). The red line represents addition of 1 eq. chemical oxidant, the blue line addition of 2 eq. chemical oxidant. Grey lines are measured during sequential addition of $[N(C_6H_3Br_2)_3]^{\bullet+}[SbF_6]^-$ during the titration.

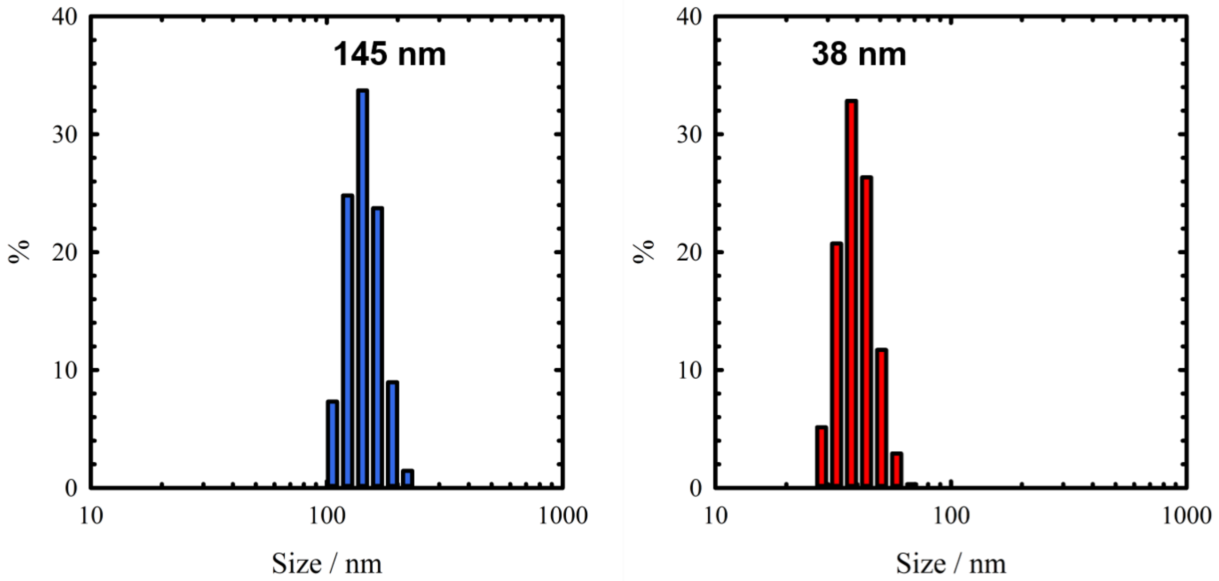

Figure S10. DLS measurement of aggregates formed *via* low temperature oxidation of **5** (left) and room temperature oxidation (right). Conditions: 0.1 mM, CH_2Cl_2.

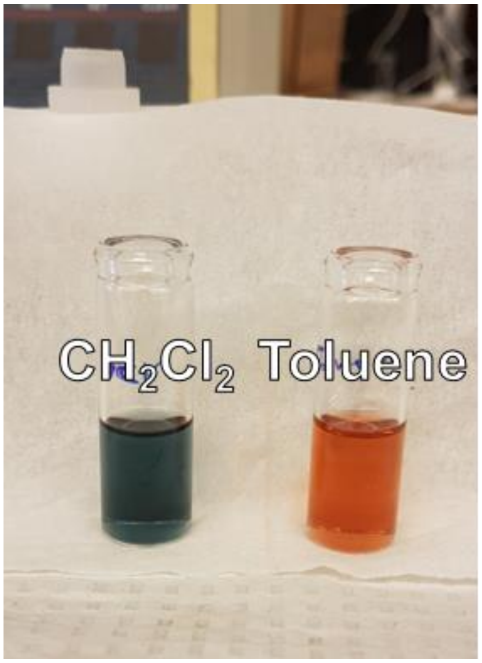

Figure S11. 1 mM solutions of [**5**••]$^{2+}$ in CH_2Cl_2 (left) and toluene (right).

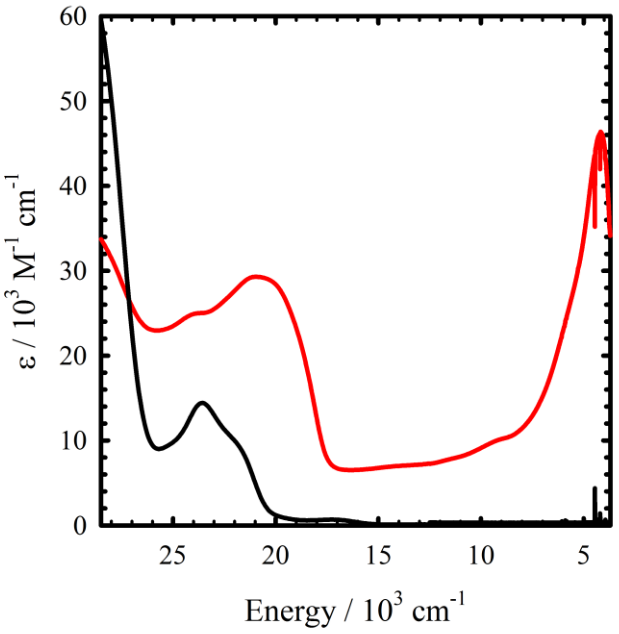

Figure S12. UV-vis-NIR spectra of **5** (black) and [**5**••]$^{2+}$ (red) recorded in toluene at 0.33 mM and 298 K.

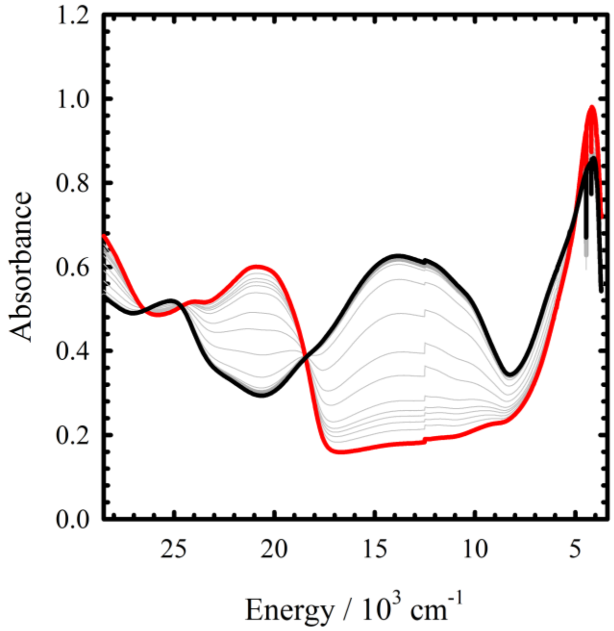

Figure S13. Dilution of a DCM solution (1 mM) of [**5**$^{\bullet\bullet}$]$^{2+}$ into toluene (0.2 mM final concentration) causes disaggregation of [**5**$^{\bullet\bullet}$]$^{2+}$. The black spectrum is recorded at time 0, grey lines are recorded every 2.5 minutes until the red spectrum is reached (~25 minutes). Isosbestic points at 5,000, 18,500, 24,500, and 26,500 cm^{-1} indicate clean conversion from one species to another.

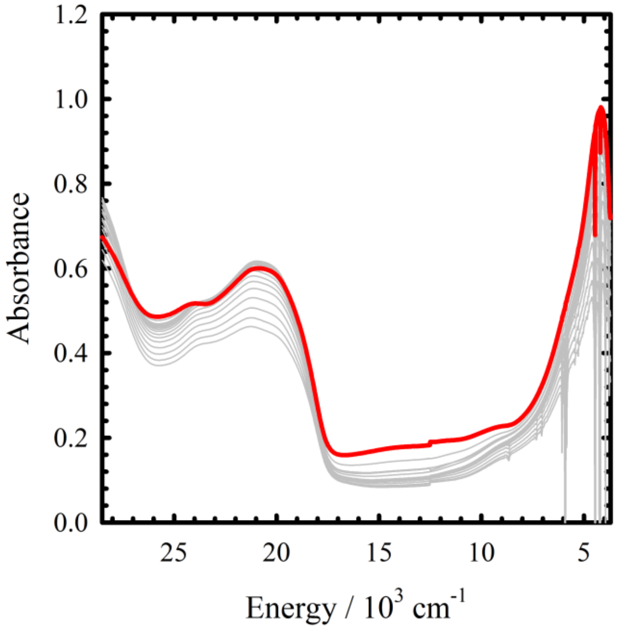

Figure S14. Decay of the NIR band for [**5**$^{\bullet\bullet}$]$^{2+}$ in toluene. Spectra recorded every 2.5 minutes.

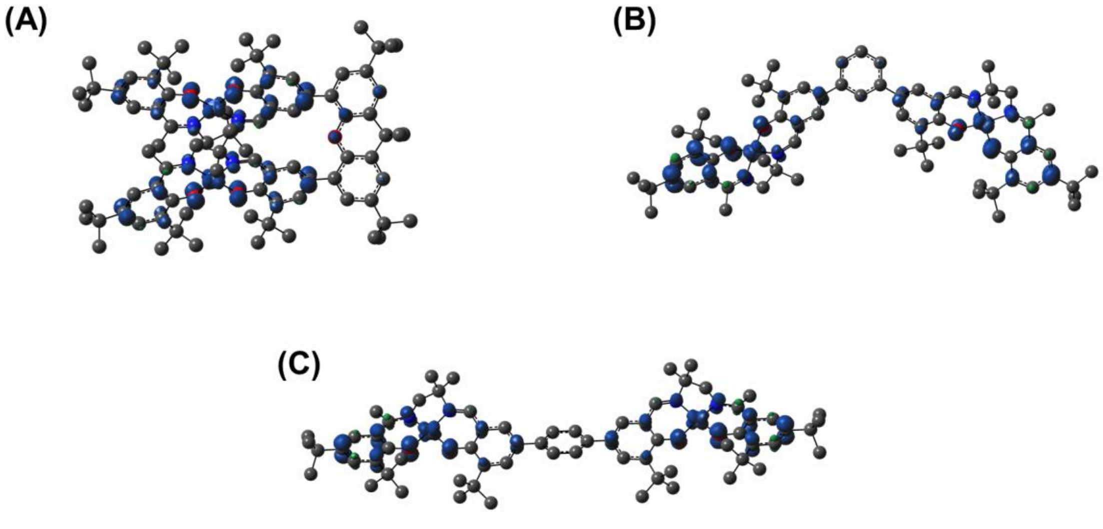

Figure S15. Spin density plots for the triplet (S = 1) solutions of (A) $[\mathbf{2}^{\bullet\bullet}]^{2+}$; (B) $[\mathbf{4}^{\bullet\bullet}]^{2+}$; and (C) $[\mathbf{5}^{\bullet\bullet}]^{2+}$.

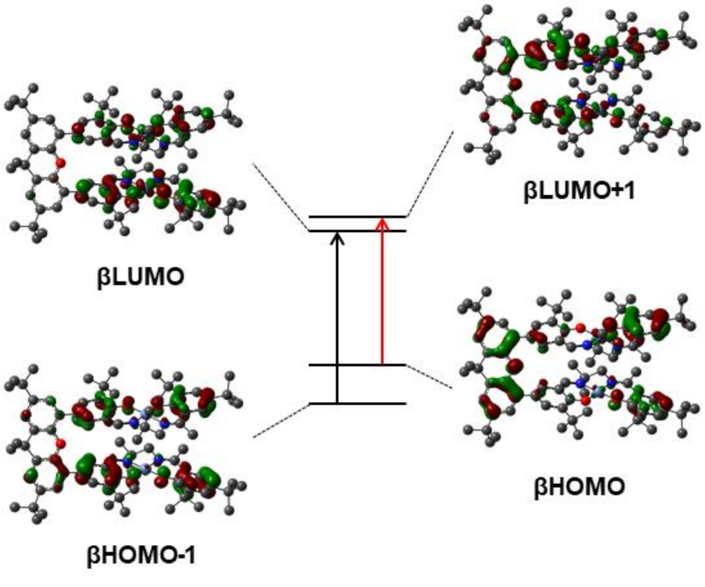

Figure S16. Kohn-Sham molecular orbitals of $[\mathbf{2}^{\bullet\bullet}]^{2+}$ (S = 1) associated with the calculated NIR transitions at 4385 cm^{-1} (βHOMO → βLUMO+1; red arrow; oscillator strength, f = 0.0704) and 5405 cm^{-1} (βHOMO-1 → βLUMO; black arrow; oscillator strength, f = 0.3855).

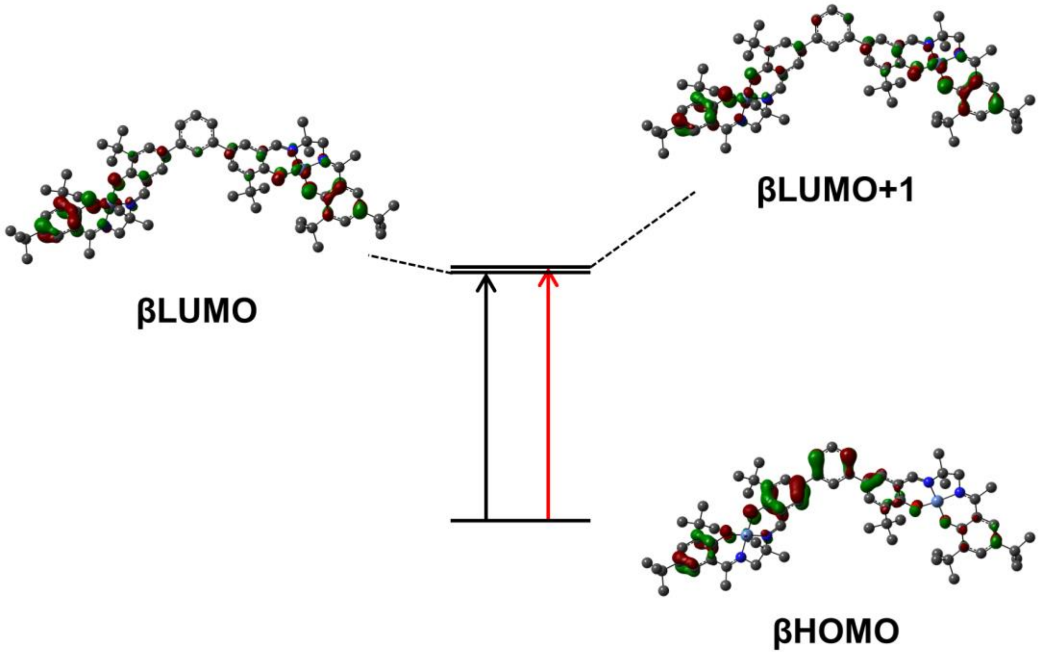

Figure S17. Kohn-Sham molecular orbitals of $[\mathbf{4}^{\bullet\bullet}]^{2+}$ (S = 1) associated with the calculated NIR transitions at 4935 cm^{-1} (βHOMO $\rightarrow$ βLUMO; black arrow; oscillator strength, f = 0.3971) and 5530 cm^{-1} (βHOMO $\rightarrow$ βLUMO+1; red arrow; oscillator strength, f = 0.0931).

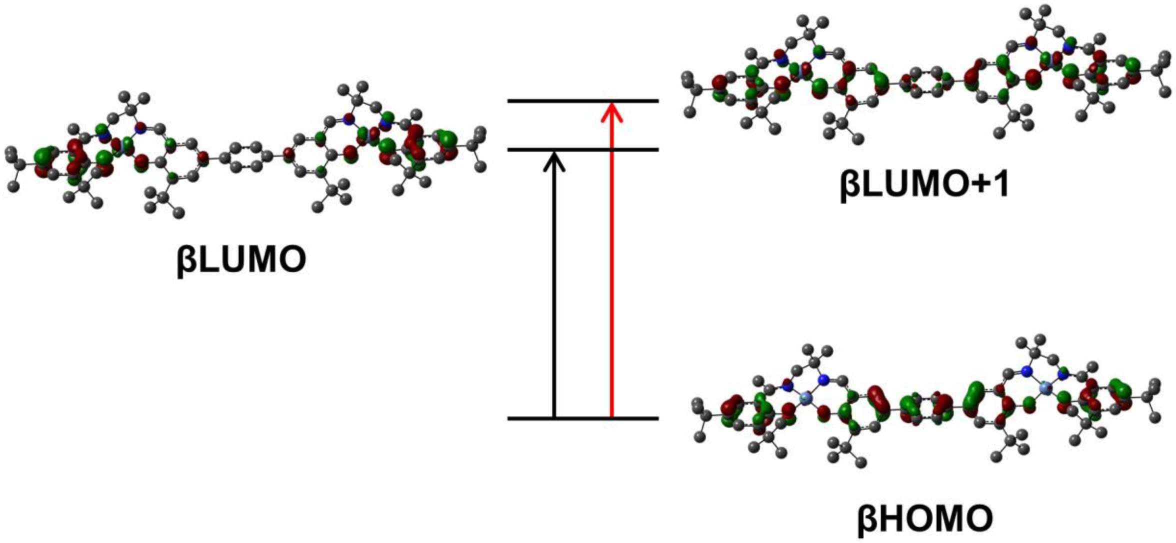

Figure S18. Kohn-Sham molecular orbitals of $[\mathbf{5}^{\bullet\bullet}]^{2+}$ (S = 1) associated with the calculated NIR transitions at 4300 cm^{-1} (βHOMO $\rightarrow$ βLUMO; black arrow; oscillator strength, f = 0.7111) and 5605 cm^{-1} (βHOMO $\rightarrow$ βLUMO+1; red arrow; oscillator strength, f = 0.002).

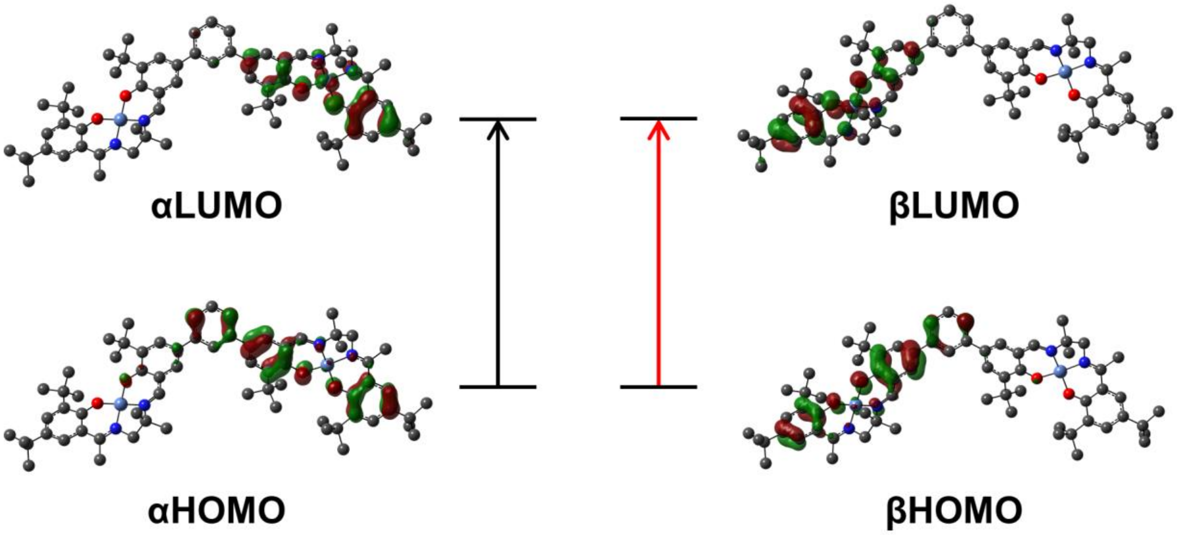

Figure S19. Kohn-Sham molecular orbitals for the broken symmetry (*S* = 0) solution of [**4**$^{\bullet\bullet}$]$^{2+}$ associated with the calculated NIR transitions at 5570 and 4935 cm^{-1} (black and red arrows; α/βHOMO → α/βLUMO). Predicted transitions are a result of symmetric and asymmetric combinations of the orbitals shown.

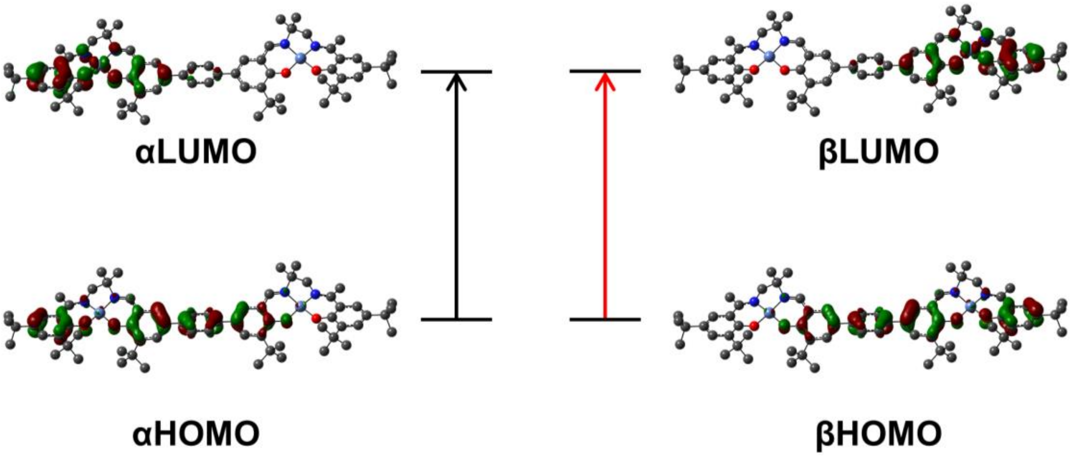

Figure S20. Kohn-Sham molecular orbitals for the broken symmetry (*S* = 0) solution of [**5**$^{\bullet\bullet}$]$^{2+}$ associated with the calculated NIR transitions at 5495 and 4390 cm^{-1} (black and red arrows; α/βHOMO → α/βLUMO). Predicted transitions are a result of symmetric and asymmetric combinations of the orbitals shown.

Computational Data

A) Optimized XYZ Coordinates (Å) for **2**

Ni	-1.640000	-1.984063	0.938004
O	-2.815756	-3.003449	-0.055343
N	-3.017335	-0.939549	1.615612
C	-4.093182	-3.201496	0.1548
Ni	-1.480054	2.118618	-1.050115
O	-0.276439	-3.015717	0.22307
N	-0.464005	-0.896115	1.865852
C	-4.772095	-4.278688	-0.531194
N	-0.322815	1.02547	-1.993339
O	-0.092311	3.128086	-0.341099
C	-6.099433	-4.521187	-0.21734
H	-6.593885	-5.348103	-0.712061
N	-2.871223	1.078128	-1.709397
O	-2.628481	3.168924	-0.060158
C	-6.857894	-3.773273	0.714365
C	-6.228867	-2.687564	1.284779
H	-6.769264	-2.081292	1.999093
O	5.519914	-0.078504	0.034391
C	-4.875919	-2.357332	1.007259
C	-4.038343	-5.158369	-1.563192
C	-2.903204	-5.936518	-0.860814
H	-2.193038	-5.255218	-0.394239
H	-2.364691	-6.560911	-1.584716
H	-3.313909	-6.596771	-0.087063
C	-3.466818	-4.274558	-2.698443
H	-2.779664	-3.524562	-2.305336
H	-4.278767	-3.764182	-3.232719
H	-2.926689	-4.894492	-3.424696
C	1.019328	-2.845067	0.25298
C	-8.322501	-5.619004	1.608977
H	-7.741437	-5.673938	2.536635
H	-7.897443	-6.344181	0.90696
H	-9.350079	-5.933262	1.831063
C	-8.962498	-3.247364	2.065266
H	-8.420665	-3.248792	3.018148
H	-9.987169	-3.578947	2.268104
H	-9.016663	-2.213848	1.703313
C	7.502698	-5.625412	2.510819
H	7.79583	-4.923209	3.299628
H	7.928942	-6.606969	2.752479

H	6.412097	-5.719707	2.536925
C	7.587126	-6.170279	0.048632
H	8.015214	-7.155856	0.270167
H	7.939422	-5.861334	-0.942209
H	6.499402	-6.285426	-0.004828
C	8.0055	-5.144645	1.128745
C	-4.311241	-1.142956	1.567553
C	-5.253689	-0.059107	2.068919
H	-5.285147	-0.026571	3.165552
H	-6.267642	-0.213592	1.70695
H	-4.933973	0.92439	1.715097
C	-2.487065	0.30709	2.185518
H	-3.157787	0.742555	2.928694
H	-2.350724	1.039054	1.378393
C	-1.140149	-0.003271	2.845642
C	-0.403451	1.311465	3.128419
H	-1.057127	1.971047	3.708881
H	-0.143214	1.831267	2.202272
H	0.503852	1.160617	3.72242
C	-9.157655	-4.142925	-0.258766
H	-10.190311	-4.44705	-0.046799
H	-8.765938	-4.816408	-1.028401
H	-9.180515	-3.131482	-0.680804
C	9.544488	-5.095181	1.169786
H	9.910516	-4.406449	1.939861
H	9.968708	-4.789343	0.206446
H	9.940153	-6.090156	1.403107
C	8.475748	-0.168126	0.086175
C	9.383985	-0.457931	-1.139356
H	10.061641	0.379463	-1.33564
H	8.779893	-0.6236	-2.037285
H	9.997338	-1.349404	-0.973143
C	9.359982	0.067401	1.340642
H	8.738457	0.269662	2.218954
H	10.031146	0.920335	1.195977
H	9.979372	-0.809171	1.557091
C	7.646935	1.08816	-0.185781
C	6.246199	1.076454	-0.179897
C	5.511448	2.26535	-0.3795
C	6.232177	3.44481	-0.631258
H	5.655081	4.34068	-0.824402
C	7.626466	3.498279	-0.667598
C	8.302275	2.298976	-0.430832

H	9.387594	2.296256	-0.439757
C	9.341201	5.108588	0.240626
H	8.759533	5.260006	1.157138
H	9.915219	6.023181	0.046736
H	10.057084	4.301625	0.430031
C	8.413877	4.789575	-0.956048
C	7.489839	6.00162	-1.179674
H	6.868974	6.209247	-0.300662
H	6.827085	5.856716	-2.040261
H	8.093772	6.895068	-1.375059
C	9.27085	4.598171	-2.229956
H	9.84462	5.50796	-2.446298
H	8.637648	4.380968	-3.097731
H	9.9834	3.77324	-2.122764
C	1.196841	2.907984	-0.343337
C	2.100989	3.813268	0.324063
C	3.450162	3.495371	0.318965
H	4.133923	4.152928	0.841052
C	4.027746	2.370907	-0.328327
C	3.161956	1.521743	-0.989173
H	3.54238	0.65774	-1.525536
C	1.771094	1.778214	-1.012037
C	1.604206	5.121285	0.971117
C	0.977639	6.011331	-0.130253
H	0.586802	6.939013	0.306029
H	0.159488	5.498589	-0.637898
H	1.733125	6.282393	-0.877802
C	2.745079	5.930855	1.620682
H	2.32869	6.844488	2.059973
H	3.505706	6.235963	0.892688
H	3.241837	5.376508	2.425894
C	0.566829	4.822681	2.075692
H	1.020028	4.22779	2.878884
H	-0.287067	4.278561	1.674498
H	0.20609	5.759827	2.517861
C	0.964083	0.937635	-1.834773
H	1.50107	0.176477	-2.399204
C	-1.013623	0.19983	-3.017803
C	-0.293854	-1.102548	-3.386887
H	-0.026046	-1.678953	-2.497192
H	0.607874	-0.923617	-3.981806
H	-0.960909	-1.720948	-3.996735
C	-1.212395	1.074567	-4.271985

H	-1.772696	1.982532	-4.029884
H	-1.759195	0.518738	-5.042534
H	-0.242826	1.372543	-4.684757
C	-2.361057	-0.133112	-2.37157
H	-3.04504	-0.495554	-3.142462
H	-2.237604	-0.928888	-1.624488
C	-4.165219	1.237998	-1.566465
C	-5.103671	0.132801	-2.026233
H	-4.692799	-0.851421	-1.792963
H	-5.276269	0.176131	-3.109631
H	-6.069363	0.199289	-1.529431
C	-4.733359	2.428803	-0.960878
C	-6.117961	2.697667	-1.133615
H	-6.692927	2.042832	-1.77351
C	-6.740672	3.781931	-0.554379
C	-5.935269	4.606013	0.267222
H	-6.417742	5.441383	0.759229
C	-4.578733	4.424224	0.479004
C	-3.920481	3.328097	-0.197956
C	-8.230414	4.112242	-0.748453
C	-8.935049	3.106084	-1.677986
H	-8.902341	2.086578	-1.275921
H	-8.488318	3.094134	-2.678968
H	-9.989891	3.380949	-1.79093
C	-8.951237	4.084484	0.620935
H	-8.879829	3.091502	1.079897
H	-10.014313	4.32828	0.501353
H	-8.522461	4.807619	1.32282
C	-8.375193	5.521413	-1.37095
H	-7.923672	6.294515	-0.740218
H	-9.434248	5.776302	-1.502534
H	-7.889159	5.566318	-2.35223
C	-4.694356	6.456643	2.040876
H	-5.494307	6.02561	2.654757
H	-5.151003	7.112569	1.290541
H	-4.083284	7.087956	2.695961
C	-3.795612	5.376162	1.404471
C	-3.168279	4.56778	2.56578
H	-2.588504	5.230344	3.220223
H	-2.505218	3.788618	2.188628
H	-3.953498	4.099354	3.173138
C	-2.699228	6.107263	0.596164
H	-2.133515	6.78252	1.249993

H	-3.150786	6.711587	-0.200309
H	-2.006007	5.399204	0.144126
C	-1.343401	-0.797686	4.150855
H	-0.375645	-1.07754	4.580455
H	-1.912295	-1.713933	3.966558
H	-1.883596	-0.190844	4.886843
C	3.024868	-1.554955	0.970925
H	3.427756	-0.714531	1.528198
C	1.623129	-1.746681	0.947639
C	0.829153	-0.863565	1.738032
H	1.381981	-0.111997	2.300115
C	1.900707	-3.773882	-0.413003
C	3.262423	-3.522026	-0.357711
H	3.931407	-4.201889	-0.870481
C	3.870903	-2.438944	0.330572
C	0.691822	-5.940147	-0.040971
H	1.432079	-6.274847	0.695976
H	0.256961	-6.830749	-0.511151
H	-0.100857	-5.407636	0.486379
C	2.481549	-5.872779	-1.769501
H	3.217403	-6.236192	-1.04285
H	3.013749	-5.310138	-2.545807
H	2.035319	-6.751767	-2.248353
C	1.364007	-5.041067	-1.107478
C	-8.305891	-4.182907	1.032617
C	-4.969141	-6.194799	-2.225704
H	-5.372104	-6.914547	-1.503759
H	-4.397676	-6.764946	-2.966941
H	-5.809975	-5.724289	-2.749438
C	0.355868	-4.668032	-2.217299
H	-0.481498	-4.098971	-1.814843
H	-0.035996	-5.575696	-2.69301
H	0.848152	-4.07127	-2.995677
C	7.385389	-3.772891	0.806469
C	5.995035	-3.632485	0.718385
H	5.361486	-4.496682	0.889821
C	5.355388	-2.418054	0.442286
C	6.17006	-1.273331	0.272209
C	7.562939	-1.370747	0.328489
C	8.140355	-2.621726	0.595732
H	9.221198	-2.678339	0.640986

B) Optimized XYZ Coordinates (Å) for **4**

Ni	6.73617	-0.563476	-0.637697
O	8.267387	-0.416685	0.364672
N	7.175195	-2.277581	-1.202471
C	9.381666	-1.091051	0.261076
Ni	-7.597416	0.840699	-0.319088
O	6.282581	1.133352	-0.034521
N	5.166563	-0.739171	-1.6044
C	10.567421	-0.621435	0.938052
N	-7.071724	2.341819	-1.260461
O	-5.903802	0.540235	0.365809
C	11.754841	-1.313151	0.746494
H	12.640196	-0.935153	1.237539
N	-9.347548	1.288142	-0.728026
O	-8.066013	-0.772854	0.405501
C	11.888021	-2.47593	-0.045248
C	10.728465	-2.96961	-0.608227
H	10.786512	-3.863197	-1.219447
C	9.47258	-2.329025	-0.459474
C	10.521645	0.615943	1.855808
C	10.11022	1.862324	1.040345
H	10.064561	2.743142	1.692783
H	9.134935	1.723228	0.576923
H	10.847308	2.065687	0.253317
C	9.518449	0.360274	3.007257
H	9.85191	-0.48304	3.624913
H	8.522762	0.135167	2.623596
H	9.449569	1.243796	3.653879
C	11.887281	0.923455	2.50448
H	12.657226	1.161763	1.760975
H	12.250637	0.095609	3.124929
H	11.78204	1.798673	3.155653
C	13.573238	-3.176808	-1.78673
H	13.652458	-2.150885	-2.164181
H	12.804143	-3.688713	-2.375565
H	14.528888	-3.682962	-1.973707
C	13.237162	-3.185046	-0.276347
C	13.15164	-4.6488	0.217141
H	14.105646	-5.166964	0.057129
H	12.376439	-5.212677	-0.31333
H	12.918475	-4.68806	1.287339
C	14.396611	-2.49971	0.472461
H	15.331477	-3.037311	0.276393
H	14.23828	-2.497918	1.556943

H	14.537766	-1.463044	0.14639
C	8.290952	-2.945819	-1.022119
C	8.340013	-4.423109	-1.383488
H	9.151395	-4.928179	-0.86334
H	8.47941	-4.575766	-2.461717
H	7.411848	-4.923835	-1.096293
C	6.01919	-2.940594	-1.817045
H	5.4035	-3.40744	-1.034656
H	6.315614	-3.717957	-2.525557
C	5.189419	-1.881394	-2.551522
C	5.906497	-1.424392	-3.837423
H	6.905098	-1.042469	-3.606657
H	5.339558	-0.623108	-4.322844
H	6.001126	-2.257025	-4.544285
C	3.811832	-2.467769	-2.892571
H	3.227715	-1.808096	-3.541743
H	3.228283	-2.677351	-1.989521
H	3.945868	-3.409558	-3.43569
C	4.117374	0.026061	-1.522895
H	3.238433	-0.240621	-2.109907
C	3.999306	1.211021	-0.744489
C	2.747113	1.866783	-0.739911
H	1.926833	1.425156	-1.301893
C	2.567431	3.061105	-0.072403
C	3.703769	3.606979	0.580621
H	3.558476	4.542756	1.106567
C	4.96033	3.023409	0.61051
C	5.133163	1.748585	-0.051665
C	6.136652	3.720039	1.318732
C	5.727178	5.055542	1.972986
H	6.605799	5.499307	2.454779
H	4.961827	4.922446	2.746703
H	5.354924	5.781301	1.240118
C	7.231115	4.039218	0.271392
H	8.100519	4.499934	0.756177
H	6.848802	4.745303	-0.476212
H	7.562997	3.137375	-0.244204
C	6.693458	2.810323	2.437646
H	7.022874	1.850753	2.041194
H	5.925514	2.628342	3.199924
H	7.545542	3.295492	2.929855
C	1.260035	3.762437	-0.051735
C	1.18612	5.163641	-0.131383

H	2.098227	5.747019	-0.219164
C	-0.050483	5.806343	-0.131433
H	-0.093302	6.891177	-0.190119
C	-1.231944	5.071696	-0.05352
H	-2.188633	5.585692	-0.021009
C	-1.194827	3.668894	0.025142
C	0.059392	3.039491	0.028848
H	0.104797	1.956184	0.097233
C	-2.445986	2.875111	0.110016
C	-2.530742	1.724494	0.938758
H	-1.651119	1.483026	1.523351
C	-3.654855	0.925481	1.073516
C	-4.830772	1.276422	0.308488
C	-4.761607	2.450134	-0.511236
C	-3.578073	3.218696	-0.60042
H	-3.567751	4.082294	-1.262262
C	-3.659213	-0.286194	2.024852
C	-2.317078	-0.458399	2.764801
H	-1.481756	-0.631635	2.075933
H	-2.383036	-1.331227	3.424142
H	-2.072821	0.407509	3.391255
C	-3.910649	-1.580454	1.215788
H	-3.107513	-1.738523	0.485012
H	-4.860762	-1.536037	0.684026
H	-3.928603	-2.447282	1.888163
C	-4.756703	-0.098638	3.100378
H	-4.782922	-0.968636	3.768094
H	-5.741792	0.018469	2.647481
H	-4.546121	0.787765	3.71141
C	-5.886849	2.880589	-1.269256
H	-5.72221	3.751099	-1.904759
C	-8.14811	2.871164	-2.136294
C	-8.169307	2.045496	-3.437525
H	-7.225597	2.168544	-3.979296
H	-8.986823	2.373309	-4.090226
H	-8.30021	0.982219	-3.217469
C	-8.032765	4.368176	-2.459593
H	-7.881584	4.964278	-1.553051
H	-8.957671	4.707000	-2.939051
H	-7.21764	4.581286	-3.158185
C	-9.424293	2.630205	-1.319271
H	-9.494447	3.388000	-0.525354
H	-10.295724	2.739395	-1.971468

C	-10.451104	0.651185	-0.402576
C	-11.783779	1.376437	-0.510089
H	-11.670877	2.445531	-0.321045
H	-12.492951	0.993106	0.222807
H	-12.232885	1.25782	-1.50484
C	-10.442668	-0.724948	0.042092
C	-11.668089	-1.445648	0.082279
H	-12.575708	-0.938325	-0.213192
C	-11.734599	-2.774366	0.436846
C	-9.274485	-2.782096	0.757467
C	-9.217091	-1.385536	0.38502
C	-7.992887	-3.559105	1.121252
C	-7.016865	-3.550062	-0.080579
H	-6.750725	-2.532907	-0.369215
H	-7.469824	-4.051277	-0.945268
H	-6.096931	-4.08991	0.176263
C	-7.329274	-2.921886	2.364633
H	-6.411248	-3.464865	2.622292
H	-8.003945	-2.974553	3.228263
H	-7.075061	-1.877944	2.184701
C	-8.278381	-5.036351	1.462446
H	-7.333654	-5.53369	1.709366
H	-8.724157	-5.579927	0.620784
H	-8.939053	-5.14186	2.331153
C	-13.043939	-3.580921	0.473563
C	-10.509764	-3.404409	0.770741
H	-10.547857	-4.450007	1.050888
C	-14.266848	-2.728204	0.085921
H	-14.401863	-1.878743	0.765679
H	-14.186549	-2.340672	-0.93652
H	-15.175884	-3.338303	0.136196
C	-13.276613	-4.12654	1.902992
H	-12.458593	-4.777091	2.229831
H	-13.358389	-3.306356	2.62543
H	-14.203577	-4.712112	1.94516
C	-12.953723	-4.767002	-0.516434
H	-12.123097	-5.437689	-0.272991
H	-13.877082	-5.359436	-0.493948
H	-12.80331	-4.409232	-1.541564

C) Optimized XYZ Coordinates (Å) for **5**

Ni	-8.30272	0.929721	-0.041449
N	9.496713	2.317873	-0.321525

C	11.744563	-1.326534	0.329845
Ni	8.302704	0.92974	-0.04157
C	12.976821	1.131801	-0.313912
H	13.442029	2.084771	-0.52464
O	9.618374	-0.341485	-0.026895
O	-9.618376	-0.341512	-0.026994
O	-7.091167	-0.470142	-0.084239
O	7.091163	-0.470137	-0.08422
N	-9.496713	2.317934	-0.32113
N	6.989217	2.206902	0.200965
N	-6.989214	2.20685	0.201189
C	10.913204	-0.18936	0.001949
C	11.556599	1.052862	-0.313032
C	10.785868	2.248591	-0.572323
C	8.79255	3.599977	-0.451174
H	8.48008	3.754629	-1.494151
H	9.423298	4.443759	-0.15631
C	-11.118327	-2.684195	0.707042
C	-10.913213	-0.18937	0.001853
C	11.496992	3.468268	-1.138211
H	11.88459	4.118862	-0.343404
H	10.828084	4.064852	-1.761095
H	12.337944	3.169035	-1.762964
C	-11.556579	1.052891	-0.312972
C	-11.744595	-1.326561	0.32961
C	13.777936	0.053325	-0.012326
C	7.548801	3.56032	0.446272
C	13.117447	-1.160238	0.303664
H	13.737522	-2.014299	0.547157
C	-13.117478	-1.160234	0.303424
H	-13.737586	-2.014296	0.546825
C	-10.785834	2.248658	-0.572064
C	-5.042005	-1.672751	-0.334544
C	-7.548761	3.560218	0.446818
C	-13.777938	0.053377	-0.012462
C	11.118314	-2.684134	0.707468
C	-12.976796	1.131869	-0.313915
H	-13.441968	2.084867	-0.524624
C	6.614796	4.717882	0.062699
H	7.174814	5.659075	0.089433
H	5.779028	4.827001	0.760886
H	6.212144	4.590133	-0.948037
C	-5.789364	-0.45344	-0.119844

C	-10.303205	-3.235199	-0.486487
H	-9.509861	-2.545999	-0.774029
H	-10.955342	-3.399544	-1.353366
H	-9.849219	-4.197972	-0.220395
C	-5.76294	-3.011717	-0.581092
C	-8.792589	3.600093	-0.450497
H	-9.423344	4.44376	-0.155304
H	-8.480244	3.755063	-1.493463
C	-11.497066	3.46836	-1.137762
H	-11.885794	4.118148	-0.342849
H	-12.337307	3.168995	-1.763439
H	-10.828013	4.065786	-1.759653
C	5.789358	-0.453425	-0.119808
C	10.211476	-2.516895	1.951191
H	9.75849	-3.479587	2.218838
H	9.411634	-1.799031	1.767547
H	10.799393	-2.172421	2.811251
C	5.043207	0.757319	0.05832
C	-15.314896	0.113059	0.005609
C	7.943234	3.6652	1.932554
H	8.644405	2.870819	2.203112
H	7.056523	3.567568	2.567743
H	8.411501	4.634357	2.140717
C	-2.910397	-0.403831	-0.132634
C	-10.211665	-2.51716	1.950917
H	-9.758538	-3.479851	2.218329
H	-10.799763	-2.173046	2.810999
H	-9.411915	-1.799113	1.767593
C	-5.043202	0.757299	0.058292
C	-3.658532	-1.595863	-0.329219
H	-3.091108	-2.500172	-0.513613
C	15.314891	0.112972	0.005712
C	-6.652325	-2.900029	-1.843432
H	-6.036862	-2.701584	-2.729708
H	-7.386283	-2.099384	-1.74426
H	-7.188454	-3.842405	-2.010919
C	5.704232	2.004157	0.243302
H	5.053863	2.859975	0.426413
C	12.182943	-3.742481	1.062733
H	12.849454	-3.961838	0.220099
H	11.679712	-4.678043	1.331589
H	12.797462	-3.443152	1.92023
C	-5.704223	2.004115	0.24339

H	-5.053851	2.859929	0.4265
C	-12.182878	-3.742748	1.061953
H	-11.679564	-4.678284	1.330743
H	-12.849224	-3.962069	0.219181
H	-12.797574	-3.443637	1.919401
C	10.303396	-3.235521	-0.48603
H	10.955635	-3.399945	-1.35282
H	9.509973	-2.546518	-0.773783
H	9.849558	-4.198322	-0.219777
C	-7.943059	3.664776	1.933164
H	-7.056303	3.566931	2.568256
H	-8.644265	2.870382	2.203594
H	-8.411239	4.633918	2.14159
C	-3.62973	0.759087	0.057482
H	-3.112332	1.699997	0.232698
C	3.629733	0.759114	0.05753
H	3.112326	1.700026	0.232718
C	-1.428812	-0.415372	-0.141666
C	5.042004	-1.672732	-0.334509
C	15.83606	-0.24169	1.418956
H	15.521133	-1.243089	1.731138
H	15.463451	0.47161	2.163173
H	16.932794	-0.216551	1.44201
C	-6.614758	4.717836	0.063421
H	-5.778953	4.826797	0.761586
H	-7.174756	5.659035	0.090374
H	-6.212163	4.59028	-0.947363
C	2.910408	-0.403808	-0.132592
C	3.658529	-1.595842	-0.329186
H	3.091092	-2.50015	-0.513551
C	1.428816	-0.415358	-0.141642
C	-6.621795	-3.372045	0.653947
H	-5.987271	-3.493309	1.540713
H	-7.146879	-4.320347	0.483975
H	-7.362141	-2.599186	0.858932
C	-4.778942	-4.175641	-0.815162
H	-4.128765	-4.350751	0.050286
H	-4.145764	-4.013986	-1.695501
H	-5.349249	-5.095582	-0.986263
C	5.762922	-3.011703	-0.581082
C	15.88464	-0.900281	-1.016043
H	16.98165	-0.880969	-1.005505
H	15.549081	-0.661315	-2.031768

H	15.568947	-1.925148	-0.794079
C	15.85012	1.509901	-0.361354
H	15.512984	2.274871	0.348025
H	15.539183	1.813821	-1.367847
H	16.945907	1.504398	-0.342982
C	-0.695975	-1.495014	0.381134
H	-1.224179	-2.332934	0.828217
C	6.652244	-2.899999	-1.843463
H	7.386398	-2.099546	-1.744219
H	6.036765	-2.701228	-2.729657
H	7.188134	-3.842467	-2.011178
C	-15.850088	1.510018	-0.361383
H	-15.512889	2.27495	0.348009
H	-16.945874	1.504568	-0.342964
H	-15.539174	1.813959	-1.367882
C	0.695988	-1.495006	0.381149
H	1.224206	-2.332925	0.828221
C	-0.695233	0.660404	-0.672699
H	-1.225432	1.492221	-1.129185
C	0.695242	0.660413	-0.672687
H	1.225454	1.492245	-1.129129
C	6.621821	-3.372092	0.653907
H	7.146731	-4.320494	0.483957
H	5.987355	-3.493194	1.540731
H	7.362307	-2.599335	0.858777
C	-15.884732	-0.900129	-1.016169
H	-15.549027	-0.661277	-2.031866
H	-16.981739	-0.880596	-1.005735
H	-15.569281	-1.925055	-0.794127
C	-15.836015	-0.24165	1.41886
H	-15.520991	-1.243027	1.731024
H	-16.932751	-0.216603	1.44194
H	-15.46345	0.471672	2.163076
C	4.778883	-4.175601	-0.81513
H	4.145806	-4.014023	-1.695557
H	4.128591	-4.350581	0.050259
H	5.349169	-5.095586	-0.986053

D) Optimized XYZ Coordinates (Å) for [**2**$^{••}$]$^{2+}$ (*S* = 1)

Ni	-1.610945	1.87996	-1.183545
O	-2.850839	2.825319	-0.237004
N	-2.907985	0.836638	-1.990883
C	-4.132749	2.966754	-0.440169

Ni	-1.393786	-2.029822	1.296038
O	-0.321096	2.876355	-0.329074
N	-0.352862	0.921332	-2.133142
C	-4.861653	4.00846	0.257269
N	-0.157109	-1.080957	2.284354
O	-0.073585	-3.010769	0.457385
C	-6.197195	4.176376	-0.064203
H	-6.740077	4.972199	0.427483
N	-2.707713	-1.004186	2.097909
O	-2.602694	-2.960049	0.301391
C	-6.911274	3.390055	-1.002944
C	-6.22423	2.336834	-1.586084
H	-6.739412	1.709654	-2.299312
O	5.506409	0.090954	-0.017723
C	-4.860978	2.088159	-1.318585
C	-4.18693	4.921077	1.297674
C	-3.073911	5.748037	0.614499
H	-2.306721	5.107305	0.18233
H	-2.600341	6.412377	1.34641
H	-3.493849	6.37458	-0.180635
C	-3.609039	4.059221	2.446602
H	-2.890203	3.324962	2.077942
H	-4.41499	3.53375	2.97438
H	-3.100602	4.699789	3.175762
C	0.973393	2.763333	-0.288038
C	-8.461206	5.160057	-1.884315
H	-7.884671	5.256177	-2.810907
H	-8.086647	5.90493	-1.174687
H	-9.504034	5.411332	-2.106566
C	-8.980521	2.756549	-2.35897
H	-8.449122	2.799141	-3.316773
H	-10.021299	3.033769	-2.552743
H	-8.981554	1.718171	-2.006286
C	7.433558	5.905865	-1.863747
H	7.741762	5.304007	-2.725953
H	7.856757	6.909335	-1.983736
H	6.342697	6.001652	-1.892653
C	7.481546	6.162117	0.65048
H	7.90821	7.166282	0.551781
H	7.820296	5.743273	1.604718
H	6.392273	6.270861	0.697084
C	7.927442	5.2767	-0.538415
C	-4.215819	0.944289	-1.93157

C	-5.083736	-0.162523	-2.503519
H	-5.198924	-0.061076	-3.590284
H	-6.076567	-0.153409	-2.058292
H	-4.650201	-1.144503	-2.302403
C	-2.302507	-0.307091	-2.693995
H	-2.93309	-0.659204	-3.511529
H	-2.163101	-1.133401	-1.984597
C	-0.949346	0.137121	-3.254396
C	-0.139044	-1.095189	-3.673664
H	-0.740187	-1.69942	-4.360407
H	0.120638	-1.723691	-2.816106
H	0.77527	-0.826815	-4.211819
C	-9.216557	3.625637	-0.021943
H	-10.262199	3.870281	-0.238018
H	-8.869731	4.321662	0.748791
H	-9.186282	2.613436	0.397391
C	9.467373	5.243096	-0.564229
H	9.851354	4.658445	-1.407985
H	9.886072	4.833472	0.362209
H	9.852948	6.261718	-0.673156
C	8.456628	0.215397	-0.089385
C	9.365389	0.36257	1.1609
H	10.04521	-0.489883	1.253048
H	8.767562	0.421886	2.075976
H	9.979332	1.26585	1.095875
C	9.333152	0.143021	-1.368727
H	8.712974	0.033475	-2.264088
H	10.022325	-0.70574	-1.325487
H	9.937617	1.047844	-1.481838
C	7.639318	-1.071159	0.035022
C	6.238243	-1.069173	0.077805
C	5.507426	-2.282675	0.181116
C	6.239588	-3.486453	0.246468
H	5.681551	-4.405342	0.369355
C	7.629176	-3.531648	0.22427
C	8.295434	-2.299142	0.111281
H	9.379507	-2.2976	0.085153
C	9.292559	-5.005004	-0.951758
H	8.663528	-5.063635	-1.84712
H	9.879666	-5.927833	-0.889348
H	9.995923	-4.176697	-1.08818
C	8.433973	-4.837466	0.325864
C	7.525115	-6.072978	0.464324

H	6.863468	-6.195055	-0.401401
H	6.909391	-6.030093	1.370285
H	8.141507	-6.974918	0.531876
C	9.360398	-4.775174	1.564667
H	9.941091	-5.700669	1.644763
H	8.78041	-4.658579	2.487026
H	10.072847	-3.945368	1.507238
C	1.212035	-2.85037	0.406891
C	2.049004	-3.768122	-0.342339
C	3.397857	-3.475785	-0.407808
H	4.03105	-4.127017	-0.994666
C	4.038257	-2.375386	0.234041
C	3.232935	-1.527919	0.993543
H	3.67522	-0.709117	1.55093
C	1.85501	-1.76422	1.106169
C	1.484263	-5.040424	-0.997108
C	0.872501	-5.935435	0.110674
H	0.438584	-6.836108	-0.337183
H	0.088063	-5.420922	0.667575
H	1.64622	-6.253866	0.818576
C	2.574279	-5.869956	-1.705995
H	2.112677	-6.762729	-2.139476
H	3.352604	-6.212641	-1.015032
H	3.051393	-5.321129	-2.526369
C	0.422952	-4.671672	-2.057739
H	0.878163	-4.09238	-2.870601
H	-0.394788	-4.095199	-1.626061
H	0.005000	-5.583694	-2.498362
C	1.117977	-0.990683	2.056562
H	1.70396	-0.305728	2.666804
C	-0.77716	-0.369893	3.439257
C	0.018584	0.83384	3.955851
H	0.27887	1.528939	3.151383
H	0.931961	0.531444	4.477372
H	-0.591971	1.378883	4.682693
C	-0.976173	-1.401545	4.568573
H	-1.578742	-2.249972	4.231096
H	-1.478655	-0.934299	5.421928
H	-0.009112	-1.784271	4.909133
C	-2.123292	0.0989	2.880614
H	-2.772153	0.395177	3.706589
H	-1.97432	0.970103	2.228599
C	-4.014055	-1.088108	1.972596

C	-4.887623	0.012809	2.547643
H	-4.41205	0.989612	2.443431
H	-5.0886	-0.151888	3.614091
H	-5.844274	0.064067	2.031237
C	-4.650497	-2.19987	1.297696
C	-6.036877	-2.413356	1.480715
H	-6.577893	-1.773147	2.162253
C	-6.713999	-3.446541	0.85752
C	-5.959892	-4.263559	-0.020829
H	-6.487818	-5.056213	-0.533404
C	-4.602537	-4.130815	-0.259613
C	-3.895685	-3.089686	0.456992
C	-8.207863	-3.7287	1.072508
C	-8.85478	-2.734178	2.054722
H	-8.794118	-1.700577	1.692857
H	-8.396596	-2.782012	3.049403
H	-9.915803	-2.974483	2.1743
C	-8.949805	-3.624349	-0.283304
H	-8.852972	-2.619572	-0.711106
H	-10.01674	-3.829125	-0.142574
H	-8.572633	-4.342506	-1.018749
C	-8.38124	-5.158089	1.643476
H	-7.979175	-5.923106	0.971239
H	-9.444944	-5.374908	1.790672
H	-7.877466	-5.260538	2.61092
C	-4.850363	-6.097061	-1.884912
H	-5.647372	-5.611895	-2.459937
H	-5.309665	-6.763355	-1.146566
H	-4.286697	-6.727414	-2.580326
C	-3.886016	-5.081069	-1.238141
C	-3.256943	-4.258961	-2.388564
H	-2.727579	-4.923954	-3.080252
H	-2.545846	-3.521484	-2.012643
H	-4.037309	-3.740716	-2.960723
C	-2.806018	-5.889533	-0.482375
H	-2.306205	-6.579786	-1.171552
H	-3.26246	-6.488148	0.314187
H	-2.053665	-5.238596	-0.03881
C	-1.132132	1.091151	-4.451983
H	-0.161594	1.463798	-4.793941
H	-1.751925	1.952649	-4.185925
H	-1.607439	0.563335	-5.285426
C	3.047934	1.556075	-0.942799

H	3.51698	0.769872	-1.525289
C	1.652076	1.715945	-1.008417
C	0.930317	0.89448	-1.925444
H	1.532011	0.216212	-2.527716
C	1.77808	3.698143	0.471863
C	3.143078	3.478368	0.493567
H	3.757345	4.149404	1.078559
C	3.825799	2.438538	-0.201319
C	0.540127	5.837987	0.084808
H	1.312145	6.206687	-0.60015
H	0.067787	6.707425	0.554994
H	-0.216163	5.314575	-0.502008
C	2.226011	5.765778	1.916134
H	2.989217	6.172478	1.243329
H	2.726417	5.198334	2.709507
H	1.729934	6.618433	2.390453
C	1.168725	4.926638	1.169389
C	-8.37592	3.719533	-1.320032
C	-5.176361	5.918061	1.935881
H	-5.592254	6.619005	1.203955
H	-4.644183	6.51414	2.68425
H	-6.004997	5.416093	2.448254
C	0.113447	4.490968	2.210654
H	-0.681555	3.898378	1.75875
H	-0.338162	5.37442	2.675261
H	0.584501	3.905576	3.01002
C	7.31585	3.873424	-0.384045
C	5.929849	3.709893	-0.332929
H	5.294361	4.582865	-0.429651
C	5.303618	2.457991	-0.221533
C	6.13785	1.310591	-0.151532
C	7.530037	1.427758	-0.183685
C	8.084731	2.707548	-0.30198
H	9.16382	2.788656	-0.332929

E) Optimized XYZ Coordinates (Å) for $[\mathbf{2}^{\bullet\bullet}]^{2+}$ (S = 0)

Ni	-1.234738	2.928193	-1.351782
O	-2.746232	3.115957	-0.356465
N	-2.191131	2.864124	-2.935438
C	-3.951613	3.462569	-0.70665
Ni	-1.240499	-2.934028	1.350303
O	-0.294733	2.944664	0.233624
N	0.297446	2.72414	-2.357825

C	-4.939742	3.748829	0.315521
N	0.291633	-2.730167	2.357715
O	-0.297081	-2.95229	-0.234989
C	-6.163105	4.240722	-0.101496
H	-6.895018	4.488901	0.65526
N	-2.197631	-2.871064	2.932949
O	-2.750189	-3.117392	0.352497
C	-6.533991	4.449335	-1.454257
C	-5.620651	4.056758	-2.420479
H	-5.879287	4.180309	-3.462262
O	5.272365	-0.007468	0.006668
C	-4.349788	3.541466	-2.088601
C	-4.647839	3.528039	1.80998
C	-3.519236	4.48118	2.264909
H	-2.603371	4.315423	1.699674
H	-3.304803	4.325296	3.328561
H	-3.823219	5.526427	2.136712
C	-4.254269	2.047779	2.044135
H	-3.380509	1.759085	1.457808
H	-5.085741	1.38406	1.777742
H	-4.026389	1.890167	3.104929
C	0.953002	2.693491	0.499825
C	-8.001804	6.464314	-1.142127
H	-7.2304	7.135505	-1.535453
H	-7.894442	6.427675	-0.05309
H	-8.979681	6.905905	-1.362753
C	-8.124951	5.203987	-3.303604
H	-7.378878	5.859585	-3.767578
H	-9.107779	5.64838	-3.488874
H	-8.105658	4.235172	-3.816837
C	7.566999	5.34345	2.707976
H	8.004433	5.455786	1.709591
H	8.015235	6.101098	3.360463
H	6.496431	5.563049	2.632446
C	7.207783	3.817976	4.689269
H	7.65611	4.562267	5.356628
H	7.382491	2.82664	5.122394
H	6.12687	3.995764	4.67726
C	7.830286	3.927836	3.276681
C	-3.472845	3.071447	-3.140823
C	-4.066862	2.782447	-4.507963
H	-3.881026	3.606195	-5.208965
H	-5.141916	2.627989	-4.447463

H	-3.636546	1.875121	-4.937594
C	-1.337622	2.426087	-4.052634
H	-1.702711	2.794763	-5.01222
H	-1.325305	1.328246	-4.087243
C	0.078469	2.955452	-3.815695
C	1.059573	2.216317	-4.73554
H	0.706516	2.292972	-5.768858
H	1.131981	1.152873	-4.48252
H	2.061189	2.655976	-4.714847
C	-9.020317	4.151165	-1.215142
H	-10.00109	4.582998	-1.442422
H	-8.952804	4.044662	-0.127446
H	-8.98108	3.149017	-1.656844
C	9.353166	3.734201	3.406694
H	9.867542	3.852493	2.446234
H	9.608111	2.750505	3.817689
H	9.761055	4.488503	4.087152
C	8.222247	-0.011154	0.010757
C	9.108344	-0.835562	0.981695
H	9.75207	-1.52742	0.429477
H	8.494146	-1.420954	1.673398
H	9.758256	-0.182156	1.571724
C	9.125579	0.805235	-0.951036
H	8.52381	1.3952	-1.649732
H	9.776668	0.146253	-1.53357
H	9.769446	1.491831	-0.392695
C	7.346397	-0.979958	-0.793965
C	5.952661	-0.981507	-0.681057
C	5.154565	-1.969172	-1.303367
C	5.793653	-2.887748	-2.154828
H	5.182976	-3.650756	-2.622011
C	7.174807	-2.888103	-2.351887
C	7.92094	-1.935485	-1.635293
H	9.001189	-1.941652	-1.735506
C	8.637179	-3.116557	-4.384506
H	7.93828	-2.537477	-4.998802
H	9.165229	-3.816694	-5.041492
H	9.380152	-2.42324	-3.976269
C	7.891115	-3.892467	-3.271848
C	6.908976	-4.871257	-3.94239
H	6.171641	-4.350398	-4.564512
H	6.373381	-5.484068	-3.207949
H	7.461354	-5.554596	-4.595298

C	8.909447	-4.716628	-2.447569
H	9.419403	-5.438866	-3.094521
H	8.41159	-5.273221	-1.645643
H	9.678735	-4.084495	-1.991656
C	0.949926	-2.701358	-0.499425
C	1.461902	-2.779414	-1.854208
C	2.803661	-2.493096	-2.046256
H	3.202335	-2.545327	-3.051373
C	3.714183	-2.121395	-1.019713
C	3.213751	-2.049685	0.276719
H	3.875382	-1.810139	1.102332
C	1.872749	-2.353875	0.549382
C	0.569003	-3.200945	-3.032774
C	0.089579	-4.655555	-2.799776
H	-0.579854	-4.965248	-3.609894
H	-0.445247	-4.761437	-1.855053
H	0.943904	-5.341928	-2.790857
C	1.320535	-3.175875	-4.379589
H	0.632015	-3.476563	-5.175894
H	2.162991	-3.87578	-4.401785
H	1.694171	-2.175592	-4.630111
C	-0.630079	-2.231836	-3.156431
H	-0.275136	-1.211123	-3.354814
H	-1.23597	-2.217704	-2.251314
H	-1.26726	-2.53306	-3.995578
C	1.478569	-2.437977	1.917849
H	2.271097	-2.27968	2.647209
C	0.07137	-2.963472	3.81504
C	1.051556	-2.225971	4.737082
H	1.123836	-1.161956	4.486362
H	2.053376	-2.665197	4.716246
H	0.697727	-2.304811	5.769967
C	0.139756	-4.483771	4.062984
H	-0.58621	-5.018741	3.443432
H	-0.067329	-4.706185	5.115064
H	1.137601	-4.864673	3.824031
C	-1.345242	-2.435198	4.051615
H	-1.710674	-2.806221	5.010211
H	-1.333044	-1.337397	4.08925
C	-3.480164	-3.077435	3.136273
C	-4.074421	-2.790593	4.503885
H	-3.643855	-1.884173	4.935248
H	-3.889002	-3.615554	5.203568

H	-5.149387	-2.63572	4.443513
C	-4.356678	-3.543568	2.082672
C	-5.629945	-4.056537	2.412786
H	-5.889286	-4.181486	3.454243
C	-6.542967	-4.445163	1.445808
C	-6.169471	-4.236115	0.093875
H	-6.900846	-4.481869	-0.664251
C	-4.944167	-3.747039	-0.321029
C	-3.956835	-3.463587	0.701888
C	-7.91336	-5.051517	1.775475
C	-8.138854	-5.197174	3.292
H	-8.117448	-4.228703	3.805814
H	-7.39506	-5.854949	3.756547
H	-9.123134	-5.639052	3.475759
C	-9.028586	-4.140947	1.203175
H	-8.987474	-3.139247	1.645788
H	-10.010951	-4.570366	1.428307
H	-8.958901	-4.033556	0.115672
C	-8.015893	-6.456358	1.130112
H	-7.906453	-6.419251	0.041274
H	-8.995214	-6.895871	1.348656
H	-7.246721	-7.129676	1.52421
C	-5.880156	-3.808617	-2.702298
H	-6.733782	-3.171052	-2.445059
H	-6.200248	-4.855202	-2.653301
H	-5.61954	-3.602265	-3.745639
C	-4.649666	-3.526844	-1.815298
C	-4.251987	-2.047698	-2.049175
H	-4.021475	-1.890673	-3.109561
H	-3.378911	-1.761337	-1.460684
H	-5.082522	-1.382047	-1.784495
C	-3.52289	-4.482749	-2.268734
H	-3.305663	-4.327138	-3.331924
H	-3.830132	-5.527168	-2.141537
H	-2.607882	-4.319888	-1.701234
C	0.146656	4.475259	-4.066529
H	1.144167	4.856844	-3.827331
H	-0.579923	5.011446	-3.448695
H	-0.059399	4.695688	-5.119226
C	3.219567	2.047594	-0.27443
H	3.882753	1.811514	-1.09988
C	1.877534	2.350188	-0.548171
C	1.484754	2.434359	-1.916228

H	2.277932	2.277288	-2.645095
C	1.462103	2.767292	1.855246
C	2.803928	2.479992	2.048708
H	3.201048	2.528289	3.054721
C	3.716492	2.114268	1.022489
C	0.090418	4.641825	2.804221
H	0.94547	5.327329	2.798756
H	-0.580187	4.950068	3.613933
H	-0.442399	4.75100	1.858709
C	1.317237	3.1563	4.381712
H	2.16023	3.855443	4.407379
H	1.689773	2.15504	4.629817
H	0.627788	3.45518	5.177894
C	0.567784	3.186037	3.03382
C	-7.902076	5.058748	-1.786643
C	-5.879061	3.813021	2.694935
H	-6.196336	4.860452	2.645532
H	-5.62054	3.605978	3.738608
H	-6.733847	3.177523	2.436495
C	-0.632506	2.217955	3.153123
H	-1.23702	2.206872	2.247043
H	-1.27058	2.517571	3.992182
H	-0.279125	1.196315	3.349128
C	7.184319	2.879744	2.352129
C	5.798628	2.88038	2.150382
H	5.192559	3.652041	2.613926
C	5.15799	1.961699	1.30885
C	5.95693	0.968094	0.691595
C	7.346558	0.965042	0.805935
C	7.92776	1.925692	1.645213
H	9.006074	1.923722	1.744698

F) Optimized XYZ Coordinates (Å) for $[\mathbf{4}^{\bullet\bullet}]^{2+}$ (S = 1)

O	-8.367722	-0.40676	-0.363623
N	-7.464186	-2.202485	1.365483
C	-9.510357	-1.017462	-0.326817
Ni	7.693338	0.844791	0.343555
O	-6.304505	1.007395	-0.035166
N	-5.384693	-0.753468	1.734067
C	-10.604222	-0.550495	-1.160389
N	7.154765	2.338062	1.268777
O	6.005608	0.467085	-0.247146
C	-11.847597	-1.136156	-0.968907

H	-12.673703	-0.76772	-1.558086
N	9.439868	1.380171	0.61827
O	8.192691	-0.774551	-0.302404
C	-12.107961	-2.181342	-0.056201
C	-11.012874	-2.705372	0.636325
H	-11.180975	-3.533377	1.312641
C	-9.722731	-2.176797	0.51054
C	-10.408297	0.552674	-2.21098
C	-10.031556	1.877487	-1.506785
H	-9.898859	2.667478	-2.254173
H	-9.107713	1.782714	-0.938749
H	-10.830866	2.193255	-0.826183
C	-9.306515	0.116245	-3.210315
H	-9.609334	-0.791397	-3.745332
H	-8.353998	-0.076503	-2.714871
H	-9.150716	0.904721	-3.954624
C	-11.688878	0.813162	-3.031915
H	-12.512701	1.190033	-2.415377
H	-12.031513	-0.079859	-3.566765
H	-11.473944	1.579284	-3.783598
C	-13.894358	-2.57533	1.661782
H	-13.911914	-1.515458	1.93821
H	-13.199145	-3.089309	2.334524
H	-14.892896	-2.988577	1.841129
C	-13.511369	-2.75959	0.171124
C	-13.508171	-4.270287	-0.175687
H	-14.507395	-4.688754	-0.014229
H	-12.810519	-4.838723	0.449027
H	-13.23743	-4.436261	-1.224113
C	-14.581565	-2.066789	-0.693573
H	-15.559903	-2.510646	-0.484884
H	-14.390000	-2.191613	-1.765192
H	-14.656393	-0.995475	-0.475903
C	-8.610434	-2.815287	1.195675
C	-8.788004	-4.238572	1.692
H	-9.592501	-4.743804	1.161528
H	-9.018289	-4.263792	2.764836
H	-7.880863	-4.824725	1.530909
C	-6.383667	-2.874491	2.108555
H	-5.773941	-3.455547	1.40376
H	-6.774229	-3.554429	2.867059
C	-5.522045	-1.803732	2.783585
C	-6.259951	-1.182677	3.986154

H	-7.230244	-0.773852	3.688206
H	-5.667603	-0.369439	4.416833
H	-6.422246	-1.936551	4.763895
C	-4.196951	-2.432719	3.235982
H	-3.605085	-1.752486	3.855661
H	-3.589714	-2.758709	2.384625
H	-4.410602	-3.311454	3.852972
C	-4.286669	-0.057764	1.642189
H	-3.456665	-0.331764	2.291131
C	-4.067572	1.061538	0.79482
C	-2.796313	1.67233	0.820475
H	-2.018515	1.240859	1.445384
C	-2.556228	2.832353	0.105841
C	-3.641346	3.381828	-0.630006
H	-3.439979	4.287518	-1.187305
C	-4.913089	2.838957	-0.702464
C	-5.145569	1.603255	0.013627
C	-6.024776	3.539134	-1.501997
C	-5.533447	4.833839	-2.181741
H	-6.369599	5.287029	-2.723952
H	-4.737235	4.645778	-2.911125
H	-5.177397	5.576192	-1.458211
C	-7.163743	3.935914	-0.529953
H	-7.985893	4.398884	-1.08695
H	-6.804446	4.666888	0.203804
H	-7.558842	3.074602	0.010719
C	-6.538125	2.600219	-2.61834
H	-6.938406	1.671326	-2.214411
H	-5.729488	2.356351	-3.317607
H	-7.330446	3.099032	-3.187692
C	-1.241197	3.516545	0.121467
C	-1.1735	4.920467	0.17489
H	-2.087963	5.503842	0.221617
C	0.058757	5.568669	0.202176
H	0.097639	6.653189	0.242581
C	1.240575	4.833594	0.169109
H	2.193836	5.353023	0.148588
C	1.209154	3.428236	0.110998
C	-0.041312	2.788778	0.090993
H	-0.084402	1.704261	0.043749
C	2.47165	2.655143	0.046387
C	2.57249	1.480264	-0.747449
H	1.688999	1.191221	-1.302216

C	3.718913	0.715842	-0.894145
C	4.89679	1.147849	-0.175437
C	4.81166	2.339892	0.62136
C	3.608518	3.063823	0.725417
H	3.581607	3.945907	1.359399
C	3.737242	-0.526588	-1.801985
C	2.378085	-0.764666	-2.491587
H	1.571002	-0.941968	-1.770926
H	2.452201	-1.65703	-3.121461
H	2.088776	0.068679	-3.141918
C	4.050181	-1.780208	-0.949942
H	3.277691	-1.931196	-0.186249
H	5.016717	-1.700019	-0.45236
H	4.065923	-2.669496	-1.590102
C	4.792509	-0.340708	-2.920256
H	4.81593	-1.229744	-3.56054
H	5.793196	-0.185722	-2.514857
H	4.537519	0.518378	-3.551683
C	5.951121	2.831714	1.319216
H	5.787425	3.711776	1.938993
C	8.258805	2.937692	2.076872
C	8.389903	2.138887	3.388278
H	7.472884	2.225615	3.979398
H	9.219628	2.527884	3.987685
H	8.569903	1.077553	3.192233
C	8.081914	4.432948	2.374857
H	7.850154	5.00344	1.469112
H	9.013777	4.827191	2.792656
H	7.302759	4.61826	3.120356
C	9.490581	2.741068	1.185171
H	9.480201	3.478997	0.372147
H	10.39717	2.890916	1.77556
C	10.548881	0.796227	0.222057
C	11.846184	1.581103	0.203789
H	11.670764	2.645538	0.046345
H	12.490993	1.239158	-0.605574
H	12.398109	1.463663	1.145096
C	10.575789	-0.600154	-0.172904
C	11.813297	-1.256263	-0.309851
H	12.725239	-0.700662	-0.149587
C	11.904227	-2.612214	-0.603248
C	9.428257	-2.765718	-0.656854
C	9.354936	-1.347082	-0.362715

C	8.161648	-3.619373	-0.843283
C	7.285707	-3.554933	0.43361
H	6.963854	-2.537806	0.661164
H	7.832505	-3.949318	1.298274
H	6.391998	-4.17376	0.295704
C	7.37597	-3.112305	-2.077345
H	6.479684	-3.726312	-2.220374
H	7.985575	-3.195788	-2.984556
H	7.065297	-2.074113	-1.961788
C	8.496643	-5.105277	-1.089304
H	7.562229	-5.664458	-1.200406
H	9.04605	-5.552742	-0.25323
H	9.073837	-5.256301	-2.008333
C	13.240895	-3.350794	-0.744239
C	10.688513	-3.323778	-0.765684
H	10.759611	-4.379715	-0.987518
C	14.451259	-2.42399	-0.524696
H	14.483618	-1.608142	-1.256278
H	14.461559	-1.991562	0.482742
H	15.375701	-2.998352	-0.639387
C	13.338574	-3.950672	-2.17021
H	12.531361	-4.660592	-2.377776
H	13.303678	-3.165538	-2.933515
H	14.286543	-4.4884	-2.280466
C	13.307156	-4.493352	0.300617
H	12.502108	-5.223841	0.169857
H	14.25704	-5.029705	0.20123
H	13.245421	-4.100633	1.321613

G) Optimized XYZ Coordinates (Å) for [**4**••]$^{2+}$ (S = 0)

Ni	-6.889619	-0.571873	0.689271
O	-8.368773	-0.408093	-0.364031
N	-7.469268	-2.197844	1.373352
C	-9.511883	-1.017652	-0.326683
Ni	7.693952	0.8436	0.346053
O	-6.304997	1.005668	-0.037741
N	-5.38937	-0.748855	1.739861
C	-10.604051	-0.5532	-1.163982
N	7.154028	2.334878	1.273551
O	6.007156	0.466262	-0.246891
C	-11.848291	-1.136802	-0.971784
H	-12.673185	-0.770104	-1.563739
N	9.4401	1.379154	0.62339

O	8.194787	-0.77438	-0.302435
C	-12.11097	-2.177813	-0.055007
C	-11.017318	-2.700149	0.641264
H	-11.187195	-3.525285	1.320637
C	-9.726566	-2.173418	0.515113
C	-10.405401	0.545085	-2.219188
C	-10.028874	1.872756	-1.520229
H	-9.894359	2.659316	-2.270897
H	-9.106033	1.779904	-0.950248
H	-10.829114	2.192098	-0.842396
C	-9.302194	0.103291	-3.214565
H	-9.604628	-0.806715	-3.745764
H	-8.350638	-0.087577	-2.716564
H	-9.14471	0.88813	-3.962346
C	-11.684394	0.802823	-3.043439
H	-12.508903	1.18325	-2.430009
H	-12.026888	-0.092408	-3.574676
H	-11.467617	1.565265	-3.798324
C	-13.900293	-2.561265	1.66214
H	-13.917439	-1.49997	1.933071
H	-13.206434	-3.072321	2.338489
H	-14.899409	-2.972823	1.842133
C	-13.515279	-2.753535	0.172983
C	-13.513148	-4.265988	-0.166094
H	-14.513122	-4.682424	-0.004081
H	-12.81719	-4.832066	0.462631
H	-13.240925	-4.437608	-1.213223
C	-14.58344	-2.064065	-0.696867
H	-15.562535	-2.505886	-0.487423
H	-14.390356	-2.194497	-1.76755
H	-14.657545	-0.991596	-0.484727
C	-8.61574	-2.810316	1.204291
C	-8.795329	-4.231606	1.70557
H	-9.599498	-4.738039	1.175731
H	-9.02723	-4.252719	2.778147
H	-7.888473	-4.819144	1.547965
C	-6.390324	-2.86782	2.120575
H	-5.779835	-3.451682	1.418772
H	-6.782497	-3.544826	2.88086
C	-5.529147	-1.795089	2.793054
C	-6.268576	-1.169208	3.992163
H	-7.238169	-0.760933	3.691183
H	-5.676457	-0.3547	4.420745

H	-6.432488	-1.920129	4.772415
C	-4.205171	-2.42331	3.249853
H	-3.613825	-1.741207	3.867963
H	-3.596793	-2.752803	2.400656
H	-4.420346	-3.299664	3.869693
C	-4.290849	-0.053872	1.647487
H	-3.462244	-0.3259	2.299034
C	-4.06963	1.062111	0.796496
C	-2.798076	1.672656	0.82262
H	-2.021711	1.243304	1.450765
C	-2.556152	2.829642	0.104013
C	-3.639344	3.376472	-0.636517
H	-3.436311	4.279928	-1.196843
C	-4.911168	2.833729	-0.709661
C	-5.145674	1.601179	0.010962
C	-6.020805	3.531179	-1.514441
C	-5.527172	4.822458	-2.199044
H	-6.361913	5.273865	-2.744919
H	-4.729717	4.630413	-2.926036
H	-5.17188	5.567808	-1.478243
C	-7.161385	3.933039	-0.54639
H	-7.982286	4.393776	-1.107077
H	-6.803134	4.667257	0.184634
H	-7.557883	3.074428	-0.002428
C	-6.532792	2.587758	-2.627608
H	-6.935215	1.661306	-2.220224
H	-5.722854	2.339571	-3.323845
H	-7.323094	3.085019	-3.20111
C	-1.240864	3.513857	0.119959
C	-1.173431	4.917697	0.172537
H	-2.088084	5.500893	0.218137
C	0.05873	5.566079	0.20021
H	0.097498	6.65063	0.239975
C	1.240624	4.831034	0.168244
H	2.193914	5.350443	0.14797
C	1.209295	3.425774	0.110952
C	-0.041022	2.786121	0.090663
H	-0.084004	1.701551	0.044076
C	2.472153	2.652806	0.046967
C	2.57413	1.479368	-0.748762
H	1.69124	1.191075	-1.304884
C	3.720916	0.715385	-0.895481
C	4.897845	1.146528	-0.174913

C	4.811529	2.336983	0.62398
C	3.607959	3.060481	0.728089
H	3.580114	3.94148	1.363549
C	3.740412	-0.525559	-1.805348
C	2.381783	-0.763206	-2.496143
H	1.574366	-0.941938	-1.776216
H	2.456674	-1.654592	-3.127311
H	2.092472	0.070986	-3.145384
C	4.053491	-1.780449	-0.955256
H	3.280466	-1.933305	-0.192479
H	5.019553	-1.700461	-0.456718
H	4.07037	-2.668605	-1.59696
C	4.796255	-0.337257	-2.922677
H	4.820561	-1.225219	-3.564422
H	5.796603	-0.18236	-2.516397
H	4.541185	0.522735	-3.552838
C	5.950048	2.827896	1.323794
H	5.785458	3.706721	1.945083
C	8.257003	2.933343	2.083918
C	8.387118	2.132075	3.393927
H	7.469447	2.217247	3.984263
H	9.216053	2.520249	3.994962
H	8.567777	1.071192	3.196025
C	8.07924	4.427964	2.384596
H	7.848233	5.000116	1.479705
H	9.010509	4.82174	2.804163
H	7.299214	4.611543	3.129609
C	9.48976	2.738951	1.1931
H	9.479949	3.478485	0.381535
H	10.395721	2.887868	1.784681
C	10.549705	0.796584	0.226926
C	11.846674	1.582051	0.211181
H	11.670924	2.646773	0.056077
H	12.492063	1.242204	-0.598615
H	12.398158	1.462688	1.152507
C	10.577626	-0.59906	-0.170784
C	11.815503	-1.254252	-0.307997
H	12.727061	-0.698537	-0.145958
C	11.907339	-2.609644	-0.603983
C	9.431508	-2.764275	-0.660033
C	9.357257	-1.346243	-0.363042
C	8.165461	-3.618106	-0.8494
C	7.288514	-3.556939	0.426963

H	6.96588	-2.540497	0.656463
H	7.834886	-3.952886	1.291181
H	6.395274	-4.175981	0.287026
C	7.380514	-3.108589	-2.08292
H	6.484522	-3.72256	-2.22791
H	7.990781	-3.189916	-2.989883
H	7.069392	-2.07076	-1.965335
C	8.501381	-5.103271	-1.098584
H	7.567341	-5.662639	-1.211846
H	9.050253	-5.552454	-0.263078
H	9.079453	-5.25184	-2.017462
C	13.244505	-3.347228	-0.745208
C	10.692137	-3.321497	-0.768885
H	10.763938	-4.376953	-0.992784
C	14.454203	-2.420207	-0.522897
H	14.486638	-1.60282	-1.25276
H	14.463596	-1.989856	0.48544
H	15.379034	-2.993833	-0.638126
C	13.34375	-3.944435	-2.172198
H	12.537167	-4.654491	-2.381725
H	13.309018	-3.157948	-2.934113
H	14.292146	-4.481375	-2.282605
C	13.310402	-4.49169	0.297603
H	12.505796	-5.222292	0.164781
H	14.260621	-5.027409	0.198024
H	13.247613	-4.100884	1.319268

H) Optimized XYZ Coordinates (Å) for [**5**$^{\bullet\bullet}$]$^{2+}$ (S = 1)

Ni	-8.303818	0.960777	-0.034586
N	9.537862	2.306531	-0.320046
C	11.65657	-1.416115	0.372555
Ni	8.303818	0.960791	-0.034521
C	12.962833	0.9999	-0.364523
H	13.470231	1.920821	-0.610832
O	9.581522	-0.326165	0.019131
O	-9.581524	-0.326173	0.019158
O	-7.067387	-0.384448	-0.106344
O	7.067389	-0.38443	-0.10637
N	-9.537858	2.306498	-0.320216
N	7.033464	2.263656	0.223027
N	-7.033465	2.263661	0.222879
C	10.875452	-0.243106	0.026281
C	11.556421	0.97906	-0.331016

C	10.819262	2.201845	-0.592217
C	8.869098	3.616252	-0.430664
H	8.559959	3.783835	-1.470928
H	9.534819	4.429275	-0.133064
C	-10.988879	-2.738296	0.788783
C	-10.875455	-0.243108	0.026303
C	11.565552	3.390848	-1.166223
H	12.023444	3.996392	-0.373707
H	10.906984	4.037278	-1.746804
H	12.360589	3.0595	-1.83399
C	-11.556419	0.97903	-0.331095
C	-11.656577	-1.416085	0.372676
C	13.728415	-0.117238	-0.048567
C	7.635012	3.606037	0.478745
C	13.033075	-1.298813	0.312683
H	13.628072	-2.166401	0.562618
C	-13.033083	-1.298778	0.312808
H	-13.628084	-2.166346	0.562802
C	-10.819256	2.201794	-0.592387
C	-5.008228	-1.56692	-0.367502
C	-7.635015	3.606059	0.478499
C	-13.728418	-0.117229	-0.048534
C	10.988863	-2.738357	0.788553
C	-12.962831	0.999873	-0.364608
H	-13.470225	1.920775	-0.610994
C	6.724451	4.785218	0.108401
H	7.307949	5.711057	0.136817
H	5.901775	4.912914	0.818321
H	6.310693	4.677166	-0.899968
C	-5.766181	-0.357328	-0.133999
C	-10.160306	-3.292152	-0.396072
H	-9.373633	-2.60125	-0.698969
H	-10.805123	-3.487756	-1.260687
H	-9.693766	-4.240458	-0.106902
C	-5.69874	-2.914918	-0.640046
C	-8.869092	3.616211	-0.430923
H	-9.534814	4.429256	-0.133388
H	-8.559942	3.783717	-1.471196
C	-11.565542	3.390754	-1.166487
H	-12.023448	3.996352	-0.374019
H	-12.36057	3.059355	-1.834241
H	-10.906971	4.037145	-1.747106
C	5.766184	-0.35731	-0.134023

C	10.092653	-2.510945	2.031968
H	9.635537	-3.460179	2.332567
H	9.292783	-1.795079	1.837972
H	10.688353	-2.147229	2.877504
C	5.04709	0.869873	0.072796
C	-15.261694	-0.112515	-0.066863
C	8.038923	3.683588	1.964046
H	8.729358	2.878263	2.231824
H	7.154889	3.599582	2.603909
H	8.523551	4.642492	2.175879
C	-2.902994	-0.258298	-0.117302
C	-10.09263	-2.510775	2.032149
H	-9.635515	-3.459985	2.332826
H	-10.688301	-2.146972	2.877668
H	-9.292759	-1.794936	1.838061
C	-5.047089	0.86987	0.072747
C	-3.627926	-1.461537	-0.343693
H	-3.044984	-2.351834	-0.540584
C	15.261692	-0.112515	-0.066867
C	-6.596758	-2.799535	-1.896853
H	-5.994564	-2.559917	-2.781147
H	-7.363887	-2.032273	-1.783354
H	-7.096038	-3.756925	-2.083524
C	5.74309	2.095964	0.26894
H	5.122606	2.969403	0.463616
C	12.026422	-3.815701	1.168102
H	12.676311	-4.085049	0.32788
H	11.497011	-4.7251	1.469495
H	12.654739	-3.51087	2.012808
C	-5.743091	2.095973	0.268809
H	-5.122608	2.969425	0.463427
C	-12.026445	-3.815586	1.168468
H	-11.497039	-4.724964	1.469934
H	-12.676364	-4.085006	0.328292
H	-12.654731	-3.510662	2.013163
C	10.160246	-3.292084	-0.396331
H	10.805034	-3.487613	-1.260985
H	9.373573	-2.601141	-0.699137
H	9.6937	-4.240411	-0.107241
C	-8.03894	3.683713	1.963791
H	-7.154912	3.599747	2.603668
H	-8.729379	2.878407	2.231617
H	-8.523568	4.642632	2.175554

C	-3.639631	0.898655	0.084544
H	-3.138292	1.842714	0.279911
C	3.639632	0.898655	0.084592
H	3.138291	1.842701	0.280014
C	-1.425346	-0.254956	-0.11077
C	5.008236	-1.566891	-0.36759
C	15.791934	-0.468506	1.345159
H	15.457724	-1.456683	1.677561
H	15.465191	0.267204	2.088459
H	16.887253	-0.477385	1.336797
C	-6.724451	4.785215	0.108082
H	-5.901782	4.912962	0.818
H	-7.30795	5.711056	0.136428
H	-6.310684	4.677093	-0.900276
C	2.902997	-0.25829	-0.117311
C	3.627933	-1.461516	-0.343764
H	3.044993	-2.351805	-0.540699
C	1.425349	-0.254952	-0.110774
C	-6.533023	-3.320069	0.59904
H	-5.887384	-3.441188	1.476805
H	-7.028961	-4.279604	0.414254
H	-7.297453	-2.578401	0.830825
C	-4.684301	-4.045891	-0.905548
H	-4.030746	-4.230693	-0.045036
H	-4.059201	-3.846763	-1.78365
H	-5.231415	-4.97379	-1.099989
C	5.698754	-2.914872	-0.640202
C	15.759009	-1.170279	-1.085012
H	16.854324	-1.182413	-1.098967
H	15.409573	-0.94058	-2.097705
H	15.421618	-2.180469	-0.831648
C	15.840736	1.256727	-0.469488
H	15.55481	2.047307	0.234223
H	15.529659	1.555598	-1.477412
H	16.933821	1.204277	-0.470575
C	-0.694504	-1.364581	0.354516
H	-1.219158	-2.225796	0.756878
C	6.596724	-2.799438	-1.897039
H	7.36385	-2.032172	-1.783543
H	5.994495	-2.559796	-2.781303
H	7.096007	-3.756816	-2.083762
C	-15.840735	1.256507	-0.470239
H	-15.554663	2.047506	0.232943

H	-16.933823	1.204108	-0.471134
H	-15.529801	1.554747	-1.478394
C	0.694511	-1.36458	0.354511
H	1.219168	-2.225794	0.756872
C	-0.694262	0.856243	-0.573417
H	-1.220775	1.713147	-0.983552
C	0.694261	0.856246	-0.573416
H	1.220769	1.713152	-0.983553
C	6.533092	-3.320049	0.598839
H	7.029042	-4.279567	0.414001
H	5.887488	-3.441211	1.476623
H	7.297515	-2.578374	0.830621
C	-15.758969	-1.170837	-1.084446
H	-15.40955	-0.941658	-2.097264
H	-16.854284	-1.183034	-1.098387
H	-15.42152	-2.180874	-0.830546
C	-15.79198	-0.467742	1.345339
H	-15.457834	-1.455763	1.678267
H	-16.887299	-0.476568	1.336959
H	-15.465216	0.268341	2.088259
C	4.684322	-4.045852	-0.905701
H	4.059181	-3.846704	-1.783769
H	4.030808	-4.230695	-0.045167
H	5.231443	-4.973736	-1.100199

l) Optimized XYZ Coordinates (Å) for $[\mathbf{5}^{\bullet\bullet}]^{2+}$ (S = 0)

Ni	-8.303576	0.960984	-0.034518
N	9.536817	2.306747	-0.32009
C	11.656013	-1.415028	0.372738
Ni	8.303576	0.960994	-0.034484
C	12.962469	1.000799	-0.363874
H	13.469554	1.921941	-0.610012
O	9.580463	-0.325886	0.018927
O	-9.580465	-0.325893	0.018932
O	-7.066414	-0.384585	-0.105736
O	7.066416	-0.384574	-0.105743
N	-9.536814	2.30673	-0.320168
N	7.032353	2.263584	0.221776
N	-7.032354	2.263582	0.221706
C	10.874853	-0.242416	0.026505
C	11.555638	0.979612	-0.330502
C	10.818556	2.201972	-0.591767
C	8.868192	3.616434	-0.430983

H	8.559696	3.784528	-1.471361
H	9.533674	4.429402	-0.132671
C	-10.988662	-2.737573	0.788713
C	-10.874855	-0.242419	0.026508
C	11.564635	3.391277	-1.165502
H	12.02197	3.997007	-0.372809
H	10.906128	4.037459	-1.746435
H	12.3601	3.060164	-1.832858
C	-11.555637	0.979599	-0.330539
C	-11.656018	-1.415016	0.372786
C	13.727991	-0.115849	-0.047882
C	7.633549	3.606198	0.477661
C	13.03253	-1.29747	0.313159
H	13.627565	-2.165023	0.563191
C	-13.032535	-1.297453	0.313217
H	-13.627572	-2.164996	0.563276
C	-10.818554	2.201948	-0.591843
C	-5.008252	-1.568427	-0.367279
C	-7.63355	3.606203	0.477551
C	-13.727993	-0.115842	-0.047861
C	10.98865	-2.737596	0.78862
C	-12.962469	1.00079	-0.363906
H	-13.469551	1.921925	-0.610074
C	6.722974	4.785204	0.106884
H	7.306402	5.711075	0.135502
H	5.89999	4.912923	0.816456
H	6.309703	4.677123	-0.901685
C	-5.766024	-0.358103	-0.13427
C	-10.160451	-3.29162	-0.396229
H	-9.373953	-2.600656	-0.699457
H	-10.805543	-3.487209	-1.260645
H	-9.693801	-4.239951	-0.107239
C	-5.699494	-2.916063	-0.639343
C	-8.868189	3.616414	-0.431098
H	-9.533672	4.42939	-0.132813
H	-8.559688	3.784476	-1.47148
C	-11.564631	3.391237	-1.165615
H	-12.021965	3.996992	-0.372939
H	-12.360096	3.060105	-1.83296
H	-10.906123	4.0374	-1.746568
C	5.766026	-0.358093	-0.134274
C	10.092387	-2.510575	2.032009
H	9.634996	-3.459788	2.332329

H	9.292775	-1.79435	1.838228
H	10.688128	-2.147247	2.877683
C	5.046195	0.869454	0.07112
C	-15.26137	-0.111149	-0.065741
C	8.036521	3.68363	1.963239
H	8.727025	2.878481	2.231301
H	7.152135	3.59943	2.602599
H	8.520801	4.642642	2.175329
C	-2.902517	-0.260154	-0.118396
C	-10.092324	-2.510492	2.032037
H	-9.634937	-3.459696	2.332393
H	-10.688009	-2.147096	2.87772
H	-9.292707	-1.794298	1.838165
C	-5.046195	0.86945	0.071092
C	-3.628202	-1.46359	-0.343408
H	-3.04602	-2.354364	-0.540063
C	15.261367	-0.111157	-0.065758
C	-6.597548	-2.800414	-1.896124
H	-5.99533	-2.561213	-2.780516
H	-7.364459	-2.032933	-1.782695
H	-7.097218	-3.757626	-2.082548
C	5.74213	2.096017	0.26719
H	5.12135	2.969358	0.461374
C	12.026425	-3.814726	1.168196
H	12.676505	-4.083786	0.328032
H	11.497239	-4.72432	1.46945
H	12.654578	-3.509787	2.01298
C	-5.74213	2.096017	0.267127
H	-5.121352	2.969365	0.461288
C	-12.026442	-3.814653	1.168415
H	-11.497263	-4.724241	1.469693
H	-12.676578	-4.083746	0.328305
H	-12.654539	-3.509646	2.013217
C	10.160358	-3.291548	-0.39631
H	10.805397	-3.487096	-1.260774
H	9.37386	-2.600545	-0.699449
H	9.693701	-4.239886	-0.107354
C	-8.036528	3.683677	1.963125
H	-7.152145	3.599494	2.602491
H	-8.727034	2.878537	2.231207
H	-8.520808	4.642697	2.175187
C	-3.63936	0.897699	0.082143
H	-3.137936	1.841806	0.276805

C	3.63936	0.897701	0.08217
H	3.137936	1.841802	0.276855
C	-1.425922	-0.257015	-0.111916
C	5.008257	-1.568412	-0.367308
C	15.791357	-0.466883	1.346374
H	15.457043	-1.455004	1.678867
H	15.464379	0.26893	2.089476
H	16.88669	-0.475784	1.338379
C	-6.722973	4.785199	0.106744
H	-5.899992	4.912938	0.816315
H	-7.306402	5.711071	0.135334
H	-6.309699	4.677089	-0.90182
C	2.90252	-0.260149	-0.118394
C	3.628207	-1.46358	-0.343431
H	3.046025	-2.35435	-0.540106
C	1.425924	-0.257013	-0.111914
C	-6.533903	-3.320386	0.599969
H	-5.88833	-3.441239	1.47782
H	-7.029954	-4.279908	0.415538
H	-7.298325	-2.578606	0.831351
C	-4.685634	-4.047603	-0.904613
H	-4.032246	-4.232673	-0.04402
H	-4.060435	-3.849017	-1.782779
H	-5.233265	-4.975215	-1.098894
C	5.699505	-2.916041	-0.6394
C	15.759074	-1.168903	-1.083653
H	16.854403	-1.181122	-1.097352
H	15.409887	-0.939267	-2.096453
H	15.42157	-2.179081	-0.830349
C	15.840365	1.258096	-0.46839
H	15.554087	2.048709	0.235146
H	15.529435	1.556803	-1.47641
H	16.93347	1.205844	-0.46921
C	-0.694297	-1.371739	0.341291
H	-1.218345	-2.237079	0.7353
C	6.597542	-2.800369	-1.896191
H	7.364451	-2.032886	-1.782761
H	5.995312	-2.561158	-2.780571
H	7.097214	-3.757576	-2.082636
C	-15.840365	1.258035	-0.468609
H	-15.554029	2.048782	0.234753
H	-16.933471	1.205804	-0.469355
H	-15.529493	1.556537	-1.476707

C	0.694301	-1.371738	0.34129
H	1.21835	-2.237078	0.7353
C	-0.694025	0.859337	-0.562366
H	-1.219948	1.720614	-0.963853
C	0.694025	0.859339	-0.562364
H	1.219947	1.720617	-0.96385
C	6.533936	-3.320371	0.599894
H	7.029995	-4.279885	0.415444
H	5.888375	-3.441245	1.477752
H	7.298352	-2.578586	0.831278
C	-15.759066	-1.169071	-1.083457
H	-15.409891	-0.939597	-2.096298
H	-16.854395	-1.181311	-1.097146
H	-15.42154	-2.179201	-0.829987
C	-15.791371	-0.466634	1.346448
H	-15.457082	-1.454708	1.679105
H	-16.886704	-0.475512	1.33845
H	-15.464379	0.269294	2.08943
C	4.685652	-4.047588	-0.904671
H	4.060435	-3.848994	-1.782823
H	4.032281	-4.232679	-0.044069
H	5.233289	-4.975191	-1.098977

J) TD-DFT excitation energies and oscillator strengths for $[\mathbf{2}^{\bullet\bullet}]^{2+}$ (S = 1)

```
Excited State   1:  3.021-A      0.4210 eV 2945.02 nm  f=0.0188  <S**2>=2.031
   371B -> 374B       0.13534
   372B -> 373B       0.98447
 This state for optimization and/or second-order correction.
 Total Energy, E(TD-HF/TD-KS) =  -6920.76773245
 Copying the excited state density for this state as the 1-particle RhoCI density.

 Excited State   2:  3.021-A      0.5439 eV 2279.48 nm  f=0.0704  <S**2>=2.032
   371B -> 373B      -0.43610
   372B -> 374B       0.89724

 Excited State   3:  3.021-A      0.6700 eV 1850.64 nm  f=0.3855  <S**2>=2.032
   370B -> 374B      -0.16804
   371B -> 373B       0.86365
   372B -> 374B       0.43439
   372B <- 374B      -0.12897

 Excited State   4:  3.021-A      0.7041 eV 1760.78 nm  f=0.0093  <S**2>=2.032
   371B -> 374B       0.97190
```

372B -> 373B -0.15181

Excited State 5: 3.031-A 0.8850 eV 1401.02 nm f=0.0020 <S**2>=2.046
369B -> 373B -0.10579
370B -> 373B 0.94420
370B -> 374B 0.19758
371B -> 374B 0.12835

Excited State 6: 3.041-A 0.9325 eV 1329.58 nm f=0.0228 <S**2>=2.062
370B -> 373B -0.20041
370B -> 374B 0.94300
371B -> 373B 0.19566

Excited State 7: 4.075-A 0.9530 eV 1300.93 nm f=0.0004 <S**2>=3.901
360A -> 377A 0.23405
360A -> 378A 0.10546
361A -> 377A 0.15155
362A -> 376A -0.14961
362A -> 377A 0.44233
362A -> 378A 0.19918
362A -> 379A -0.14314
362A -> 380A -0.13941
363A -> 377A -0.21988
364A -> 377A 0.10669
359B -> 378B -0.11137
360B -> 378B -0.18600
360B -> 379B 0.11924
361B -> 378B -0.13570
362B -> 373B 0.12315
362B -> 374B 0.12290
362B -> 377B 0.17970
362B -> 378B -0.46287
362B -> 379B 0.30101
362B -> 380B 0.18800
370B -> 374B -0.11774
362A <- 377A 0.13722
362B <- 378B -0.14492

Excited State 8: 4.055-A 0.9800 eV 1265.14 nm f=0.0017 <S**2>=3.861
359A -> 375A -0.30819
361A -> 375A -0.51084
361A -> 379A -0.15360
362A -> 375A 0.26226

357B -> 376B 0.20459
358B -> 376B 0.19083
361B -> 373B 0.18227
361B -> 374B -0.11824
361B -> 375B -0.15557
361B -> 376B 0.49893
361B -> 378B 0.15562
361B -> 379B 0.18619
362B -> 376B -0.19182
361A <- 375A -0.15520
361B <- 376B 0.15234

Excited State 9: 3.110-A 1.1530 eV 1075.32 nm f=0.0006 <S**2>=2.168
361A -> 375A 0.10997
357B -> 373B 0.12206
358B -> 373B 0.10830
361B -> 373B 0.60080
361B -> 374B -0.45775
362B -> 373B -0.42097
364B -> 373B -0.17491
364B -> 374B 0.17325
366B -> 373B -0.18259

Excited State 10: 3.085-A 1.1795 eV 1051.18 nm f=0.0024 <S**2>=2.129
361B -> 373B 0.34005
362B -> 373B 0.47312
362B -> 374B 0.61907
364B -> 373B -0.21477
364B -> 374B -0.11074
365B -> 373B 0.12147
365B -> 374B 0.13148
366B -> 374B 0.17332
369B -> 373B 0.11541
369B -> 374B 0.12912

K) TD-DFT excitation energies and oscillator strengths for $[\mathbf{2}^{\bullet\bullet}]^{2+}$ (S = 0)

Excited State 1: 2.218-A 0.4452 eV 2784.75 nm f=0.0552 <S**2>=0.980
373A -> 374A 0.71011
373B -> 374B 0.69105
This state for optimization and/or second-order correction.
Total Energy, E(TD-HF/TD-KS) = -6920.77136309
Copying the excited state density for this state as the 1-particle RhoCI density.

Excited State 2: 2.123-A 0.6195 eV 2001.48 nm f=0.4056 <S**2>=0.876
373A -> 374A -0.69160
373B -> 374B 0.70619
373A <- 374A 0.13201
373B <- 374B -0.12289

Excited State 3: 1.914-A 0.7691 eV 1612.07 nm f=0.0273 <S**2>=0.666
372A -> 374A -0.43178
371B -> 374B 0.13817
372B -> 374B 0.86054
373B -> 374B -0.12818

Excited State 4: 2.032-A 0.8095 eV 1531.61 nm f=0.0069 <S**2>=0.783
369A -> 374A -0.10158
371A -> 374A -0.19200
372A -> 374A 0.84771
371B -> 374B 0.13441
372B -> 374B 0.40558

Excited State 5: 3.568-A 0.9474 eV 1308.67 nm f=0.0018 <S**2>=2.933
359A -> 378A 0.21063
360A -> 378A -0.16625
362A -> 374A 0.14532
362A -> 378A -0.59087
362A -> 380A 0.28235
358B -> 375B 0.28466
361B -> 375B 0.60640
361B -> 379B -0.20897
363B -> 375B -0.12998
362A <- 378A -0.18779
361B <- 375B 0.18889

Excited State 6: 3.557-A 0.9588 eV 1293.16 nm f=0.0021 <S**2>=2.913
358A -> 375A 0.29802
361A -> 375A 0.60154
361A -> 379A 0.20191
363A -> 375A -0.10748
359B -> 378B -0.26421
359B -> 380B -0.11798
362B -> 374B 0.16696
362B -> 378B -0.58625
362B -> 380B -0.26998
361A <- 375A 0.18546

```
  362B <- 378B      -0.18416

Excited State   7:  1.776-A      1.0133 eV 1223.54 nm  f=0.0005  <S**2>=0.539
  370A -> 374A       0.10720
  371A -> 374A      -0.43397
  372A -> 374A      -0.15988
  370B -> 374B      -0.13904
  371B -> 374B       0.83108
  372B -> 374B      -0.21747

Excited State   8:  1.808-A      1.0579 eV 1172.01 nm  f=0.0136  <S**2>=0.567
  370A -> 374A      -0.12539
  371A -> 374A       0.84006
  372A -> 374A       0.16263
  371B -> 374B       0.47265

Excited State   9:  2.328-A      1.1351 eV 1092.30 nm  f=0.0003  <S**2>=1.105
  359B -> 374B       0.20665
  362B -> 374B       0.92630
  366B -> 374B      -0.17405

Excited State  10:  2.316-A      1.1472 eV 1080.72 nm  f=0.0002  <S**2>=1.091
  359A -> 374A      -0.15581
  360A -> 374A       0.12055
  362A -> 374A       0.93419
  366A -> 374A      -0.17556
```

L) TD-DFT excitation energies and oscillator strengths for [**4**••]$^{2+}$ (*S* = 1)

```
Excited State   1:  3.023-A      0.6122 eV 2025.30 nm  f=0.3971  <S**2>=2.034
  304B -> 307B       0.54697
  305B -> 306B       0.80302
  305B -> 307B      -0.10476
This state for optimization and/or second-order correction.
Total Energy, E(TD-HF/TD-KS) =  -6183.01992526
Copying the excited state density for this state as the 1-particle RhoCI density.

Excited State   2:  3.024-A      0.6859 eV 1807.51 nm  f=0.0931  <S**2>=2.036
  300B -> 306B       0.11817
  301B -> 307B      -0.10293
  304B -> 306B       0.58954
  304B -> 307B       0.16660
  305B -> 307B       0.74693
```

Excited State 3: 3.025-A 0.8799 eV 1409.10 nm f=0.0002 <S**2>=2.038
304B -> 306B -0.53582
304B -> 307B -0.45062
305B -> 306B 0.41107
305B -> 307B 0.57236

Excited State 4: 3.030-A 0.9192 eV 1348.83 nm f=0.0001 <S**2>=2.046
304B -> 306B -0.55572
304B -> 307B 0.64925
305B -> 306B -0.41299
305B -> 307B 0.28904

Excited State 5: 4.093-A 0.9655 eV 1284.18 nm f=0.0016 <S**2>=3.937
295A -> 309A 0.29401
295A -> 311A -0.12090
295A -> 313A 0.11115
297A -> 309A 0.45953
297A -> 311A -0.19120
297A -> 313A 0.17487
298A -> 309A 0.30011
298A -> 311A -0.12341
298A -> 313A 0.11280
294B -> 309B -0.22691
294B -> 311B 0.13110
294B -> 313B 0.15761
297B -> 306B 0.12949
297B -> 307B -0.11313
297B -> 309B -0.47216
297B -> 311B 0.27560
297B -> 313B 0.33216
297A <- 309A 0.14031
297B <- 309B -0.14668
297B <- 313B 0.10673

Excited State 6: 4.004-A 1.0185 eV 1217.30 nm f=0.0022 <S**2>=3.758
294A -> 308A 0.37599
294A -> 312A -0.12726
296A -> 308A -0.48467
296A -> 312A 0.16591
292B -> 308B -0.18500
293B -> 308B -0.18789
295B -> 308B -0.17188
296B -> 306B 0.20275

296B -> 307B 0.21642
296B -> 308B 0.48858
296B -> 312B -0.25284
299B -> 308B -0.14267
294A <- 308A 0.11097
296A <- 308A -0.14145
296B <- 308B 0.14383

Excited State 7: 3.167-A 1.1077 eV 1119.32 nm f=0.0050 <S**2>=2.257
294A -> 308A -0.12207
296A -> 308A 0.16053
292B -> 306B -0.12490
292B -> 307B -0.13492
293B -> 306B -0.12116
293B -> 307B -0.12734
296B -> 306B 0.54486
296B -> 307B 0.57958
296B -> 308B -0.15078
296B -> 312B 0.10358
299B -> 306B -0.20802
299B -> 307B -0.22067
300B -> 307B 0.10712
302B -> 307B -0.10504

Excited State 8: 3.075-A 1.1285 eV 1098.62 nm f=0.0004 <S**2>=2.114
294B -> 306B 0.16191
294B -> 307B -0.14472
297B -> 306B 0.68444
297B -> 307B -0.61261
298B -> 306B 0.10906
301B -> 306B 0.12216
301B -> 307B -0.10770

Excited State 9: 3.540-A 1.2703 eV 976.01 nm f=0.0679 <S**2>=2.883
292A -> 308A 0.17456
293A -> 309A 0.24045
296A -> 308A -0.10798
300A -> 309A 0.13746
302A -> 308A -0.10733
307A -> 309A 0.10737
290B -> 306B -0.21478
290B -> 307B -0.22341
290B -> 308B -0.16278

291B -> 309B -0.20907
291B -> 311B 0.12366
291B -> 313B 0.14061
296B -> 306B -0.11857
296B -> 307B -0.12569
300B -> 306B 0.27557
300B -> 307B 0.36898
301B -> 306B -0.21805
301B -> 309B 0.13292
302B -> 307B -0.11761
303B -> 306B -0.16743
304B -> 307B -0.14270

Excited State 10: 3.753-A 1.2765 eV 971.25 nm f=0.0175 <S**2>=3.272
292A -> 308A 0.16029
293A -> 309A -0.31661
293A -> 311A 0.12964
293A -> 313A -0.12164
300A -> 309A -0.18076
303A -> 309A 0.10438
306A -> 309A -0.11877
307A -> 309A -0.14283
290B -> 306B -0.16589
290B -> 307B -0.18085
290B -> 308B -0.14982
291B -> 309B 0.27151
291B -> 311B -0.15839
291B -> 313B -0.18788
300B -> 306B 0.27409
300B -> 307B 0.24167
301B -> 307B -0.14220
301B -> 309B -0.17307
301B -> 311B 0.10133
301B -> 313B 0.11308
303B -> 307B -0.11591
303B -> 309B -0.12139
304B -> 306B -0.12613

Excited State 11: 3.858-A 1.3224 eV 937.59 nm f=0.0227 <S**2>=3.472
292A -> 308A 0.41829
292A -> 312A -0.14375
302A -> 308A -0.24953
306A -> 308A 0.13896

307A -> 308A	-0.10842
290B -> 306B	0.12054
290B -> 307B	0.12421
290B -> 308B	-0.37784
290B -> 312B	0.21276
300B -> 306B	-0.17505
300B -> 307B	-0.24343
300B -> 308B	0.22764
300B -> 312B	-0.12544
301B -> 306B	0.15020
302B -> 306B	0.10942
302B -> 307B	0.11621
302B -> 308B	-0.12622
303B -> 306B	0.14271
303B -> 308B	-0.12939
304B -> 308B	0.12102

Excited State 12: 3.229-A 1.3364 eV 927.77 nm f=0.0426 <S**2>=2.357

289A -> 309A	-0.11003
307A -> 309A	-0.10780
291B -> 306B	0.27725
291B -> 307B	-0.24545
297B -> 306B	0.11248
298B -> 306B	-0.21350
298B -> 307B	0.19906
300B -> 306B	-0.21800
301B -> 306B	-0.45210
301B -> 307B	0.44096
303B -> 307B	0.11661
304B -> 306B	0.14461
305B -> 307B	0.10824

Excited State 13: 3.842-A 1.3773 eV 900.20 nm f=0.0042 <S**2>=3.440

288A -> 309A	-0.17012
289A -> 309A	0.28173
289A -> 311A	-0.11523
289A -> 313A	0.10319
291A -> 309A	0.10899
293A -> 309A	-0.25231
293A -> 311A	0.11370
303A -> 309A	0.18606
304A -> 309A	-0.17825
305A -> 309A	-0.14232

306A -> 309A 0.15906
307A -> 309A 0.17334
291B -> 309B 0.20538
291B -> 311B -0.11345
291B -> 313B -0.15322
292B -> 309B 0.12054
293B -> 309B -0.20243
293B -> 311B 0.11463
293B -> 313B 0.13933
295B -> 309B 0.10962
298B -> 309B -0.12085
301B -> 306B -0.11714
301B -> 307B 0.11531
301B -> 309B -0.12546
302B -> 309B -0.18213
302B -> 313B 0.12792
303B -> 306B -0.14239
303B -> 307B 0.11442
303B -> 309B 0.19553
303B -> 311B -0.10726
303B -> 313B -0.13569

Excited State 14: 3.685-A 1.3951 eV 888.71 nm f=0.0014 <S**2>=3.144
280A -> 308A -0.11017
288A -> 308A -0.30007
289A -> 308A -0.19968
292A -> 308A 0.11032
298A -> 308A 0.12175
301A -> 308A -0.16250
305A -> 308A -0.20045
306A -> 308A -0.29303
307A -> 308A 0.19562
290B -> 308B -0.18391
292B -> 308B 0.19189
295B -> 308B -0.19452
299B -> 308B -0.13029
300B -> 308B 0.16150
302B -> 306B 0.14154
302B -> 307B 0.18042
302B -> 308B 0.24039
302B -> 312B -0.13256
303B -> 306B 0.15832
303B -> 307B 0.13628

```
   303B -> 308B        0.21461
   303B -> 312B       -0.11793

 Excited State  15:  3.069-A      1.4070 eV  881.21 nm  f=0.0311  <S**2>=2.105
   293B -> 306B       -0.10385
   301B -> 306B       -0.10857
   302B -> 306B       -0.42790
   302B -> 307B        0.42355
   303B -> 306B        0.54630
   303B -> 307B       -0.44227
```

M) TD-DFT excitation energies and oscillator strengths for [**4**$^{\bullet\bullet}$]$^{2+}$ (S = 0)

```
Excited State   1:  2.262-A      0.6119 eV 2026.22 nm  f=0.3950  <S**2>=1.030
   305A -> 307A        0.13540
   306A -> 307A       -0.61552
   302B -> 307B       -0.10572
   305B -> 307B        0.10982
   306B -> 307B        0.73843
 This state for optimization and/or second-order correction.
 Total Energy, E(TD-HF/TD-KS) =  -6183.01985253
 Copying the excited state density for this state as the 1-particle RhoCI density.

 Excited State   2:  2.261-A      0.6906 eV 1795.31 nm  f=0.0922  <S**2>=1.028
   292A -> 307A        0.12241
   302A -> 307A       -0.14984
   305A -> 307A       -0.19172
   306A -> 307A        0.70812
   302B -> 307B       -0.12578
   305B -> 307B        0.12165
   306B -> 307B        0.61425

 Excited State   3:  3.570-A      0.9660 eV 1283.54 nm  f=0.0018  <S**2>=2.937
   294A -> 308A       -0.29499
   294A -> 310A        0.12632
   294A -> 312A       -0.10943
   296A -> 308A        0.52729
   296A -> 310A       -0.22775
   296A -> 312A        0.19639
   298A -> 308A       -0.12795
   295B -> 310B        0.22333
   295B -> 312B       -0.13329
   295B -> 313B       -0.15499
   298B -> 307B        0.17512
```

298B -> 310B -0.46991
298B -> 312B 0.28228
298B -> 313B 0.33020
296A <- 308A 0.16085
298B <- 310B -0.14590
298B <- 313B 0.10613

Excited State 4: 2.545-A 1.0145 eV 1222.15 nm f=0.0030 <S**2>=1.369
292A -> 307A -0.10153
293A -> 309A 0.13444
295A -> 309A 0.14454
297A -> 307A -0.15234
297A -> 309A -0.29907
297A -> 313A 0.15592
302A -> 307A 0.10861
305A -> 307A 0.73314
306A -> 307A 0.24115
293B -> 308B -0.22823
296B -> 308B 0.28647
305B -> 307B 0.12307

Excited State 5: 2.949-A 1.0221 eV 1213.04 nm f=0.0005 <S**2>=1.924
293A -> 309A -0.17692
295A -> 309A -0.18129
297A -> 307A 0.26279
297A -> 309A 0.36666
297A -> 313A -0.18886
299A -> 309A -0.11475
305A -> 307A 0.57664
306A -> 307A 0.12215
293B -> 308B 0.29821
296B -> 308B -0.39226
296B -> 311B 0.13294
297A <- 309A 0.10679
296B <- 308B -0.11455

Excited State 6: 1.698-A 1.0602 eV 1169.44 nm f=0.0010 <S**2>=0.470
305A -> 307A -0.14616
304B -> 307B -0.11125
305B -> 307B 0.95175
306B -> 307B -0.19331

Excited State 7: 2.467-A 1.1068 eV 1120.17 nm f=0.0045 <S**2>=1.271

293A -> 307A -0.21895
295A -> 307A -0.19987
297A -> 307A 0.77475
297A -> 309A -0.15238
297A -> 313A 0.10396
298A -> 307A -0.14244
299A -> 307A -0.31404
302A -> 307A 0.13293
303A -> 307A -0.17928
293B -> 308B -0.12868
296B -> 308B 0.17109

Excited State 8: 2.337-A 1.1270 eV 1100.12 nm f=0.0003 <S**2>=1.115
294B -> 307B -0.11095
295B -> 307B -0.21340
298B -> 307B 0.91795
299B -> 307B -0.14079
302B -> 307B -0.16766

Excited State 9: 2.998-A 1.2712 eV 975.35 nm f=0.0595 <S**2>=1.997
291A -> 308A 0.26843
291A -> 310A -0.11348
291A -> 312A 0.10112
292A -> 307A -0.28290
292A -> 309A -0.15173
297A -> 307A -0.14960
300A -> 308A 0.16954
302A -> 307A 0.43221
303A -> 307A -0.17109
305A -> 307A -0.11693
305A -> 308A -0.14968
306A -> 307A 0.10982
291B -> 308B 0.16195
292B -> 307B 0.13053
292B -> 310B -0.23353
292B -> 312B 0.14153
292B -> 313B 0.15760
298B -> 307B -0.10443
302B -> 307B -0.21519
302B -> 310B 0.15790
302B -> 313B -0.10185
303B -> 310B -0.10675

Excited State 10: 3.119-A   1.2769 eV 970.97 nm f=0.0229 <S**2>=2.181

288A -> 308A   -0.11156
291A -> 308A   -0.29057
291A -> 310A   0.12445
291A -> 312A   -0.10968
292A -> 307A   -0.27253
292A -> 309A   -0.16758
297A -> 307A   -0.14305
300A -> 308A   -0.18437
302A -> 307A   0.41384
302A -> 309A   0.10466
303A -> 307A   -0.15157
305A -> 307A   -0.10644
305A -> 308A   0.16488
306A -> 307A   0.12308
291B -> 308B   0.17874
292B -> 310B   0.24920
292B -> 312B   -0.14922
292B -> 313B   -0.17310
301B -> 308B   -0.10567
302B -> 310B   -0.16908
302B -> 312B   0.10107
302B -> 313B   0.11075
303B -> 310B   0.11928
306B -> 310B   0.10525

Excited State 11: 3.280-A   1.3229 eV 937.23 nm f=0.0237 <S**2>=2.440

292A -> 307A   0.17838
292A -> 309A   -0.37154
292A -> 313A   0.21019
302A -> 307A   -0.31382
302A -> 309A   0.22918
302A -> 313A   -0.12765
303A -> 307A   0.21098
303A -> 309A   -0.17517
306A -> 309A   -0.12687
291B -> 308B   0.41325
291B -> 311B   -0.14042
300B -> 308B   0.10009
301B -> 308B   -0.24247
302B -> 307B   0.17142
305B -> 308B   0.17339

Excited State 12: 2.515-A 1.3375 eV 926.96 nm f=0.0425 <S**2>=1.331
288A -> 308A 0.13046
302A -> 307A 0.15266
305A -> 308A -0.13148
292B -> 307B -0.37623
298B -> 307B 0.14491
299B -> 307B 0.28409
302B -> 307B 0.65826
303B -> 310B -0.11623
305B -> 307B 0.12672
306B -> 307B 0.16483

Excited State 13: 3.276-A 1.3788 eV 899.21 nm f=0.0046 <S**2>=2.433
288A -> 308A 0.32741
288A -> 310A -0.13865
288A -> 312A 0.11773
289A -> 308A 0.10370
291A -> 308A -0.25730
291A -> 310A 0.11937
298A -> 308A 0.11671
301A -> 308A 0.18264
304A -> 308A -0.23072
305A -> 308A -0.21773
292B -> 310B 0.21680
292B -> 312B -0.12405
292B -> 313B -0.16189
294B -> 310B -0.23887
294B -> 312B 0.13887
294B -> 313B 0.16459
299B -> 310B -0.11422
302B -> 307B -0.18701
302B -> 310B -0.12387
303B -> 307B 0.19528
303B -> 310B -0.26588
303B -> 312B 0.14819
303B -> 313B 0.18512

Excited State 14: 3.067-A 1.3964 eV 887.89 nm f=0.0018 <S**2>=2.102
292A -> 309A -0.18619
293A -> 309A 0.18943
295A -> 307A -0.11085
295A -> 309A -0.17613
299A -> 309A -0.13488

```
302A -> 307A       0.12856
302A -> 309A       0.16866
303A -> 307A       0.34075
303A -> 309A       0.31867
303A -> 313A      -0.17634
279B -> 308B      -0.13061
288B -> 308B      -0.35394
288B -> 311B       0.11463
291B -> 308B       0.11245
297B -> 308B       0.15090
300B -> 308B      -0.17673
304B -> 308B      -0.22529
305B -> 308B      -0.33285

Excited State  15:  2.326-A      1.4064 eV  881.58 nm  f=0.0309  <S**2>=1.103
   292B -> 307B      -0.10099
   294B -> 307B       0.16347
   303B -> 307B       0.91833
   304B -> 307B      -0.12039
```

N) TD-DFT excitation energies and oscillator strengths for [**5**••]$^{2+}$ (*S* = 1)

```
Excited State   1:  3.022-A      0.5330 eV 2326.23 nm  f=0.7111  <S**2>=2.033
   304B -> 307B       0.30214
   305B -> 306B       0.94125
 This state for optimization and/or second-order correction.
 Total Energy, E(TD-HF/TD-KS) =  -6183.02600770
 Copying the excited state density for this state as the 1-particle RhoCI density.

 Excited State   2:  3.021-A      0.6951 eV 1783.73 nm  f=0.0020  <S**2>=2.032
   291B -> 306B      -0.10331
   300B -> 306B       0.12176
   301B -> 307B      -0.11948
   304B -> 306B       0.43929
   305B -> 307B       0.86950

 Excited State   3:  3.035-A      0.9094 eV 1363.43 nm  f=0.0014  <S**2>=2.053
   300B -> 306B       0.11179
   303B -> 307B      -0.17683
   304B -> 306B       0.82645
   305B -> 307B      -0.48272

 Excited State   4:  4.024-A      0.9926 eV 1249.02 nm  f=0.0097  <S**2>=3.798
   294A -> 308A       0.25501
```

```
    295A -> 309A       -0.25341
    296A -> 308A        0.21676
    297A -> 309A       -0.35934
    297A -> 312A        0.13014
    298A -> 308A       -0.28653
    298A -> 313A       -0.10328
    293B -> 309B        0.19069
    293B -> 312B       -0.10143
    294B -> 308B       -0.20424
    294B -> 313B        0.10659
    296B -> 306B        0.15309
    296B -> 308B        0.34337
    296B -> 313B       -0.18270
    297B -> 307B        0.14496
    297B -> 309B        0.38615
    297B -> 312B       -0.20231
    299B -> 308B       -0.14183
    304B -> 307B       -0.14128
    297A <- 309A       -0.10666
    296B <- 308B        0.10461
    297B <- 309B        0.11797

 Excited State   5:  4.048-A      0.9964 eV 1244.26 nm  f=0.0001  <S**2>=3.846
    294A -> 309A       -0.25907
    295A -> 308A        0.25729
    296A -> 309A       -0.22418
    297A -> 308A        0.37282
    297A -> 313A        0.13460
    298A -> 309A        0.29856
    298A -> 312A       -0.10688
    293B -> 308B        0.19340
    293B -> 313B       -0.10181
    294B -> 309B       -0.20824
    294B -> 312B        0.10828
    296B -> 307B        0.14148
    296B -> 309B        0.34095
    296B -> 312B       -0.17987
    297B -> 306B        0.17922
    297B -> 308B        0.38144
    297B -> 313B       -0.20134
    299B -> 309B       -0.14065
    304B -> 306B        0.11524
    297A <- 308A        0.11092
```

296B <- 309B 0.10275
297B <- 308B 0.11500

Excited State 6: 3.061-A 1.0597 eV 1170.03 nm f=0.0642 <S**2>=2.092
290B -> 306B -0.14130
291B -> 307B -0.11932
296B -> 306B 0.13322
297B -> 307B 0.10769
300B -> 307B 0.20683
301B -> 306B -0.20615
303B -> 306B -0.22415
304B -> 307B 0.79944
305B -> 306B -0.32094

Excited State 7: 3.114-A 1.1199 eV 1107.11 nm f=0.0002 <S**2>=2.174
293B -> 306B 0.19453
294B -> 307B -0.17372
296B -> 307B 0.52809
297B -> 306B 0.64851
299B -> 307B -0.25507
300B -> 306B 0.19212
302B -> 306B -0.14371

Excited State 8: 3.123-A 1.1220 eV 1105.01 nm f=0.0000 <S**2>=2.188
297A -> 309A 0.10751
293B -> 307B 0.16625
294B -> 306B -0.19526
296B -> 306B 0.59354
297B -> 307B 0.55626
299B -> 306B -0.28451
300B -> 307B 0.11933
302B -> 307B -0.11862
304B -> 307B -0.23453

Excited State 9: 3.799-A 1.2837 eV 965.84 nm f=0.0234 <S**2>=3.357
292A -> 308A -0.27823
292A -> 313A -0.10161
293A -> 309A 0.29135
293A -> 312A -0.10630
300A -> 309A -0.16575
303A -> 308A -0.20896
304A -> 309A -0.12742
306A -> 309A 0.10608

307A -> 308A 0.13562
290B -> 306B -0.16880
290B -> 308B -0.26856
290B -> 313B 0.13309
291B -> 307B -0.14719
291B -> 309B -0.25549
291B -> 312B 0.12575
295B -> 309B -0.10781
296B -> 306B -0.12254
297B -> 307B -0.11619
300B -> 307B 0.21877
300B -> 309B 0.17456
301B -> 306B -0.26463
301B -> 308B -0.15368
303B -> 306B -0.11236
303B -> 308B -0.17153
304B -> 307B -0.25881
304B -> 309B 0.13591

Excited State 10: 3.867-A 1.2847 eV 965.08 nm f=0.0000 <S**2>=3.488
292A -> 309A 0.29146
292A -> 312A -0.10681
293A -> 308A -0.30485
293A -> 313A -0.11151
300A -> 308A 0.17255
303A -> 309A 0.21788
304A -> 308A 0.13339
306A -> 308A -0.10930
307A -> 309A -0.14069
290B -> 307B -0.14207
290B -> 309B -0.28354
290B -> 312B 0.14060
291B -> 306B -0.16741
291B -> 308B -0.26807
291B -> 313B 0.13348
295B -> 308B -0.11031
297B -> 306B -0.11726
300B -> 306B 0.25434
300B -> 308B 0.18363
301B -> 307B -0.21505
301B -> 309B -0.16350
303B -> 309B -0.17802
304B -> 306B -0.14404

304B -> 308B 0.14180

O) <u>TD-DFT excitation energies and oscillator strengths for $[\mathbf{5}^{\bullet\bullet}]^{2+}$ (*S* = 0)</u>

Excited State 1: 2.236-A 0.5442 eV 2278.47 nm f=0.7444 <S**2>=1.000
305A -> 307A 0.13231
306A -> 307A 0.68333
305B -> 307B -0.13231
306B -> 307B -0.68332
This state for optimization and/or second-order correction.
Total Energy, E(TD-HF/TD-KS) = -6183.02591585
Copying the excited state density for this state as the 1-particle RhoCI density.

Excited State 2: 2.170-A 0.6812 eV 1820.12 nm f=0.0002 <S**2>=0.927
302A -> 307A -0.11732
304A -> 307A -0.10519
305A -> 307A 0.17022
306A -> 307A 0.66381
302B -> 307B -0.11732
304B -> 307B -0.10519
305B -> 307B 0.17023
306B -> 307B 0.66381
306A <- 307A -0.10589
306B <- 307B -0.10589

Excited State 3: 3.521-A 0.9936 eV 1247.88 nm f=0.0069 <S**2>=2.849
293A -> 310A 0.11097
294A -> 308A 0.24461
295A -> 310A -0.17872
296A -> 308A 0.36878
296A -> 312A -0.13156
297A -> 307A 0.12564
297A -> 310A -0.32441
297A -> 313A -0.17589
298A -> 310A 0.17167
293B -> 310B 0.10857
294B -> 308B 0.24972
295B -> 310B -0.17500
296B -> 308B 0.37654
296B -> 312B 0.13432
297B -> 307B -0.12306
297B -> 310B -0.31766
297B -> 313B 0.17230
298B -> 310B -0.16813

```
   296A <- 308A       0.10961
   296B <- 308B       0.11192

Excited State   4:  3.498-A      0.9938 eV 1247.63 nm  f=0.0000  <S**2>=2.809
   293A -> 310A       0.11199
   294A -> 308A      -0.24689
   295A -> 310A      -0.17325
   296A -> 308A      -0.37450
   296A -> 312A       0.13357
   297A -> 307A       0.12260
   297A -> 310A      -0.31479
   297A -> 313A      -0.17022
   298A -> 310A       0.16512
   293B -> 310B      -0.11427
   294B -> 308B       0.24173
   295B -> 310B       0.17689
   296B -> 308B       0.36672
   296B -> 312B       0.13079
   297B -> 307B       0.12521
   297B -> 310B       0.32141
   297B -> 313B      -0.17387
   298B -> 310B       0.16861
   296A <- 308A      -0.11143
   296B <- 308B       0.10912

Excited State   5:  1.889-A      1.0558 eV 1174.28 nm  f=0.0027  <S**2>=0.642
   302A -> 307A      -0.13428
   303A -> 307A      -0.15823
   304A -> 307A      -0.25580
   305A -> 307A       0.55187
   306A -> 307A      -0.22934
   302B -> 307B      -0.13429
   303B -> 307B       0.15824
   304B -> 307B      -0.25582
   305B -> 307B       0.55190
   306B -> 307B      -0.22935

Excited State   6:  2.221-A      1.1139 eV 1113.05 nm  f=0.0584  <S**2>=0.983
   292A -> 307A      -0.10719
   293A -> 307A      -0.10949
   295A -> 307A       0.11512
   297A -> 307A       0.44515
   298A -> 307A      -0.18874
```

301A -> 307A -0.10199
302A -> 307A 0.22580
303A -> 307A 0.22272
304A -> 307A 0.13107
305A -> 307A -0.25650
306A -> 307A 0.10053
292B -> 307B 0.10718
293B -> 307B 0.10948
295B -> 307B -0.11511
297B -> 307B -0.44511
298B -> 307B -0.18872
301B -> 307B 0.10199
302B -> 307B -0.22579
303B -> 307B 0.22270
304B -> 307B -0.13106
305B -> 307B 0.25648
306B -> 307B -0.10052

Excited State 7: 2.363-A 1.1279 eV 1099.25 nm f=0.0001 <S**2>=1.146
293A -> 307A -0.11107
295A -> 307A 0.15956
297A -> 307A 0.54402
298A -> 307A -0.27292
301A -> 307A -0.10150
303A -> 307A 0.11898
305A -> 307A 0.11353
293B -> 307B -0.11107
295B -> 307B 0.15957
297B -> 307B 0.54405
298B -> 307B 0.27293
301B -> 307B -0.10150
303B -> 307B -0.11899
305B -> 307B 0.11352

Excited State 8: 2.132-A 1.1546 eV 1073.86 nm f=0.0858 <S**2>=0.886
292A -> 307A 0.14560
295A -> 307A 0.12051
296A -> 308A -0.12415
297A -> 307A 0.33310
298A -> 307A -0.20556
302A -> 307A -0.16887
304A -> 307A -0.17784
305A -> 307A 0.43031

306A -> 307A -0.13073
292B -> 307B -0.14560
295B -> 307B -0.12050
296B -> 308B -0.12415
297B -> 307B -0.33310
298B -> 307B -0.20556
302B -> 307B 0.16885
304B -> 307B 0.17783
305B -> 307B -0.43028
306B -> 307B 0.13072

Excited State 9: 3.357-A 1.2907 eV 960.58 nm f=0.0001 <S**2>=2.567
291A -> 308A 0.31113
291A -> 312A -0.11241
292A -> 307A -0.13211
292A -> 310A 0.27371
292A -> 313A 0.14025
301A -> 308A 0.18590
302A -> 307A 0.19852
302A -> 310A -0.16867
303A -> 310A -0.12215
305A -> 307A 0.11674
291B -> 308B -0.31136
291B -> 312B -0.11248
292B -> 307B -0.13208
292B -> 310B -0.27343
292B -> 313B 0.14015
301B -> 308B -0.18603
302B -> 307B 0.19850
302B -> 310B 0.16849
303B -> 310B -0.12201
305B -> 307B 0.11665

Excited State 10: 3.412-A 1.2951 eV 957.36 nm f=0.0025 <S**2>=2.660
291A -> 308A 0.32257
291A -> 312A -0.11711
292A -> 310A -0.28251
292A -> 313A -0.14794
301A -> 308A 0.18874
302A -> 307A -0.10925
302A -> 310A 0.17639
303A -> 310A 0.13057
305A -> 307A -0.17019

305A -> 308A	0.10112
291B -> 308B	0.32236
291B -> 312B	0.11704
292B -> 310B	-0.28260
292B -> 313B	0.14804
301B -> 308B	0.18862
302B -> 307B	0.10942
302B -> 310B	0.17645
303B -> 310B	-0.13061
305B -> 307B	0.17028
305B -> 308B	0.10106

References

1. Han, S.; Yao, E.; Qin, W.; Zhang, S.; Ma, Y., *Macromolecules* 2012, **45**, 4054.
2. Dunn, T. J.; Ramogida, C. F.; Simmonds, C.; Paterson, A.; Wong, E. W. Y.; Chiang, L.; Shimazaki, Y.; Storr, T., *Inorg. Chem.* 2011, **50**, 6746.
3. Murata, Y.; Cheng, F.; Kitagawa, T.; Komatsu, K., *J. Am. Chem. Soc.* 2004, **126**, 8874.
4. Noviandri, I.; Brown, K. N.; Fleming, D. S.; Gulyas, P. T.; Lay, P. A.; Masters, A. F.; Phillips, L., *J. Phys. Chem. B* 1999, **103**, 6713.
5. Stoll, S.; Schweiger, A., *J. Magn. Reson.* 2006, **178**, 42.
6. Sheldrick, G. M. *SHELXT v2014*, Bruker AXS Inc: Madison, WI.
7. Hubschle, C. B.; Sheldrick, G. M.; Dittrich, B., *J. Appl. Crystallogr.* 2011, **44**, 1281.
8. van der Sluis, P.; Spek, A. L., *Acta Crystallographica Section A* 1990, **46**, 194.
9. Farrugia, L., *J. Appl. Crystallogr.* 2012, **45**, 849.
10. *Persistance of Vision Raytracer (POV-Ray)*, 3.6.2; Persistence of Vision Pty. Ltd.: Victoria, Australia, 2004.
11. Frisch, M. J.*, et al. Gaussian 09*, Gaussian, Inc.: Wallingford, CT, USA, 2009.
12. Becke, A. D., *J. Chem. Phys.* 1993, **98**, 5648.
13. Stephens, P. J.; Devlin, F. J.; Chabalowski, C. F.; Frisch, M. J., *J. Phys. Chem.* 1994, **98**, 11623.
14. Dunn, T. J.; Chiang, L.; Ramogida, C. F.; Hazin, K.; Webb, M. I.; Katz, M. J.; Storr, T., *Chem. Eur. J.* 2013, **19**, 9606.
15. Dunn, T. J.; Chiang, L.; Ramogida, C. F.; Webb, M. I.; Savard, D.; Sakaguchi, M.; Ogura, T.; Shimazaki, Y.; Storr, T., *Dalton Trans.* 2012, **41**, 7905.
16. Clarke, R. M.; Hazin, K.; Thompson, J. R.; Savard, D.; Prosser, K. E.; Storr, T., *Inorg. Chem.* 2016, **55**, 762.
17. Schäfer, A.; Horn, H.; Ahlrichs, R., *J. Chem. Phys.* 1992, **97**, 2571.
18. Schäfer, A.; Huber, C.; Ahlrichs, R., *J. Chem. Phys.* 1994, **100**, 5829.
19. Casida, M. E., In *Recent Advances in Density Functional Methods*, Chong, D. P., Ed. World Scientific: Singapore, 1995; p 155.
20. Stratmann, R. E.; Scuseria, G. E.; Frisch, M. J., *J. Chem. Phys.* 1998, **109**, 8218.
21. Barone, V.; Cossi, M.; Tomasi, J., *J. Comput. Chem.* 1998, **19**, 404.
22. Barone, V.; Cossi, M.; Tomasi, J., *J. Chem. Phys.* 1997, **107**, 3210.
23. Miertuš, S.; Scrocco, E.; Tomasi, J., *Chem. Phys.* 1981, **55**, 117.

24. Tomasi, J.; Mennucci, B.; Cancès, E., *J. Mol. Struct. THEOCHEM* 1999, **464**, 211.
